# Diversity of short interspersed nuclear elements (SINEs) in lepidopteran insects and evidence of horizontal SINE transfer between baculovirus and lepidopteran hosts

**DOI:** 10.1186/s12864-021-07543-z

**Published:** 2021-03-31

**Authors:** Guangjie Han, Nan Zhang, Heng Jiang, Xiangkun Meng, Kun Qian, Yang Zheng, Jian Xu, Jianjun Wang

**Affiliations:** 1 College of Horticulture and Plant Protection, Yangzhou University, Yangzhou, 225009 China; 2 Jiangsu Lixiahe District Institute of Agricultural Sciences, Yangzhou, 225008 China; 3 Joint International Research Laboratory of Agriculture andAgri-Product Safety of the Ministry of Education, Yangzhou University, Yangzhou, 225009 China

**Keywords:** Short interspersed nuclear element (SINE), Horizontal transfer, *Plutella xylostella*, Retrotransposon, Long interspersed nuclear elements (LINEs)

## Abstract

**Background:**

Short interspersed nuclear elements (SINEs) belong to non-long terminal repeat (non-LTR) retrotransposons, which can mobilize dependent on the help of counterpart long interspersed nuclear elements (LINEs). Although 234 SINEs have been identified so far, only 23 are from insect species (SINEbase: http://sines.eimb.ru/).

**Results:**

Here, five SINEs were identified from the genome of *Plutella xylostella*, among which *PxSE1*, *PxSE2* and *PxSE3* were tRNA-derived SINEs, *PxSE4* and *PxSE5* were 5S RNA-derived SINEs. A total of 18 related SINEs were further identified in 13 lepidopteran insects and a baculovirus. The 3′-tail of *PxSE5* shares highly identity with that of LINE retrotransposon, PxLINE1. The analysis of relative age distribution profiles revealed that *PxSE1* is a relatively young retrotransposon in the genome of *P. xylostella* and was generated by recent explosive amplification. Integration pattern analysis showed that SINEs in *P. xylostella* prefer to insert into or accumulate in introns and regions 5 kb downstream of genes. In particular, the *PxSE1*-like element, *SlNPVSE1*, in *Spodoptera litura* nucleopolyhedrovirus II genome is highly identical to *SfSE1* in *Spodoptera frugiperda*, *SlittSE1* in *Spodoptera littoralis*, and *SlituSE1* in *Spodoptera litura*, suggesting the occurrence of horizontal transfer.

**Conclusions:**

Lepidopteran insect genomes harbor a diversity of SINEs. The retrotransposition activity and copy number of these SINEs varies considerably between host lineages and SINE lineages. Host-parasite interactions facilitate the horizontal transfer of SINE between baculovirus and its lepidopteran hosts.

**Supplementary Information:**

The online version contains supplementary material available at 10.1186/s12864-021-07543-z.

## Background

Short interspersed nuclear elements (SINEs) are Class I transposable elements (TEs) that propagate by a copy-and-paste mechanism [1, 2]. SINEs are evolutionarily derived from endogenous RNA polymerase III (Pol III) transcripts [3]. While mammalian SINEs, such as *B1* and *Alu*, are originated from 7SL RNAs, other eukaryotes primarily harbor tRNA-like SINEs [4], and SINEs originated from 5S rRNA have been found in zebrafish, fruit bats, and springhare [5, 6]. Recently, SINEs derived from small nuclear RNA (*snRNA*) (*SINEU*) and the 3′-end of the large ribosomal subunit (LSU or 28S rDNA) (*SINE28*) have been identified in avian, crocodilian and mammalian genomes, respectively [7–9]. The characteristic features of SINEs include a 5′ terminal RNA-related region which contains an internal Pol III promoter, a central region, and a 3′-tail that is of variable length and recognized by the reverse transcriptase (RT) of autonomous partner long nuclear interspersed element (LINE*)* during retrotransposition [3]. The SINEs promoters originated from tRNA and 7SL RNA comprise box A and B motif, whereas 5S rRNA-derived SINE promoters have three boxes such as A, IE and C [10].

As non-autonomous retrotransposon, the replication rate and survival of a SINE is dependent on the partner LINE activity, and its genomic copy number varies greatly between families and host species. For example, as high as 1 million copies of *Alu* elements have been identified in the human genome [11], whereas only two copies of *ZmSINE3* were detected in *Zea mays* [12]. On the other hand, the number of SINE families within a genome is also highly variable, ranging from a single SINE family in the Vitaceae to 22 SINE families in the Amaranthaceae [13]. Interestingly, unlike LINEs, the distribution of a SINE family is generally restricted to a certain taxonomic group such as orders/families [3, 4, 14], suggesting that SINEs are one of the major genetic elements that determine a clade-specific genomic composition.

Transposable elements play an important role in the epigenetic regulation of the genome and generation of genomic novelty. A growing body of evidence has recently accumulated indicating that SINEs have a deep impact on genome organization and gene structure by generating regulatory elements for gene expression [15, 16], exon skipping and alternative splicing [17], alternative polyadenylation signals [18, 19], and even functional RNA genes [20, 21]. For example, an *Alu* SINE inserted into human pluripotency-associated transcript 5 (HPAT5) regulated related microRNAs through its let-7 binding site, which is essential for inner cell mass formation during early embryonic development [22].

While SINEs have been well characterized in human [23], other mammals [24] and plants [25], and currently about 200 SINE families/subfamilies are identified in various clades in Metazoa, as reported in Repbase [26] and in SINEBase [2], information on insect SINEs is still limited [27–33]. Recent improvements in both genome sequencing and assembly methodologies have led to increasing high-quality insect genome assemblies, which provides the opportunity to identify novel SINEs. However, due to their minimal sequence feature, the lack of coding capacity, and high sequence heterogeneity, annotations of SINE are often incomplete or missing. Here, we described three tRNA-derived SINE families and two 5S rRNA-derived SINE families in the diamondback moth (DBM), *Plutella xylostella* (L.), which is one of the most damaging insect pests of cruciferous vegetables around the world. We investigated the structures and insertion regions of these SINEs. The distribution of these SINEs in other lepidopteran insect species was also surveyed.

## Results

### Novel tRNA-derived SINE retrotransposons, *PxSE1*, *PxSE2* and *PxSE3*, in *P. xylostella*

**Fig. 1:**
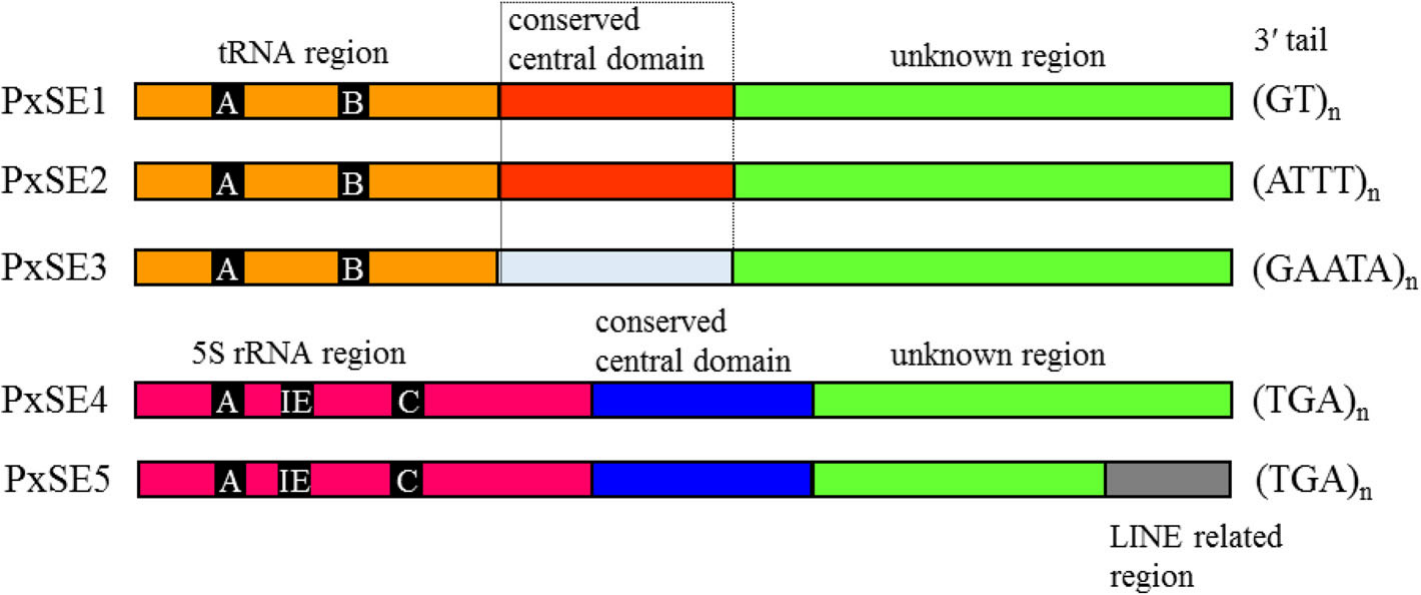
The schematic representation of structure of *PxSE1*, *PxSE2*, *PxSE3*, *PxSE4* and *PxSE5* in *P. xylostella*. The A, B, IE and C in tRNA^Arg^ or 5S rRNA region represent A box, B box, intermediate element and C box, respectively.The *PxSE1*, *PxSE2* and *PxSE3* are tRNA-derived SINEs, *PxSE4* and *PxSE5* are 5S rRNA-derived SINEs

**Fig. 2:**
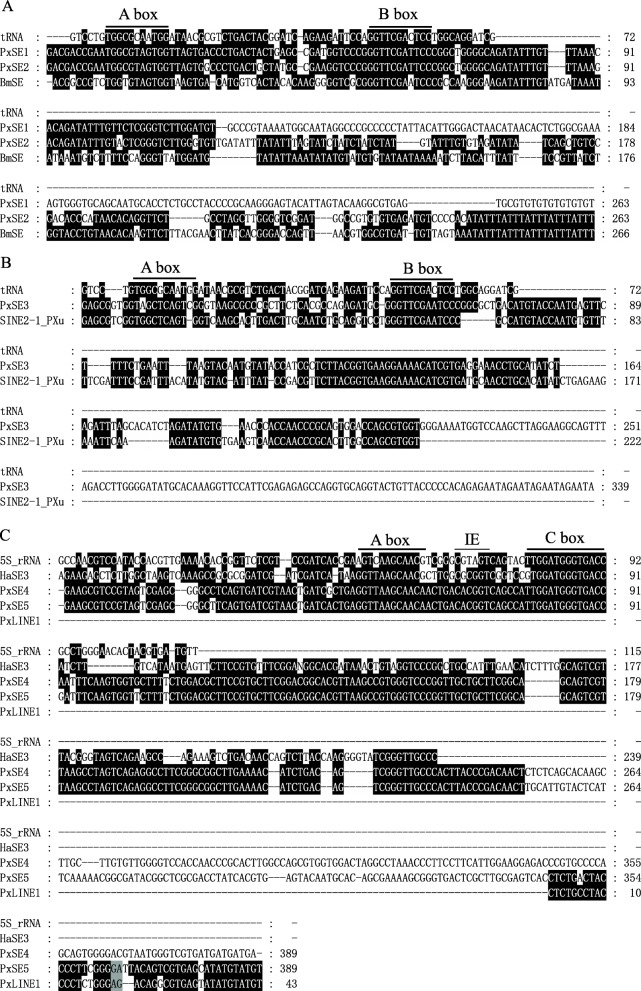
The consensus sequences of *PxSE1*, *PxSE2*, *PxSE3*, *PxSE4* and *PxSE5*. *BmSEm*, *SINE2-1_PXu* and *HaSE3* sequences were obtained from Repbase database. tRNA and 5S rRNA sequences were downloaded from *D. melanogaster* tRNA^Arg^ sequence (Accession number: V00243) and *B. mori* (Accession number: K03316), respectively. **a** *PxSE1* and *PxSE2* consensus sequences aligned with tRNA sequence and *BmSE*. Nucleotides shaded in black are conserved across sequences. The underlined sequences of A Box and B Box are the RNA pol III promoter sequences. **b** *PxSE3* consensus sequence aligned with tRNA-related region and conserved central domain of *SINE2-1_PXu*. **c** *PxSE4*, *PxSE5* consensus sequences aligned with 5S rRNA and 3′-region of *PxLINE1.1*. *PxLINE1.1* is a new LINE transposon in *P. xylostella*

A novel tRNA-derived SINE, *PxSE1*, was identified by homology search in DBM genome database (Additional file 1: Figure S1). A total of 68 full length copies homologous to *PxSE1* were used to reconstruct the consensus sequence of *PxSE1* (Accession numbers: MW068006-MW068073). The *PxSE1* is 263 bp long, includes GT dinucleotide repeats at 3′-tail and a 72-bp tRNA-related region at the 5′-end with 64% identity to 72-bp tRNA^Arg^ of *Drosophila melanogaster*, which contains box A and box B of the RNA Pol III promoter (Figs. 1 and 2a, Additional file 2: Figure S2). The boundary of *PxSE1* was further defined by the alignment of a *PxSE1* element and its empty site sequence (Additional file 3: Figure S3). Using the *PxSE1* as the query, a total of 6208 copies were identified in DBM genome (Table 1). The average divergence is 0.035 in all *PxSE1* copies (Table 1), indicating a recent invasion time.

**Table 1:** Novel SINE elements identified in this study

SINE Family	Species	RNA Origin	Consensus Length	Tail	Copy Number^a^	Divergence	Resource
PxSE1	*Plutella xylostella*/Yponomeutoidea	tRNA	263	(GT)n	6208 (68)	0.035	WGS
MsSE1	*Manduca sexta*/Bombycoidea	tRNA	267	(GT)n	7513 (133)	0.091	WGS
SfSE1	*Spodoptera frugiperda/*Noctuoidea	tRNA	298	(ATGT)n	11,117 (79)	0.130	WGS
SlNPVSE1	*Spodoptera litura* nucleopolyhedrovirus II	tRNA	260	(TGTTA)n	1 (1)	ND	Nr/nt
SlituSE1	*Spodoptera litura*/Noctuoidea	tRNA	259	(ATGTT)n	ND(8)	ND	EST
SlittSE1	*Spodoptera littoralis*/Noctuoidea	tRNA	270	(ATGTT)n	ND(15)	ND	EST
CfSE1	*Choristoneura fumiferana*/Tortricoidea	tRNA	252	(ATTT)n	ND(10)	ND	EST
PxSE2	*P. xylostella*	tRNA	263	(ATTT)n	5056 (33)	0.071	WGS
ObSE1	*Operophtera brumata*/Geometroidea	tRNA	275	(TATT)n	4521 (120)	0.066	WGS
CsSE1	*Chilo suppressalis/*Pyraloidea	tRNA	287	(TATT)n	533 (125)	0.036	WGS
PxSE3	*P. xylostella*	tRNA	339	(GAATA)n	5158 (50)	0.089	WGS
MsSE2	*M. sexta*	tRNA	333	(TAT)n	16,157 (126)	0.090	WGS
PgSE1	*Papilio glaucus*/Papilionoidea	tRNA	303	(TAT)n	1751 (66)	0.097	WGS
PmSE1	*Papilio machaon/*Papilionoidea	tRNA	310	(GAT)n	5740 (138)	0.098	WGS
LaSE1	*Lerema accius/*Hesperioidea	tRNA	319	(GAT)n	3832 (52)	0.107	WGS
PzSE1	*Papilio zelicaon*/Papilionoidea	tRNA	293	(GAT)n	ND(15)	ND	TSA
EpSE1	*Erynnis propertius*/Hesperioidea	tRNA	300	ND	ND(8)	ND	TSA
SeSE1	*Spodoptera exigua/*Noctuidae	tRNA	291	(GAT)n	ND(43)	ND	TSA
PxSE4	*P. xylostella*	5S rRNA	389	(TGA)n	4415 (50)	0.078	WGS
LaSE2	*L. accius*	5S rRNA	348	ND	1214 (45)	0.101	WGS
CsSE2	*C. suppressalis*	5S rRNA	294	(TGA)n	532 (169)	0.021	WGS
ObSE2	*O. brumata*	tRNA	255	(CGAAA)n	863 (65)	0.012	WGS
PxSE5	*P. xylostella*	5S rRNA	389	(ATGT)n	1952 (23)	0.132	WGS

*ND* not determined

^a^ the number in bracket is the number of copies used to reconstruct the consensus sequences

Using *PxSE1* as query, two additional tRNA-derived SINEs, *PxSE2* and *PxSE3*, were identified by database searches. The consensus sequences of *PxSE2* and *PxSE3* were reconstructed using the same methods as described above (Accession numbers: MW068074-MW068156, Additional file 2: Figure S2 and Additional file 3: Figure S3). The *PxSE2* is 263 bp long, includes a 143 bp 3′-end sequence, which is different from *PxSE1*, but has 67.5% identity with *BmSE*. The 72-bp tRNA-related region of *PxSE2* is 93.4% identical to *PxSE1* (Figs. 1 and 2a). Interestingly, *PxSE2* has a 44 bp conserved central domain with 93.2% identity to *PxSE1* (Fig. 2a). The *PxSE3* is 339 bp long, includes a 72-bp tRNA-related region with 66.5% identity to tRNA^Arg^ of *D. melanogaster*, and has 79.3% identity with the 222 bp sequence at 5′-end of *SINE2-1_Pxu* from *Papilio xuthus* [34] (Figs. 1 and 2b). The copy numbers of *PxSE2* and *PxSE3* were 5056 and 5158 in DBM genome, respectively (Table 1). The average divergence of *PxSE2* and *PxSE3* were 0.071 and 0.089, respectively (Table 1).

### Distribution of *PxSE1*, *PxSE2* and *PxSE3* in other species

BLAST searches were performed to detect *PxSE1*, *PxSE2* and *PxSE3* sequences in insect species other than *P. xylostella*. In total, homologous sequences of *PxSE1*, *PxSE2* and *PxSE3* were identified in five, two and seven Lepidoptera insects, respectively (Accession numbers: MW068230-MW069451, Additional file 2: Figure S2), among which *MsSE2* in *Manduca sexta* showed the highest copy numbers of 16,157, whereas only 533 copies of *CsSE1* were detected in the genome of *Chilo suppressalis* (Table 1). The consensus sequences of these elements vary in size from 252 bp to 333 bp and have different 3′-tails. Differently, the consensus sequences of *EpSE1* did not contain poly(A), poly(T) or simple sequence repeats at 3′-end. The average divergence varied from 0.035 to 0.13 (Table 1). Although *PxSE2*- and *PxSE3*-like elements were not identified in non-insect species, a *PxSE1*-like element, *SlNPVSE1*, was detected in *Spodoptera litura* nucleopolyhedrovirus II (EU780426.1: 30485–30735), which was located within ORF27 encoding an unknown protein.

Multiple sequence alignment of the consensus sequences showed that the evolutionary divergence varied from 0.003 to 0.436. The highest identity (99.7%) was observed between *PmSE1* in *Papilio machaon* and *PzSE1* in *Papilio zelicaon*, whereas *MsSE1* in *M. sexta* and *CfSE1* in *Choristoneura fumiferana* showed the highest evolutionary divergence (0.436) (Additional file 4: Figure S4).

### Two 5S rRNA-derived SINEs, *PxSE4* and *PxSE5*, in *P. xylostella* and related species

Using *HaSE3* as a query [33], BLAST searches revealed two 5S rRNA-derived SINEs, *PxSE4* and *PxSE5*, in DBM (Accession numbers: MW068157-MW068229, Figs. 1 and 2c). The boundary of *PxSE4* and *PxSE5* was further defined by the alignment of single PxSE element and its empty site sequence (Additional file 2: Figure S2 and Figure S3). *PxSE4* and *PxSE5* are both 389 bp in length and shared high identity of 250 bp sequence at 5′-end but are different at the 3′-end. The promoter regions of *PxSE4* and *PxSE5* include the specific A box, IE and C boxes, and shared about 63% identity with 5S rRNA of *Bombyx mori*, indicating that they are 5S rRNA-derived SINEs (Fig. 2c). The copy numbers and average divergence of *PxSE4* and *PxSE5* were 4415 and 1952, 0.078 and 0.132, respectively (Table 1).

**Fig. 3:**
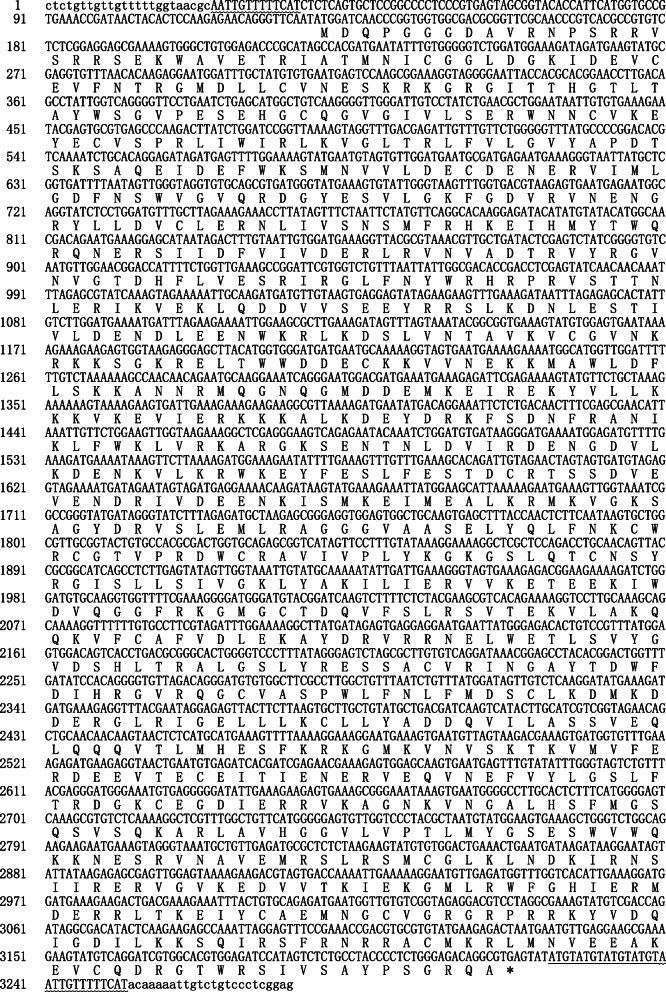
The nucleotide sequence and conceptual translation of the partner LINE element, *PxLINE1.1*, for *PxSE5*. Flanking direct repeats are indicated in lowercase. The nucleotides of TSD are indicated with the wavy line. The nucleotides of 3′ tail sequence are indicated with the straight line

Interestingly, we found a LINE element *PxLINE1.1* (NW_011952036.1: 552486–555,713) with its 43-bp 3′-end being 84% identical to that of *PxSE5* (Fig. 2). Thus, this region was designated as 3′-LINE-related region (Fig. 1). The *PxLINE1.1* element was 3228 bp long, flanked by 13 bp target site duplications (TSDs), encoded L1_EN (Endonuclease domain of the non-LTR retrotransposon LINE-1) and RT domain, and was terminated by ATGT tetranucleotide repeats in the short 3′ untranslated region (3′ UTR) (Fig. 3). Additional eight copies were found to be 96.1 to 99.7% identical to *PxLINE1.1* in *P. xylostella.* Specifically, one copy (AHIO01028576.1:13049_14357) from WGS was inserted as a 1686 bp fragment, which shared 71.8% identity with *mariner-8_BM* from *B. mori* [35] (Table 2 and Additional file 5: Figure S5). Sequences sharing 63 to 82% identity with the 1580 bp fragments at the 3′-end of *PxLINE1.1* were also found in the other 7 lepidopteran insect genomes (Additional file 6: Figure S6).

**Table 2:** Copies with high identity to *PxLINE1.1* in *P. xylostella*

Subject No.	Identity %	Length	Start	End	Evalue	Bit score
AHIO01036046.1	99.6	3203	31,532	34,758	0	5838
AHIO01028688.1	99.5	3203	1034	4236	0	5819
AHIO01028673.1	99.4	3203	10,866	7665	0	5810
AHIO01016682.1	99.3	3203	4885	8087	0	5797
AHIO01031207.1	99.2	3207	51	3252	0	5784
AHIO01033561.1	99	3209	7965	4750	0	5749
AHIO01003557.1	96.1	3214	43,696	40,488	0	5243
AHIO01028576.1	99.7	4408	11,375	15,783	0	5067

The *PxSE4* and *PxSE5* sequences were used as queries to search against the whole genome shotgun (WGS) and expressed sequence tags (EST) database using BLASTN. Three elements, *LaSE2*, *CsSE2* and *ObSE2*, with high identities to *PxSE4* were found in genomes of *Lerema accius, C. suppressalis* and *Operophtera brumata*, respectively (Accession numbers: MW068230-MW069451, Additional file 2: Figure S2). In particular, the 115-bp fragment at 5′-end of *ObSE2* is different from *PxSE4*, whereas the central 122-bp fragment shares highly identity with *PxSE4* (Additional file 4: Figure S4G and Additional file 7: Figure S7B). The 75-bp fragment at 5′-end of *ObSE2* is 54.2% identical to the 72-bp tRNA of *D. melanogaster*, but different from *PxSE1* (Additional file 7: Figure S7A). However, no simple repeat sequences were found at the 3′-ends of the *ObSE2*. While we did not find *PxSE5*-like elements in other insects, the 56-bp fragment at 3′-end of *PxSE5* and *SfSE1* shared 89.6% identity (Additional file 4: Figure S4I).

### Transpositional burst of SINEs

**Fig. 4:**
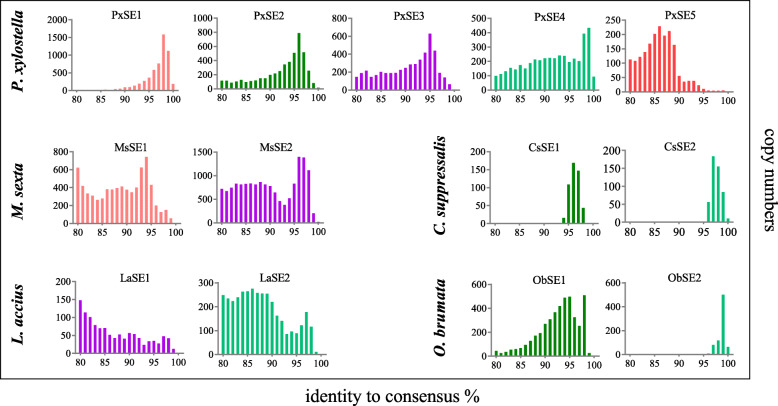
Examples for the relative age distribution of SINE families in *P. xylostella*, *M. sexta*, *C. suppressalis*, *L. accius* and *O. brumata*. The abscissa showed the identities between each consensus sequence and the copies. The ordinate showed the copy numbers of sequence with the same identity. The same color represented the same family of SINE

Due to the accumulation of random mutations over time, evolutionarily ancient SINE families have a lower sequence identity among copies, whereas SINEs families with recent or ongoing transposition harbor relatively homogeneous copies [12]. To evaluate the periods of transpositional activity and relative age of SINE copies per family of SINEs, we performed a pairwise comparison of SINE copies with the consensus sequences of respective family and grouped them into intervals from 80 to 100% identity. As shown in Fig. 4, 4796 of 6208 copies of *PxSE1* show more than 95% identity to the consensus sequence, of which 223 copies are 100% identical to *PxSE1* consensus sequence (Additional file 8: Table S1), indicating a recent transpositional burst. A strong transposition peak with high identity values is also observed in *PxSE2*, *PxSE3*, and *PxSE4*. However, *PxSE5* shows high numbers of diverged copies, and only 49 copies (2.5%) of *PxSE5* have more than 95% identity with its consensus sequence (Fig. 4).

The activity profiles deduced from similarity intervals of SINEs in other lepidopteran species revealed a recent transpositional burst of *CsSE1* and *CsSE2* in *C. suppressalis* and *ObSE2* in *O. brumata*, whereas *LaSE1* and *SfSE1* harbour diverged copies and only few young ones (Fig. 4 and Additional file 9: Figure S8). High number of copies with a wide range of identity values were observed in *MsSE1*, *MsSE2*, *PgSE1*, *PmSE1* and *LaSE2* (Fig. 4 and Additional file 9: Figure S8). Due to few copies in related EST and transcriptome shotgun assembly (TSA) databases, the distribution profiles of copy identity in *SlituSE1*, *SlittSE1*, *CfSE1*, *PzSE1*, *EpSE1* and *SeSE1* were not subject to analysis.

### Contribution of SINEs to gene and genome evolution in *P. xylostella*

**Fig. 5:**
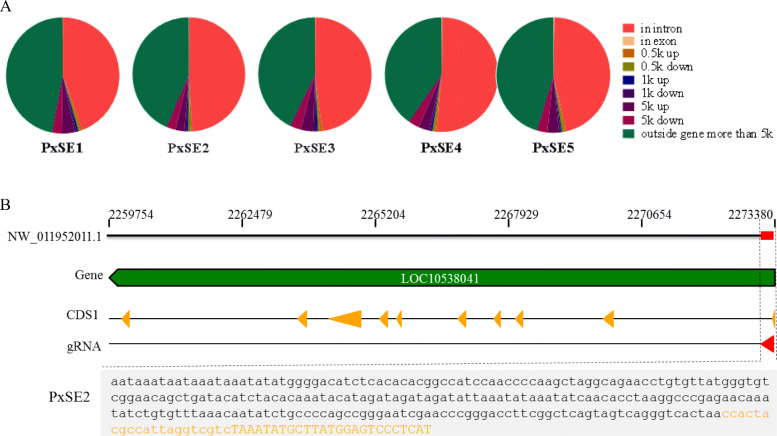
Gene association of SINEs in *P. xylostella*. **a** Overall proportions of SINEs in the genome of in *P. xylostella* are represented as pie charts. **b** Integration of a *PxSE2* element within the CDS of a gene encoding a nitrogen permease regulator 3-like protein. The sequences with yellow represent the exon region of LOC105380419, the sequences with lowercase is a *PxSE2.2* copy of *PxSE2*

The integration pattern relative to the annotated genes in the genome of *P. xylostella* was analyzed. A total of 2750 out of 6208 copies (44%) of *PxSE1*, 2478 out of 5056 copies (49%) of *PxSE2*, 2470 out of 5158 copies (48%) of *PxSE3*, 2265 out of 4415 copies (51%) of *PxSE4* and 902 out of 1952 copies (46%) of *PxSE5* were found in introns (Fig. 5a). Similar proportions of the copies are distributed in regions 5kbp downstream of genes. Only two, five, five, eight and five copies of *PxSE1*, *PxSE2*, *PxSE3*, *PxSE4* and *PxSE5* were found to insert into exonic regions, respectively (Fig. 5a). Among them, 11 copies are inserted into the coding regions (CDS), a copy is inserted into the 5′ UTR, and 13 copies are inserted into the 3′ UTR (Table 3). Most of these genes were annotated as enzymes or enzyme-associated proteins, and were related to signal transduction, splicing, metabolism. For example, a 261 bp copy *PxSE2.2* of *PxSE2* family from DBM genome (NW_011952011.1: 2273356–2273095) inserted into CDS of a gene encoding nitrogen permease regulator 3-like protein. The 21-bp fragment at 5′-end of *PxSE2.2* contributed 7 amino acids to the N-terminus of the protein (Fig. 5b).

**Table 3:** The annotation of SINEs copies integrated into CDS and untranslated regions (UTR) in *P. xylostella*

Copies	Location	GeneID	Gene mapping	COG class annotation	Swissprot annotation	Nr annotation
PxSE1.1	436,420–436,680	LOC105383591	CDS	Signal transduction mechanisms	Cyclic nucleotide-gated cation channel subunit A	uncharacterized protein
PxSE1.2	46,181–46,441	LOC105394666	CDS	Signal transduction mechanisms	Cyclic nucleotide-gated cation channel subunit A	uncharacterized protein
PxSE2.1	800,671–800,788	LOC105384210	3′ UTR	ND	Transcription factor 25 homolog	transcription factor 25
PxSE2.2	2,273,356–2,273,095	LOC105380419	CDS	ND	Nitrogen permease regulator 3-like protein	nitrogen permease regulator 3-like protein
PxSE2.3	35,405–35,544	LOC105390425	3′ UTR	ND	Serine/threonine-protein kinase	serine/threonine-protein kinase grp-like
PxSE2.4	250,511–250,756	LOC105381765	CDS	Replication, recombination and repair	DNA topoisomerase 3-alpha	uncharacterized protein
PxSE2.5	266,277–266,424	LOC105391817	3′ UTR	ND	Leucine-rich repeat serine/threonine-protein kinase 1	uncharacterized protein
PxSE3.1	274,387–274,287	LOC105388973	3′ UTR	ND	Gamete and mating-type specific protein A	uncharacterized protein
PxSE3.2	699,462–699,145	LOC105386775	CDS	ND	Uncharacterized protein	serine/arginine repetitive matrix protein 1-like
PxSE3.3	490,234–490,329	LOC105381296	3′ UTR	ND	Venom acid phosphatase Acph-1 (Precursor)	prostatic acid phosphatase
PxSE3.4	149,472–149,747	LOC105389005	3′ UTR	General function prediction only	Protein suppressor of hairy wing	zinc finger protein 26-like
PxSE3.5	165,558–165,422	LOC105383366	CDS	ND	Mediator of RNA polymerase II transcription subunit 12	uncharacterized protein
PxSE4.1	1,260,998–1,260,894	LOC105380733	3′ UTR	General function prediction only	Ras-related protein Rab-24	ras-related protein Rab-24-like
PxSE4.2	63,533–63,636	LOC105391976	3′ UTR	General function prediction only	Protein fem-1 homolog B	protein fem-1 homolog B
PxSE4.3	31,764–31,620	LOC105393953	3′ UTR	General function prediction only	Ras-related protein Rab-24	ras-related protein Rab-24-like
PxSE4.4	787,511–787,311	LOC105382052	3′ UTR	RNA processing and modification	WW domain-containing protein ZK1098.1	transcription elongation regulator 1-like isoform X1
PxSE4.5	160,917–160,809	LOC105388357	CDS	ND	Serine/threonine kinase SAD-1	PAS domain-containing serine/threonine-protein kinase
PxSE4.6	1,160,410–1,160,304	LOC105398290	3′ UTR	General function prediction only	Ras-related protein Rab-24	ras-related protein Rab-24-like
PxSE4.7	819,669–819,771	LOC105385258	CDS	ND	Probable 4-coumarate-CoA ligase 2	probable 4-coumarate-CoA ligase 3
PxSE4.8	217,956–218,125	LOC105383715	CDS	ND	Integrin alpha-PS3 light chain (Precursor)	integrin alpha-PS5-like
PxSE5.1	306,942–307,066	LOC105398401	5′ UTR	ND	ND	CLK4-associating serine/arginine rich protein-like
PxSE5.2	101,368–101,480	LOC105393324	CDS	Coenzyme transport and metabolism	Molybdopterin synthase catalytic subunit	molybdopterin synthase catalytic subunit-like
PxSE5.3	993,048–992,940	LOC105383883	CDS	ND	Salivary glue protein Sgs-3 (Precursor)	uncharacterized protein
PxSE5.4	338,901–338,769	LOC105387600	3′ UTR	ND	Alpha-(1,3)-fucosyltransferase C	alpha-(1,3)-fucosyltransferase C-like
PxSE5.5	499,158–499,032	LOC105388075	3′ UTR	ND	Cuticle collagen 1 (Precursor)	breast cancer metastasis-suppressor 1-like protein isoform X1

*ND* not determined

Further analysis revealed the insertion of multiple copies of SINE families into introns of the same gene. As many as 60 elements inserted into introns of LOC105382892 gene, including 18, 14, 10, 11 and 7 copies of *PxSE1*, *PxSE2*, *PxSE3*, *PxSE4* and *PxSE5*, respectively (Additional file 10: Figure S9). A total of 95 genes were found to be inserted with at least ten copies of SINE elements (Additional file 10: Figure S9D). Thus, the *P. xylostella* SINE families contribute to structural variation in introns, which might influence the regulation of gene expression.

### Evolution and horizontal transposon transfer (HTT) of SINEs

**Fig. 6:**
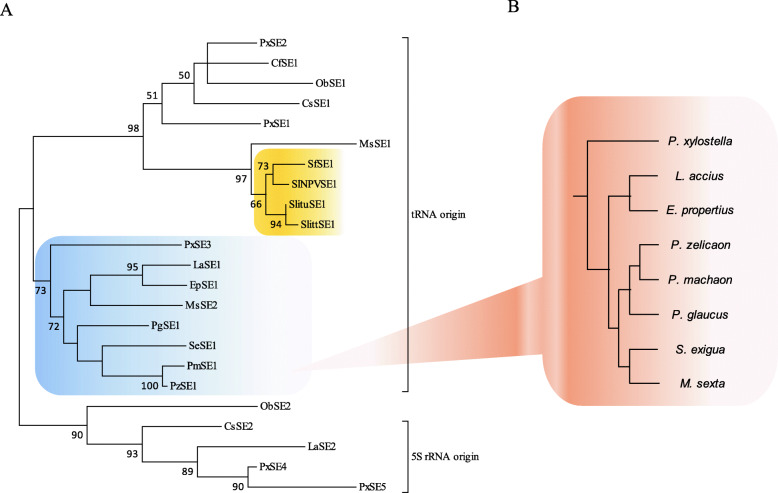
The evolutionary tree of 23 novel SINEs in this study (**a**) and the taxonomy tree of lepidopteran insects harboring *PxSE3*-like SINEs (**b**)

**Fig. 7:**
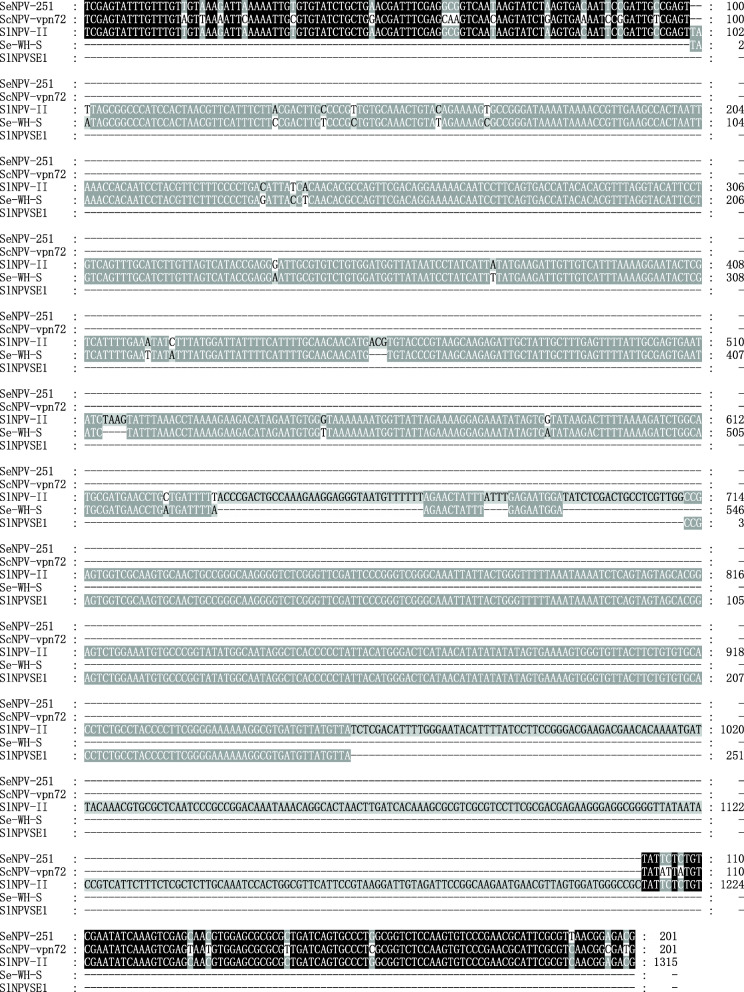
The evidence of HTT from Lepidoptera to baculovirus. Multiple sequence alignment of SlNPVSE1 and its flanking sequences and the orthologous sequences. Se-WH-S is a host sequence from *S. exigua* genome (WNNL01000005.1:248783–248238), SlNPV-II is baculovirus sequence from *S. litura* nucleopolyhedrovirus II (Accession number: EU780426.1:29774–31088) containing SINE copy, SeNPV-251 and ScNPV-vpn72 are orthologous sequecnes of SlNPV-II from *S. eridania* nucleopolyhedrovirus isolate 251 (Accession number: MH320559.1:31479–31679) and *S. cosmioides* nucleopolyhedrovirus isolate VPN72 (Accession number: MK419955.1:32601–32796), respectively

The phylogenetic tree of the 23 SINE consensus sequences showed that the SINEs with the same internal *Pol III* promoter were clustered together, except *ObSE2* SINE (Fig. 6a). Due to the high identity of *PxSE1* and *PxSE2* at 5′-ends, the clustering of related SINEs in different family, such as *PxSE1*, *PxSE2*, *CfSE1*, *ObSE1* and *CsSE1*, is not surprising. The comparison of phylogenetic tree of *PxSE3* family and the taxonomy tree of related host species [36, 37] (Fig. 6) suggests some degree of vertical transmission of *PxSE3* family in lepidopteran insects. Interestingly, *SlNPVSE1* and *SfSE1* in *Spodoptera frugiperda*, *SlittSE1* in *Spodoptera littoralis* and *SlituSE1* in *S. litura*, were clustered together (Table 1 and Fig. 6a). The orthologous outer flanking sequence of *SlNPVSE1* were identified in *Spodoptera eridania* nucleopolyhedrovirus isolate 251 and *Spodoptera cosmioides* nucleopolyhedrovirus isolate VPN72, suggesting that SlNPVSE1 inserted into the genome of nucleopolyhedrovirus by HTT. In addition, the inter 5′-flanking sequence (about 800 bp) was found to share 95% identity to the sequence (WNNL01000005.1: 248783–248238) of *Spodoptera exigua* genome (Additional file 11: Figure S10 and Fig. 7), putatively resulted from unknown horizontal gene transfer.

## Discussion

### The structure of three tRNA-derived and two 5S rRNA-derived SINE families

Up to now, more than 234 SINEs have been isolated from the genomes of human, mammals, reptiles, fishes, mollusks, fungus, green plants, and insects [2]. Based on current data, the tRNA-derived SINEs (~ 84%) were found widely in eukaryotic genomes [2]. Apart from the 5′ terminal head, SINEs also consist of typical body and variable repeated tail. In this study, we have identified three tRNA-derived SINE families, *PxSE1*, *PxSE2* and *PxSE3*. The 45 bp region in body region of *PxSE1* and *PxSE2* also showed high identity (93.3%) except the highly identical heads. Similarly, two 5S rRNA-derived SINEs, *PxSE4* and *PxSE5*, also shared 98.7% identity in 159 bp region of their bodies. Previous studies have found that the conserved bodies of SINE mainly include the V-domain, CORE-domain, Deu-domain, Nin-domain, Ceph-domain, Inv-domain, Pln-domain, Snail-domain, and Meta-domain [38–43]. However, the body regions identified in *PxSEs* are different from these known domains. A hypothesis has proposed that nonautonomous LINEs that have only 5′ and 3′ regions of original LINEs can be a source of enigmatic middle body of SINEs [1]. Hence, highly identical conserved central domains among different SINEs in the same species suggests that the conserved central domain may originated from the same LINE family and has been under strong selective constraint, which is important for reverse transcription. In addition, despite the high identity between *ObSE2* and 5S rRNA-derived *PxSE4*, *ObSE2* is a tRNA-derived SINE.

### Partner LINE

SINEs can be composed of 5′ and 3′ regions of nonautonomous LINEs, and their 3′ tails will also exchange with other LINEs under the pressure of natural selection to facilitate rapid amplification [1]. The tail homologous to LINE is important for SINE, which allows the integration of new copies of SINE into the new genomic locations using the LINE RT [44]. LINE RT can specifically recognize the 3′ homologous SINE tails, indicated that SINE can be mobilized by the retrotransposition machinery of a partner LINE [45]. Here, nine novel LINE copies in *P. xylostella,* and seven LINEs in each of the lepidopteran insects were identified with 3′-end similar to that of *PxSE5* and *SfSE1*, suggesting that the LINE identified in this study is an ancient retrotransposon and might widely exist in Lepidoptera insects. However, the 5′ regions among *SfSE1*, *PxSE5* and *PxLINE1* shared a large divergence, indicating these SINEs exploded after the exchange of 3′-tails. Moreover, the distinct 3′-end in other *PxSEs* suggested that these SINEs might be mobilized by other LINEs that were not identified yet.

### Relative age and distribution of SINEs in Lepidoptera insect

The copy numbers of SINEs varies among different families and species. In *P. xylostella*, the copy numbers of SINEs of tRNA origin is relatively higher than that of 5S rRNA origin. In particular, the copy numbers of *PxSE5* is only 1952. Previously, it was speculated that the type 1 promoter in 5S rRNAs is more dependent on upstream signals than the type 2 promoter in tRNAs, resulting in the Pol III promoter in a retroposed 5S rRNA copy presumably remains silent or is expressed at a low level [5]. In different species, the copy numbers of the same origin SINE is different. The copy numbers of *MsSE1* and *MsSE2* in *M. sexta* and *SfSE1* in *S. frugiperda* were 7513, 16,157 and 11,117, respectively, whereas only 4521 copies of *ObSE1* and 863 copies of *ObSE2* were found in *O. brumata.* The genome sizes of *M. sexta* and *S. frugiperda* are around 400 Mb, while *O. brumata* has larger genome size of 618 Mb. Hence, SINE copy number may not correlate with genome size. Some factors of 3′-tail, such as poly(A) tail or short direct repeats length, sequence conservation and distance to the transcriptional terminator, may affect the retroposition efficiency of the SINE families [46, 47]. In this study, the varied 3′-tail of these SINEs in different species may have affected their distribution in the genome. However, their relationship with the number of copies cannot be determined at this time.

Based on the divergence of the copies from the consensus sequence, the relative age distribution of identified SINEs was analyzed. Scattered age profiles were found in most SINEs among all species or within the genus, suggesting that the activity and accumulation of these SINEs are dynamic processes that can vary considerably between host lineages and SINE lineages. Especially, the highly identity and concentrated *PxSE1* showed that it most likely is a relatively young retransposon in the genome of *P. xylostella* and was generated by recent explosive amplification. The scattered distribution of *PxSE5* copies also suggests that it is older than other SINEs.

### SINEs contribute to DBM genome evolution

The ability of TEs to replicate and move in the genome affects the genomic structure, gene expression, and the divergence and evolution of host species [48–51]. The genome size of DBM is 343.575 Mb, of which the intronic region occupies of 35.23% (121.039 Mb) [52]. The integration pattern analysis revealed that the numbers of *PxSINEs* inserted into introns accounted for 44–51%, only 2–8 copies were inserted into exons, indicating that *PxSINEs* prefer to insert or accumulate in introns of genic regions. However, the proportions of different SINEs located within introns of *Solanaceae* range from 15 to 54% [53] and 96% of SINEs inside genes were located inside introns in *Zoysia japonica* and maize [54], suggesting that the distribution characteristics of SINEs varied in different species. Introns have long been an exemplar of regulated splicing, which affects and enhances almost every step of mRNA metabolism by the act of their removal [55]. In mice, a recent insertion of MT-C retrotransposon into DICER intron truncated its first 6 exons, providing an alternative promoter and a novel first exon. This change resulted in acquizition of oocyte-specific expression and is essential for fertility [56]. We speculate that the insertions of *PxSEs* into introns may provide signals for alternative splicing and polyadenylation, which may be a reflection of the host response to an ever-changing environment.

Importantly, we also noticed that only 25 copies of SINEs inserted into the genic exonsof DBM, of which 13 copies were found in 3′ UTR. In eukaryotic cells, some proteins (such as PUF protein) can bind to regulatory elements in the 3′ UTR of mRNAs and control mRNA stability, translation and localization [57]. The genes with the insertion of SINEs into exons are mainly annotated in terms of metabolism, cell division, signal transduction and transportation, and it remains to be elucidated whether some of the SINE insertions have an influence on gene expression.

### HTT of SINEs

Increasing evidence showed that HTT is a common phenomenon. So far, no less than 5689 HTT events have been recorded [58]. However, only a few HTT events of SINE have been detected, including the *SmaI-cor* SINE between coregonid and common ancestor of salmonid (Hamada et al. 1997), *Sauria* SINE between reptiles and mammals [59], *HaSE2* SINE between *Aphis gossypii* and Lepidoptera insects [33]. The long-term vertical inheritance property inherent in SINE and its dependence on active partner LINEs to move in new hosts may be the reason why HTT events rarely occur [47, 60], as was confirmed by the partial congruence between the phylogenetic trees of *PxSE3* families and host species in this study. Interestingly, *SlNPVSE1*, a SINE copy inserted into the baculovirus, shared more than 90% identity to the consensus sequence of *SfSE1*, *SlittSE1* and *SlituSE1* (Additional file 4: Figure S4B). In addition, the absence of target site duplication as well as upstream host sequence in SlNPV-II, suggested that non-homologous end-joining of double-strand breaks might be the mechanism of HTT. SlNPV can successfully infect *S. litura* and *S. exigua* [61]. *S. exigua* multicapsid nucleopolyhedrovirus (SeMNPV) DNA can also replicate in five non-permissive cell lines including SF21AEII, CLS-79, SpLi-221, hi-5 and BmN4 [62], indicating a wider host range of NPV. Thus, our finding suggests the occurrence of HTT of *PxSE1* between baculovirus and Lepidoptera insects. This is not surprising, because population genomics supported baculoviruses as vectors of horizontal transfer of insect transposons [63]. Similarly, the HTT of *Helitron* transposon *Hel-2* and *Tc1*-like transposon *TCp3.2* between insects and associated baculoviruses has been detected [64, 65]. Recent studies have revealed that the occurrence of HTT generally exhibits species ecological relationships, such as host-parasite [66, 67] and predator-prey [68, 69]. Additionaly, proviruses have been reported as vectors for HTT of *Sauria* SINE from reptiles to mammals [59]. Hence, it is necessary to further explore the HTT events of *PxSE1*-like elements mediated by baculoviruses.

## Conclusions

In this study, we identified three tRNA-derived SINEs and two 5S RNA-derived SINEs in the genome of *P. xylostella*, among which *PxSE1* is a relatively young retrotransposon and was generated by recent explosive amplification. Homology searches revealed scattered distribution of these elements in other Lepidopteran insects with variable copy numbers. The preference of PxSINEs to insert or accumulate in introns of genic regions indicated that *P. xylostella* SINE families contribute to structural variation in introns. The identification of *PxSE1*-like elements in the baculovirus and related lepidopteran host insects provides evidence of horizontal transfer facilitated by host-parasite interactions. These data may have implications for understanding the evolution and HT mechanisms of SINEs.

## Methods

### Data resources

The 235 publicly available insect databases of WGS assemblies including 33 Lepidoptera insects, EST, nucleotide (Nr/Nt), and TSA from National Center for Biotechnology Information (NCBI) (last accessed November 30, 2018) were used in this study (Additional file 12: Table S2). *P. xylostella* WGS was downloaded from NCBI [52]. As corresponding gene annotation file, the GFF files GCF_000330985.1 were used.

### Database search strategy

To identify SINE candidates, database searches were performed and composed of four steps. Firstly, the known SINE sequences, including tRNA-derived *HaSE1* from *Helicoverpa armigera* [33] and *BmSE* from *B mori* [28], 5S rRNA-derived HaSE3 from *H. armigera* [33], were used as queries for local blastn in the DBM genome. The sequences of high homology (at least 70% identity over at least 50 bp length to query) as well as 500 bp upstream and downstream flanking regions were extracted using TBtools [70] and analyzed for conserved structural motifs of SINEs such as internal RNA Pol III promoter and TSDs. The consensus sequences of *PxSE1* and *PxSE4* were determined by multiple sequence alignments. Secondly, the consensus sequences of *PxSE1* and *PxSE4* were searched against DBM genome by local BLASTN to identify other potential homologous sequences, and two other tRNA-derived *PxSE2* and *PxSE3* and a 5S rRNA-related *PxSE5* were identified. Thirdly, the 50-bp fragment at 3′-end of SINE families was used as query to search potential partner LINEs, and the LINE, *PxLINE1*, related to *PxSE5* was identified. Finally, insect genome databases as well as EST, Nr/Nt and TSA databases from NCBI were searched using consensus sequences of these five SINE families as queries to detect SINEs in species other than DBM*.*

### Copy number estimation

To estimate copy number and average divergence of SINEs, respective consensus sequences were used to search against related databases (Additional file 12: Table S2). All contiguous sequences with at least 80% identity at the nucleotide level to the consensus over 100 bp were used to estimate copy number in all species [71, 72]. Given the high sequence identity of 5′-ends in several copies of different SINE families in DBM, all those undistinguishable copies were ruled out. For example, *PxSE1* and *PxSE2* shared high identity of 120 bp sequence at 5′-ends, thus all copies aligned only with part or all of this 120 bp region in the consensus sequence were excluded for copy number analysis. Further, all fragments sharing at least 80% identity over at least 80% of the length of the consensus sequence were aligned and used for calculation of average divergence to consensus sequence with Kimura-2 parameter model [73]. The identity value of single copy to consensus sequence was rounded to an integer for the relative age distribution analysis [53].

### Gene association and genomic show cases

The association of DBM SINEs with annotated genes were investigated using custom Perl script from MapGene2Chrom (http://mg2c.iask.in/mg2c_v1.0) [74]. The integration of SINEs into genic regions including introns, coding and untranslated regions as well as the distances of intergenic copies to the closest neighboring gene were determined as described previously [53]. The number of SINEs within each region was counted and the results were graphically represented using MapGene2Chrom.

### Sequence analysis and phylogeny

SINE’s tRNA-like structure was checked with tRNAscan-SE [75], using mixed model and cove score cut off value = 0.01 as default. Multiple SINE copies were aligned by MUSCLE [76], and the alignments were visualized with GENEDOC (www.psc.edu/biomed/genedoc). The phylogeny of full consensus sequences of SINE families was built by MEGA 7.0 using Maximum Likehood with K2 + G model [77]. The reliability of the trees was tested using 1000 bootstrap replications [71].

## Supplementary Information

**Additional file 1: Figure S1.** Characteristic of *PxSE1* in *P. xylostella*. (A) the sequence of *PxSE1*. The pink nucleotides are TSD sequence, gray background present A box and B box structure, green background is 3′tail sequence. (B) the homology search of *PxSE1* in Repbase database.

**Additional file 2: Figure S2.** The consensus sequence of tRNA and 5S rRNA related SINE transposons in insect genomes. Nucleotides in red font are 3′ tail sequences.

**Additional file 3: Figure S3.** Multiple sequence alignment the consensus sequence of *PxSE1* (A), *PxSE2* (B), *PxSE3* (C), *PxSE4* (D), *PxSE5* (E) and their empty site sequences. The nucleotides of TSD are indicated with the red words. The nucleotides of 3′ tail sequence are indicated with the gray background.

**Additional file 4: Figure S4.** Multiple sequence alignment and evolutionary divergence estimation between the consensus sequences of SINEs. The number of base differences per site from between sequences are shown. All ambiguous positions were removed for each sequence pair. Evolutionary analyses were conducted in MEGA7.0.

**Additional file 5: Figure S5.** The 8 copies with high identity to *PxLINE1.1* in *P. xylostell*a (A) and the multiple sequence alignment of one copy (AHIO01028576.1: 13049_14357) and *Mariner-8_BM* (B).

**Additional file 6: Figure S6.** Alignment of potential LINE transposons in 8 lepidopteran insects genome. *PxLINE1* in *P. xylostella* (NW011952036.1: 552486–555,713), *TaLINE1* in *Tuta absoluta* (SNMR 01038797.1: 8533–11852), *McLINE1* in *Melitaea cinxia* (APLT01012314.1: 14517–16103), *CcLINE1* in *Conopomorpha cramerella* (SJJU01072145.1: 61771–65266), *GmLINE1* in *Galleria mellonella* (NHTH01000021.1: 4230671–4228043), *ArLINE1* in *Adela reaumurella* (WYDE01048472.1: 2507–535), *AhLINE1* in *Adoxophyes honmai* (BHDV01006067.1: 48096–49728), *DpLINE1* in *Dendrolimus punctatus* (JAABVI010000027.1: 8196917–8193378).

**Additional file 7: Figure S7.** The origin analysis of *ObSE2*. (A) the alignment of 75-bp fragment at 5′-end of *ObSE2* and 72-bp tRNA-related region of *D. melanogaster*. (B) the schematic representation of structure of *ObSE2*.

**Additional file 8: Table S1.** The copies of PxSE1 in the genome of *P. xylostella*.

**Additional file 9: Figure S8.** Examples for the relative age distribution of SINE families in other species based on the identity to the species-specific consensus. The abscissa showed that the identity between each consensus sequence and the copies. The ordinate showed that the copy numbers of sequence with the same identity.

**Additional file 10: Figure S9.** The typical integration pattern of SINEs within genome of *P. xylostella*. **(**A) (B) and (C) are schematic diagrams of several copies inserted into the introns of LOC105382892, LOC105381513 and LOC105383359, respectively. (D) Statistics number of different SINE families inserted into the same gene.

**Additional file 11: Figure S10.** Paralogous empty sites of *PxSE1* (A) in *P. xylostella* and *SfSE1* (B) in *S. frugiperda.* The nucleotides of TSD are indicated with the red background. The nucleotides of 3′ tail sequence are indicated with the gray background.

**Additional file 12: Table S2.** The databases of NCBI used for Blast searches, including 8 WGS databases, 3 EST databases and 2 TSA databases as well as the Nr/nt database.

## Data Availability

The original sequences used to construct the consensus sequences of all SINEs are uploaded to the NCBI database (http://www.ncbi.nlm.nih.gov/bioproject/665855). The complete genome of *Plutella xylostella* is available at the NCBI RefSeq assembly database under the accession number GCF_000330985.1 and the other lepidopteran genome databases including the accesion numbers are available in Additional file 12: Table S2.

## Abbreviations

SINEs: Short interspersed nuclear elements
LINEs: Long interspersed nuclear elements
TEs: Transposable elements
RT: Reverse transcriptase
DBM: Diamondback moth
TSDs: Target site duplications
UTR: Untranslated regions
WGS: Whole genome shotgun
EST: Expressed sequence tags
CDS: Coding regions
HTT: Horizontal transposon transfer
TSA: Transcriptome shotgun assembly
Nr/Nt: Nucleotide
NCBI: National Center for Biotechnology Information

## References

[1] Kojima KK (2018). LINEs contribute to the origins of middle bodies of SINEs besides 3′ tails. Genome Biol Evol..

[2] Vassetzky NS, Kramerov DA (2013). SINEBase: a database and tool for SINE analysis. Nucleic Acids Res.

[3] Kramerov DA, Vassetzky NS (2005). Short retroposons in eukaryotic genomes. Int Rev Cytol.

[4] Ohshima K, Okada N (2005). SINEs and LINEs: symbionts of eukaryotic genomes with a common tail. Cytogenet Genome Res.

[5] Kapitonov VV, Jurka J (2003). A novel class of SINE elements derived from 5S rRNA. Mol Biol Evol.

[6] Gogolevsky KP, Vassetzky NS, Kramerov DA (2009). 5S rRNA-derived and tRNA-derived SINEs in fruit bats. Genomics..

[7] Kojima KK (2015). A new class of SINEs with snRNA gene-derived heads. Genome Biol Evol..

[8] Longo MS, Brown JD, Zhang C, O’Neill MJ, O’Neill RJ (2015). Identification of a recently active mammalian SINE derived from ribosomal RNA. Genome Biol Evol..

[9] Suh A, Witt CC, Menger J, Sadanandan KR, Podsiadlowski L, Gerth M, Weigert A, McGuire JA, Mudge J, Edwards SV (2016). Ancient horizontal transfers of retrotransposons between birds and ancestors of human pathogenic nematodes. Nat Commun.

[10] Schramm L, Hernandez N (2002). Recruitment of RNA polymerase III to its target promoters. Genes Dev.

[11] Consortium IHGS (2001). Initial sequencing and analysis of the human genome. Nature..

[12] Kogler A, Schmidt T, Wenke T (2017). Evolutionary modes of emergence of short interspersed nuclear element (SINE) families in grasses. Plant J.

[13] Schwichtenberg K, Wenke T, Zakrzewski F, Seibt KM, Minoche AE, Dohm JC, Weisshaar B, Himmelbauer H, Schmidt T (2016). Diversification, evolution and methylation of short interspersed nuclear element families in sugar beet and related Amaranthaceae species. Plant J.

[14] Nishihara H, Okada N. Retroposons: genetic footprints on the evolutionary paths of life. In: Murphy WJ editor. Methods in molecular biology: phylogenomics. Totowa: Humana Press Inc; 2008. pp. 201–25. 10.1007/978-1-59745-581-7_13 18629669

[15] Lisch D (2013). How important are transposons for plant evolution?. Nat Rev Genet.

[16] Trizzino M, Park Y, Holsbach-Beltrame M, Aracena K, Mika K, Caliskan M, Perry GH, Lynch VJ, Brown CD (2017). Transposable elements are the primary source of novelty in primate gene regulation. Genome Res.

[17] Lev-Maor G, Ram O, Kim E, Sela N, Goren A, Levanon EY, Ast G (2008). Intronic *Alus* influence alternative splicing. PLoS Genet.

[18] Lee JY, Ji Z, Tian B (2008). Phylogenetic analysis of mRNA polyadenylation sites reveals a role of transposable elements in evolution of the 3′-end of genes. Nucleic Acids Res.

[19] Loke JC, Stahlberg EA, Strenski DG, Haas BJ, Wood PC, Li QQ (2005). Compilation of mRNA polyadenylation signals in *Arabidopsis* revealed a new signal element and potential secondary structures. Plant Physiol.

[20] Sorek R, Ast G, Graur D (2002). *Alu*-containing exons are alternatively spliced. Genome Res.

[21] Volff JN (2006). Turning junk into gold: domestication of transposable elements and the creation of new genes in eukaryotes. Bioessays..

[22] Durruthy-Durruthy J, Sebastiano V, Wossidlo M, Cepeda D, Cui J, Grow EJ, Davila J, Mall M, Wong WH, Wysocka J (2016). The primate-specific noncoding RNA HPAT5 regulates pluripotency during human preimplantation development and nuclear reprogramming. Nat Genet.

[23] Deininger P (2011). *Alu* elements: know the SINEs. Genome Biol.

[24] Luchetti A, Lomiento M, Mantovani B (2019). Riding the Wave. The SINE-specific V highly-conserved domain spread into mammalian genomes exploiting the replication burst of the MER6 DNA transposon. Int J Mol Sci.

[25] Ben-David S, Yaakov B, Kashkush K (2013). Genome-wide analysis of short interspersed nuclear elements SINES revealed high sequence conservation, gene association and retrotranspositional activity in wheat. Plant J.

[26] Bao W, Kojima KK, Kohany O (2015). Repbase update, a database of repetitive elements in eukaryotic genomes. Mob DNA.

[27] Adams DS, Eickbush TH, Herrera RJ, Lizardi PM (1986). A highly reiterated family of transcribed oligo (a)-terminated, interspersed DNA elements in the genome of *Bombyx mori*. J Mol Biol.

[28] Xu J, Liu T, Li D, Zhang Z, Xia Q, Zhou Z (2010). BmSE, a SINE family with 3′ ends of (ATTT) repeats in domesticated silkworm (*Bombyx mori*). J Genet Genomics.

[29] Tu Z (1999). Genomic and evolutionary analysis of Feilai, a diverse family of highly reiterated SINEs in the yellow fever mosquito, *Aedes aegypti*. Mol Biol Evol.

[30] Feschotte C, Fourrier N, Desmons I, Mouches C (2001). Birth of a retroposon: the twin SINE family from the vector mosquito *Culex pipiens* may have originated from a dimeric tRNA precursor. Mol Biol Evol.

[31] Kapitonov V, Jurka J (2007). SINE3-1_TC, a family of SINE3 retrotransposons from the red flour beetle genome. Repbase Rep.

[32] Santolamazza F, Mancini E, Simard F, Qi Y, Tu Z, della Torre A (2008). Insertion polymorphisms of SINE200 retrotransposons within speciation islands of *Anopheles gambiae* molecular forms. Malaria J.

[33] Wang J, Wang A, Han Z, Zhang Z, Li F, Li X (2012). Characterization of three novel SINE families with unusual features in *Helicoverpa armigera*. PLoS One.

[34] Kojima KK, Jurka J (2015). SINEs from the Asian swallowtail genome. Repbase Rep.

[35] Jurka J (2010). DNA transposons from *Bombyx mori*. Repbase Rep.

[36] Kawahara AY, Plotkin D, Espeland M, Meusemann K, Toussaint EFA, Donath A, Gimnich F, Frandsen PB, Zwick A, dos Reis M, Barber JR, Peters RS, Liu S, Zhou X, Mayer C, Podsiadlowski L, Storer C, Yack JE, Misof B, Breinholt JW (2019). Phylogenomics reveals the evolutionary timing and pattern of butterflies and moths. Proc Natl Acad Sci U S A.

[37] Zakharov EV, Caterino MS, Sperling FAH (2004). Molecular phylogeny, historical biogeography, and divergence time estimates for swallowtail butterflies of the genus Papilio (Lepidoptera: Papilionidae). Syst Biol.

[38] Nishihara H, Plazzi F, Passamonti M, Okada N (2016). MetaSINEs: broad distribution of a novel SINE superfamily in animals. Genome Biol Evol.

[39] Akasaki T, Nikaido M, Nishihara H, Tsuchiya K, Segawa S, Okada N (2010). Characterization of a novel SINE superfamily from invertebrates: "Ceph-SINEs" from the genomes of squids and cuttlefish. Gene..

[40] Gilbert N, Labuda D (1999). CORE-SINEs: eukaryotic short interspersed retroposing elements with common sequence motifs. Proc Natl Acad Sci U S A.

[41] Ogiwara I, Miya M, Ohshima K, Okada N (2002). V-SINEs: a new superfamily of vertebrate SINEs that are widespread in vertebrate genomes and retain a strongly conserved segment within each repetitive unit. Genome Res.

[42] Nishihara H, Smit AF, Okada N (2006). Functional noncoding sequences derived from SINEs in the mammalian genome. Genome Res.

[43] Matetovici I, Sajgo S, Ianc B, Ochis C, Bulzu P, Popescu O, Damert A (2016). Mobile element evolution playing jigsaw-SINEs in gastropod and bivalve mollusks. Genome Biol Evol..

[44] Kramerov D, Vassetzky N (2011). Origin and evolution of SINEs in eukaryotic genomes. Heredity..

[45] Kajikawa M, Okada N (2002). LINEs mobilize SINEs in the eel through a shared 3′ sequence. Cell..

[46] Roy-Engel AM, Salem A-H, Oyeniran OO, Deininger L, Hedges DJ, Kilroy GE, Batzer MA, Deininger PL (2002). Active *Alu* element “A-tails”: size does matter. Genome Res.

[47] Comeaux MS, Roy-Engel AM, Hedges DJ, Deininger PL (2009). Diverse cis factors controlling *Alu* retrotransposition: what causes *Alu* elements to die?. Genome Res.

[48] Liu D, Yang J, Tang W, Zhang X, Royster CM, Zhang M (2020). SINE retrotransposon variation drives ecotypic disparity in natural populations of *Coilia nasus*. Mob DNA.

[49] Ray DA, Grimshaw JR, Halsey MK, Korstian JM, Osmanski AB, Sullivan KAM, Wolf KA, Reddy H, Foley N, Stevens RD, Knisbacher BA, Levy O, Counterman B, Edelman NB, Mallet J (2019). Simultaneous TE analysis of 19 heliconiine butterflies yields novel insights into rapid te-based genome diversification and multiple SINE births and deaths. Genome Biol Evol..

[50] Trizzino M, Kapusta A, Brown CD (2018). Transposable elements generate regulatory novelty in a tissue-specific fashion. BMC Genomics.

[51] Huang K, Li CF, Wu J, Wei JH, Zou Y, Han MJ, Zhou ZY (2016). Enhancer activity of *Helitron* in *sericin-1* gene promoter from *Bombyx mori*. Insect Sci.

[52] You M, Yue Z, He W, Yang X, Yang G, Xie M, Zhan D, Baxter SW, Vasseur L, Gurr GM, Douglas CJ, Bai J, Wang P, Cui K, Huang S, Li X, Zhou Q, Wu Z, Chen Q, Liu C, Wang B, Li X, Xu X, Lu C, Hu M, Davey JW, Smith SM, Chen M, Xia X, Tang W, Ke F, Zheng D, Hu Y, Song F, You Y, Ma X, Peng L, Zheng Y, Liang Y, Chen Y, Yu L, Zhang Y, Liu Y, Li G, Fang L, Li J, Zhou X, Luo Y, Gou C, Wang J, Wang J, Yang H, Wang J (2013). A heterozygous moth genome provides insights into herbivory and detoxification. Nat Genet.

[53] Seibt KM, Wenke T, Muders K, Truberg B, Schmidt T (2016). Short interspersed nuclear elements (SINEs) are abundant in Solanaceae and have a family-specific impact on gene structure and genome organization. Plant J.

[54] Mao H, Wang H (2017). Distribution, diversity, and long-term retention of grass short interspersed nuclear elements (SINEs). Genome Biol Evol..

[55] Le Hir H, Nott A, Moore MJ (2003). How introns influence and enhance eukaryotic gene expression. Trends Biochem Sci.

[56] Flemr M, Malik R, Franke V, Nejepinska J, Sedlacek R, Vlahovicek K, Svoboda P (2013). A retrotransposon-driven dicer isoform directs endogenous small interfering RNA production in mouse oocytes. Cell..

[57] Wickens M, Bernstein DS, Kimble J, Parker R (2002). A PUF family portrait: 3′ UTR regulation as a way of life. Trends Genet.

[58] Dotto BR, Carvalho EL, da Silva AF, Dezordi FZ, Pinto PM. Campos TdL, Rezende AM, Wallau GdL. HTT-DB: new features and updates. Database. 2018;2018. 10.1093/database/bax102. 10.1093/database/bax102 PMC7206651 29315358

[59] Piskurek O, Okada N (2007). Poxviruses as possible vectors for horizontal transfer of retroposons from reptiles to mammals. Proc Natl Acad Sci U S A.

[60] Luchetti A, Mantovani B (2016). Rare horizontal transmission does not hide long-term inheritance of SINE highly conserved domains in the metazoan evolution. Curr Zool.

[61] Takatsuka J, Okuno S, Ishii T, Nakai M, Kunimi Y (2007). Host range of two multiple nucleopolyhedroviruses isolated from *Spodoptera litura*. Biol Control.

[62] Yanase T, Yasunaga C, Kawarabata T (1998). Replication of *Spodoptera exigua* nucleopolyhedrovirus in permissive and non-permissive lepidopteran cell lines. Acta Virol.

[63] Gilbert C, Chateigner A, Ernenwein L, Barbe V, Bézier A, Herniou EA, Cordaux R (2014). Population genomics supports baculoviruses as vectors of horizontal transfer of insect transposons. Nat Commun.

[64] Jehle JA, Nickel A, Vlak JM, Backhaus H (1998). Horizontal escape of the novel Tc1-like lepidopteran transposon TCp3.2 into *Cydia pomonella* granulovirus. J Mol Evol.

[65] Coates BS (2015). Horizontal transfer of a non-autonomous *Helitron* among insect and viral genomes. BMC Genomics.

[66] Guo X, Gao J, Li F, Wang J (2014). Evidence of horizontal transfer of non-autonomous *Lep1* Helitrons facilitated by host-parasite interactions. Sci Rep.

[67] Han G, Zhang N, Xu J, Jiang H, Ji C, Zhang Z, Song Q, Stanley D, Fang J, Wang J (2019). Characterization of a novel *Helitron* family in insect genomes: insights into classification, evolution and horizontal transfer. Mob DNA.

[68] Tang Z, Zhang HH, Huang K, Zhang XG, Han MJ, Zhang Z (2015). Repeated horizontal transfers of four DNA transposons in invertebrates and bats. Mob DNA.

[69] Župunski V, Gubenšek F, Kordis D (2001). Evolutionary dynamics and evolutionary history in the RTE clade of non-LTR retrotransposons. Mol Biol Evol.

[70] Chen C, Chen H, Zhang Y, Thomas H, Frank MH, He Y, Xia R (2020). TBtools: an integrative toolkit developed for interactive analyses of big biological data. Mol Plant.

[71] Gilbert C, Schaack S, Pace JK, Brindley PJ, Feschotte C (2010). A role for host-parasite interactions in the horizontal transfer of transposons across phyla. Nature..

[72] Zhang HH, Xu HE, Shen YH, Han MJ, Zhang Z (2013). The origin and evolution of six miniature inverted-repeat transposable elements in *Bombyx mori* and *Rhodnius prolixus*. Genome Bio Evol.

[73] Lerat E, Rizzon C, Biémont C (2003). Sequence divergence within transposable element families in the *Drosophila melanogaster* genome. Genome Res.

[74] Chao JT, Kong YZ, Wang Q, Sun YH, Gong DP, Lv J, Liu GS (2015). MapGene2Chrom, a tool to draw gene physical map based on Perl and SVG languages. Hereditas..

[75] Lowe TM, Eddy SR (1997). tRNAscan-SE. A program for improved detection of transfer RNA genes in genomic sequence. Nucleic Acids Res.

[76] Edgar RC (2004). MUSCLE: multiple sequence alignment with high accuracy and high throughput. Nucleic Acids Res.

[77] Kumar S, Stecher G, Tamura K (2016). MEGA7: molecular evolutionary genetics analysis version 7.0 for bigger datasets. Mol Biol Evol.

