# Supplementary material for: Diversity of short interspersed nuclear elements (SINEs) in lepidopteran insects and evidence of horizontal SINE transfer between baculovirus and lepidopteran hosts
Source: BMC Genomics. 2021 Mar 31;22:226. doi: 10.1186/s12864-021-07543-z (PMC8010984; doi:10.1186/s12864-021-07543-z)
Supplement: Supplementary file 1 — Additional file 1: Figure S1. Characteristic of PxSE1 in P. xylostella. (A) the sequence of PxSE1. The pink nucleotides are TSD sequence, gray background present A box and B box structure, green background is 3′tail sequence. (B) the homology search of PxSE1 in Repbase database. [file 12864_2021_7543_MOESM1_ESM.docx]

A

>PxSE1

GTCAAACGTCACCGAAACGGGACGACCGAATGGCGTGGACGACCGAATGGCGTAGTGGTTAGTGACCTGACTACTCAGCCGTAGGTCCCGGGTTCGATTCCCGGCTGGGGCAGATATTTGTTTAAACACAGATATTTGTTCTCGGGTCTTGGATGTGCCCGTAAAATGGCAATAGGGCCGCCCCTATTACATTGGGACTAAAATAACACTCTGGCGAAAAGTCCCTGCAGCAATGCACCTCTGCCTACCCCGCAAGGGAGTACATTAGTACAAGGCGTGAGTGCGTGTGTGTGTGTGTGTGTCAAACGTC

B


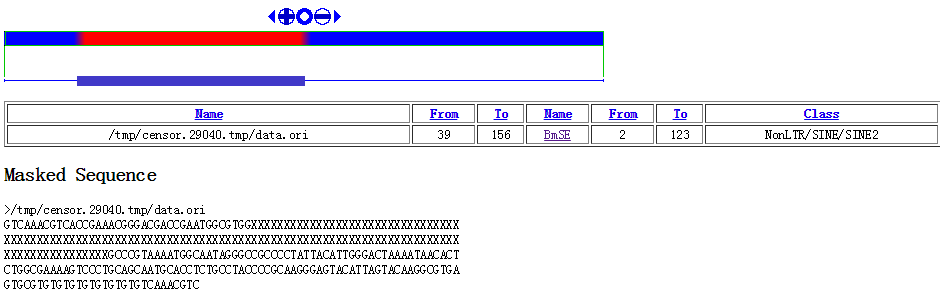

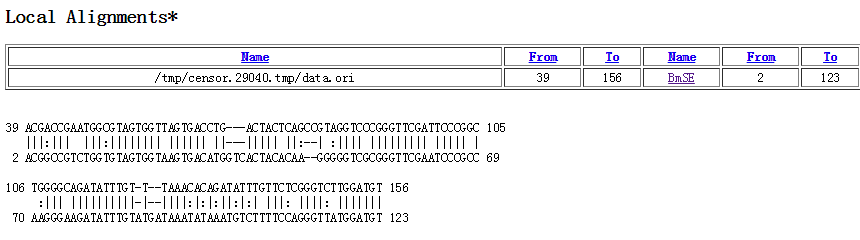


**Figure S1**
