# Supplementary material for: Diversity of short interspersed nuclear elements (SINEs) in lepidopteran insects and evidence of horizontal SINE transfer between baculovirus and lepidopteran hosts
Source: BMC Genomics. 2021 Mar 31;22:226. doi: 10.1186/s12864-021-07543-z (PMC8010984; doi:10.1186/s12864-021-07543-z)
Supplement: Supplementary file 2 — Additional file 2: Figure S2. The consensus sequence of tRNA and 5S rRNA related SINE transposons in insect genomes. Nucleotides in red font are 3′ tail sequences. [file 12864_2021_7543_MOESM2_ESM.docx]

>CfSE1 [organism= Choristoneura fumiferana] SINE transposon consensus sequence

AGACGACCGGATGGCCTAGTGGTTAGAGAACCTGACTACGAAGCTTGAGGTCCCGGGTTCGATTCCCGTGTCGGGGCAGATATTTGTATGAATAATACGAATGTTTGTTCTCGGGTCTTGGGTGTTTAATATGTATTTAAGTATGTATTATATATATAATTATATTTATCCGTTGCTTAGTACCATACACAAGCTTTGCTTATTTGGGACTAGGTCAATTGGTGTGAATTGTCCCGTGATATTTATTTATTT

>CsSE1 [organism= Chilo suppressalis] SINE transposon consensus sequence

TTTGACGACCTCCGTGGCCGAGTGGTTTGTACGCCGGGTTTCATGGTGTCGCCGACTCGAGAGGTCCCGGGTTCGAATCCCGGTGGGGGCAGATGTTTGTGTGATGAATACGAGCATTTGTACTCCAGTCATGGATGTTTAATATGTATTTCTATATAAATATACTTATATATGTACAGTATATGTATTCTATCCGTTACCCTAGTATCCCATAACACAAGCCTTACAGTGCTTACTTTGGGGCTAGGCTAATTGGTGTGAGTGTTGGAAATATTTATTTATTTATT

>CsSE2 [organism= Chilo suppressalis] SINE transposon consensus sequence

GAGGAGCTCGATGGCGCAAGTGGTTAGCGCGTTCGGCTGCGATCGTTGAAGTTAAGCAACTTTCGCAGTGGTCGGTCATTGGATGGGTGACCAAAAATGTACTATCTCGATCTCCTCCGTGCTTCGGAAGGCACTTTAAGCCGTTGGTCCCGGCTGCATCTGCAGTCGTTAGCACCCACCAATCCGCACTGGGCCCGCGTGGTGGGTCATGGCCCAATCTCCCTATTCCATCCATAGGGAAGGCCTGTGCCCCAGCAGTGGGGACATTAATAGGCTGATGATGATGATGATGAT

>EpSE1 [organism= Erynnis propertius] SINE transposon consensus sequence

TGATAGCCCAGTGGCTAAGAATTCGGCCTCTTACTCGAGGGGTCCCGGGTTCGAATCCCAGTGGCACGCACCAATGACTTCCTTAAAAGTTATGTGCGCTTTATAAAATTAAATATCACTTGCTGTAACGGTGAAGGAAAACATCGTGAGGAAACCTGCATGCCTGAGAGTTCTCCATAATGTTCTCAAAGGCGTGTGAAGTCCACCAACCCGCATTAGGCCAGCGTGGTGGACTAAGGCCTACATCCTTCTCATTGTGAGAGGAGACCCGACCCTGTAGTGGGTCATTAATGGGTTACA

>LaSE1 [organism= Lerema accius] SINE transposon consensus sequence

AAGCAGTGATAGCCTAGTGGTTTGAACGTTGGTCTCTCGAACGGGAGATCCTGGGTTCAAACCCCACGGCACGCACCTCTAATTTAAAAAGTTATGTGCGTATTAGATACTAATTATCACTTGCTACGGTGAAGGAAAACATCGTGAGGAAACCTGCACACCTGAGAGTTCTCCATAAAGTTCTCAAAGGTGTGTGAAGTCTGCCAATCCGCACTAGGCCAGCGTGGTGGACTATGGCCTGTACCCCTCACACTGTGAGAGGAGACCCGAGCCCAGCAGTGGGACAGTTATGGGTTGCAATGATGATGATGATGATGAT

>LaSE2 [organism= Lerema accius] SINE transposon consensus sequence

GAAGCGCTCATAGCCCGGCGGCTTGCGTCGATCCTAAGCGATCGACAAAGTTAAGCAACTGCGGGCGCGGTCGGTAAGTGGATGGGTGACCGCTAGTGGCAAAAATATCTTGAGCGTTTCCGTGCTTCGGAGGGCACGTTAAGCCGTCGGTCCCGGTTGTTACCATTAGGTGACAATCGTTAGCCATGTCGGAGGCCTTTGGGTGGCATAAAAATTTCCGACACTTGGGTTGGCCACTAACCCCTCCGCCAATCCGCACTGGAGCAGCGTGGTGGAGTATGCTCTAGGCCTCCTCAGTTATGAGGAGAGACCCGTGCTCAGTAGTGGGCCGTTAATGGGTTGAGGCCA

>MsSE1 [organism= Manduca sexta] SINE transposon consensus sequence

GACTGCCTCGGTGGCGTAGTTGTACTGCATGCGCGGTACGGCAGCGCTCTGAGGTCCTGGGTTCGAATCCCGGGTCGGGCAAAGTGATATTTGGGTTTTTCTGCTCAGTATCAGCCCGGAGTCTGGAATTTGTGCCCGATATGGCGATAGGCTCGCCCCCTATCACATCATGGGACGGAACACACTTGGCGAAAAGTGGGTGCCATGGTTGCGCCTCTGCATACCCCTTCGGGGATAAAATGCGTGATGTTGTGTGTGTGTGTGTGT

>MsSE2 [organism= Manduca sexta] SINE transposon consensus sequence

AAGCGGCGATAGCCTAGTTGGGTGTGGAACGGACTGCCGAGACGAATGTCCGCAGGTTCAAATCCCAAGGGCACACACCTCTGACTTTTCTAAAAAATCATGTGTGTATTCTTTGTGAATTTATCGTTCGCTTTAACGGTGAAGGAAAACATCGTGAGGAAACCTGCACATCTGAGAAGTTCTCTATAGGAATTTCGAAGGTGTGTGAAGTCTACCAATCCGCACTAGGCCAGCGTGGTGGACTAAGGCCTAATCCCTCTCAGTAGTAGAGGAGGCCCGTGCTCAGCAGTGGGCAAGTATATAATACAGGGCTGATATTATTATTATTATTAT

>ObSE1 [organism= Operophtera brumata] SINE transposon consensus sequence

TTGACGACCTCTGTGGCGCGGCGGGCGAAGCTTGGCCTGCGACGCAGGAGGTTGCGGGTTCGATTCCCGCCTCGGAACAAATATTTGTGCGATCGCAGATATTTGTTCAGGGTGTTCTGGGTATTTTCTATGTATAATAAAAAATATCTATCTATGTAATATATTGTATTAAGTATATCAGTTATCTAGTACCCATAACACAAGCTCTGCTTAGCTTGGGGCTAGATAATGTTGTGTGAACTGTCCAGCATTATTTATTTATTTATTTATTTATT

>ObSE2 [organism= Operophtera brumata] SINE transposon consensus sequence

AGCGATGGTAGCCTAACGGTTCAATAGTGCAACTCAGAATCCAACGATACCGGGTTCGATCCTAGGCTCCGCACCAATGACTTTTCTAAGTTATGTGCGGATTTCCATCGCTCTGTGCTTCGGAGGGCACGTTAAGCTGTCGGTCCCGGCTGTTATTGATTAATAGCAGTCGATAAGCCATGTCAGAGGCCCTCGGGCGGCTTGACAAACTCTGACACCAGAGGCCAGCACTACCTCATACGAAACGAAACGAAA

>PmSE1 [organism= Papilio machaon] SINE transposon consensus sequence

AGAGCGTCGGTGGCTCAGGGGTTAAGCACTTGACTTGCAATCTGCAGGTCCTGGGTTCGAATCCCGCCATGTACCAATGTGTTTTTCGATTTTCGATTTACATATGTACATTTATCCGACGTTCTTACGGTGAAGGAAAACATCGTGATGCAACCTGCACATATCTGAGAAGAAATTCAATGATATGTGTGAAGTCAACCAACCCGCACTTGGCCAGCGTGGTTGACTATGGCCTAGTCACCCCTAACTTGGGGTAGGCTCCGAGCCCCTCGGTGGGGACGTATAGTGAGCTGATGATGATGATGATGAT

>PgSE1 [organism= Papilio glaucus] SINE transposon consensus sequence

GGAGCAGTGGTGGCTCAGTGGTTTAGGCCCCGACTTACGTACTGTAGGTCGCGGGTTCGAACCCAGGCAGGCGCTTTACTTTGTGTTAATTTATTTCATCACCACTGCTCCAAAAACGGTGAAGGAAAACATCGTGAGGAAACCGGCATGTCTTAGAACCAATAAATTCGAAGACATGTGACATCCACCAACCCGCACTGGGCCAGCGTGGTGGATTACGGCCCCTTCCCTCTCTCGGAGAGGAGGCCTGTGCCCCTGCAGTGGGGAACATTCTAGAAAAGGCTGATTTATTATTATTATTAT

>PxSE1 [organism= Plutella xylostella] SINE transposon consensus sequence

GACGACCGAATGGCGTAGTGGTTAGTGACCCTGACTACTGAGCCGATGGTCCCGGGTTCGATTCCCGGCTGGGGCAGATATTTGTTTAAACACAGATATTTGTTCTCGGGTCTTGGATGTGCCCGTAAAATGGCAATAGGCCCGCCCCCTATTACATTGGGACTAACATAACACTCTGGCGAAAAGTGGGTGCAGCAATGCACCTCTGCCTACCCCGCAAGGGAGTACATTAGTACAAGGCGTGAGTGCGTGTGTGTGTGTGT

>PxSE2 [organism= Plutella xylostella] SINE transposon consensus sequence

GACGACCGAATGGCGTAGTGGTTAGTGGCCCTGACTGCTATGCCGAAGGTCCCGGGTTCGATTCCCGGCTGGGGCAGATATTTGTTTAAAGACAGATATTTGTACTCGGGTCTTGGGTGTTGATATTTATATTTAGTATCTATCTATCTATGTATTTGTGTAGATATATCAGCTGTCCGACACCCATAACACAGGTTCTGCCTAGCTTGGGGTCGGATGGCCGTGTGTGAGATGTCCCCACATATTTATTTATTTATTTATTT

>PxSE3 [organism= Plutella xylostella] SINE transposon consensus sequence

GAGCGGTGGTAGCTCAGTCGGGTAAGCGCCCGCTTCTCACGCCAGAGATGCGGGTTCGAATCCCGGCGCTGACATGTACCAATGAGTTCTTTTCTGAATTTAAGTACAATGTATACCATCGCTCTTACGGTGAAGGAAAACATCGTGAGGAAACCTGCATATCTAGATTTAGCACATCTAGATATGTGAACCCACCAACCCGCAGTGGACCAGCGTGGTGGGAAAATGGTCCAAGCTTAGGAAGGCAGTTTAGACCTTGGGGATATGCACAAAGGTTCCATTCGAGAGAGCCAGGTGCAGGTACTGTTACCCCCACAGAGAATAGAATAGAATAGAATA

>PxSE4 [organism= Plutella xylostella] SINE transposon consensus sequence

GAAGCGTCCGTAGTCGAGCGGGCCTCAGTGATCGTAACTGATCGCTGAGGTTAAGCAACAACTGACACGGTCAGCCATTGGATGGGTGACCAATTTCAAGTGGTGCTTTTCTGGACGCTTCCGTGCTTCGGACGGCACGTTAAGCCGTGGGTCCCGGTTGCTGCTTCGGCAGCAGTCGTTAAGCCTAGTCAGAGGCCTTCGGGCGGCTTGAAAACATCTGACAGTCGGGTTGCCCACTTACCCGACAACTCTCTCAGCACAAGCTTGCTTGTGTTGGGGTCCACCAACCCGCACTTGGCCAGCGTGGTGGACTAGGCCTAAACCCTTCCTTCATTGGAAGGAGACCCGTGCCCCAGCAGTGGGGACGTAATGGGTCGTGATGATGATGA

>PxSE5 [organism= Plutella xylostella] SINE transposon consensus sequence

GAAGCGTCCGTAGTCGAGCGGGCTTCAGTGATCGTAACTGATCACTGAGGTTAAGCAACAACTGACACGGTCAGCCATTGGATGGGTGACCGATTTCAAGTGGTTCTTTTCTGGACGCTTCCGTGCTTCGGACGGCACGTTAAGCCGTGGGTCCCGGTTGCTGCTTCGGCAGCAGTCGTTAAGCCTAGTCAGAGGCCTTCGGGCGGCTTGAAAACATCTGACAGTCGGGTTGCCCACTTACCCGACAACTTGCATTGTACTCATTCAAAAACGGCGATACGGCTCGCGACCTATCACGTGAGTACAATGCACAGCGAAAAGCGGGTGACTCGCTTGCGAGTCACCTCTGACTACCCCTTCGGGGATTACAGTCGTGAGCATATGTATGT

>PzSE1 [organism= Papilio zelicaon] SINE transposon consensus sequence

GAGCGTCGGTGGCTCAGGGGTTAAGCACTTGACTTGCAATCTGCAGGTCCTGGGTTCGAATCCCGCCATGTACCAATGTGTTTTTCGATTTCGATTTACATATGTACATTTATCCGACGTTCTTACGGTGAAGGAAAACATCGTGATGCACCTGCACATATCTGAGAAGAAATTCAATGATATGTGTGAAGTCAACCCGCACTGGCCAGCGTGGTTGACTATGGCCTAGTCACCCCTAACTTGGGGTAGGCTCCGAGCCCCTCAGTGGGGACGTATAGTGAGCTGATGATGAT

>SeSE1 [organism= Spodoptera exigua] SINE transposon consensus sequence

GTCGTGGTGGCCCGGAGGTTAAGGCGCCCGCTTCTCATGCATGAGGGTGTGGGTTCGAAACCTGGCAAGTACCAATGTGACTTTTTCCGAGTTATATGTACTTTCTATGATTATTTAGACACCACTGACATACGGTGAAGGAAACATCGTGAGGAAACCTGGACTTATAATTTCTAATTATAAGTTTGAAATCGCCAACCGCTTGAGCAAGCGTGGTGATTAATGCTCAAACCTTCTCCGTGTGAGAAGAGGCCTTTGCTCAGCAGTGGGCACTTATAGGCTGATGATGAT

>SfSE1 [organism= Spodoptera frugiperda] SINE transposon consensus sequence

TGACTGCCTCGTTGGCCGAGTGGTTGCAAGTGCGACTGCCGGGCAAGGGGTCTCGGGTTCGATTCCCGGGTCGGGCGAAGTATTACTGGGCTTTTTTCGGTTTTTCGAAAATTTCTCAGTGGTAGCACGGAGTCTGGAAATGTGCCCGGTATATGGCAATAGGCTCACCACCTATTACATGGGACTTACAACATAAATTGTGAAAAGTGGGTGTACACAGTGGCATTACGTGCCATAATGTGCACCTCTGCCTACCCCTTCGGGGATTAAAGGCGTGACGATATGTATGTATGTATGT

>SlittSE1 [organism= Spodoptera littoralis] SINE transposon consensus sequence

ACTGCCTCGTTGGCCGAGTGGTCGCAAGTGCGACTGCCGGGCAAGGGGTCTCGGGTTCGATTCCCGGGTCGGGCAAAGTATTACTGGGTTTTTAAAAAAATCTCAGTAGTAGCACGGAGTCTGGAATTGTGCCCGGTATATGGCAATAGGCTCACCCCCTATTACATGGGACTCATAACATAAATAGTGAAAAGTGGGTGTTACATTGTACAGTGGCATAATGTGCACCTCTGCCTACCCCTTCGGGGATAAAAGGCGTGATGTTATGTT

>SlituSE1 [organism= Spodoptera litura] SINE transposon consensus sequence

CCGAGTGGTCGCAAGTGCGACTGCCGGGCAAGGGGTCTCGGGTTCGATTCCCGGGTCGGGCAAAGTATTACTGGGTTTTTAAAAAAATCTAGTAGTAGCACGGAGTCTGGAATTGTGCCCGGTATATGGCAATAGGCTCACCCCCTATTACATGGGACTCATAACATAAATAGTGAAAAGTGGGTGTTACATCGTACAGTGCTAATGTGCACCTCTGCCTACCCCTTCGGGGATAAAAGGCGTGATGTTATGTTATGTT

>SlNPVSE1 [organism= Spodoptera litura nucleopolyhedrovirus II] SINE transposon consensus sequence

CCGAGTGGTCGCAAGTGCAACTGCCGGGCAAGGGGTCTCGGGTTCGATTCCCGGGTCGGGCAAATTATTACTGGGTTTTTAAATAAAATCTCAGTAGTAGCACGGAGTCTGGAAATGTGCCCGGTATATGGCAATAGGCTCACCCCCTATTACATGGGACTCATAACATATATATATAGTGAAAAGTGGGTGTTACTTCTGTGTGCACCTCTGCCTACCCCTTCGGGGAAAAAAGGCGTGATGTTATGTTA

**Figure S2**
