# Supplementary material for: Diversity of short interspersed nuclear elements (SINEs) in lepidopteran insects and evidence of horizontal SINE transfer between baculovirus and lepidopteran hosts
Source: BMC Genomics. 2021 Mar 31;22:226. doi: 10.1186/s12864-021-07543-z (PMC8010984; doi:10.1186/s12864-021-07543-z)
Supplement: Supplementary file 3 — Additional file 3: Figure S3. Multiple sequence alignment the consensus sequence of PxSE1 (A), PxSE2 (B), PxSE3 (C), PxSE4 (D), PxSE5 (E) and their empty site sequences. The nucleotides of TSD are indicated with the red words. The nucleotides of 3′ tail sequence are indicated with the gray background. [file 12864_2021_7543_MOESM3_ESM.docx]

A

PxSE1 1 TAAAAACGAAATGTTTAATTTCACATTTATATCGTTCACAACATATAATTAATTACCTTACCTATTATAACATCTATATTTTCATAAAGTATAATATTAT
PxSE1_EMT 1 TAAAAACGAAATGTTTAATTTCACATTTATATCGTTCACAACATATAATTAATTACCTTACCTATTATAACATCTATATTTTCATAAAGTATAATATTAT

PxSE1 101 TAGACGTAGTATTAGACGACCGAATGGCGTAGTGGTTAGTGACCTGACTACTGAGCCGATGGTCCCGGGTTCGATTCCCGGCTGGGGCAGATATTTGTTT
PxSE1_EMT 101 ----------------------------------------------------------------------------------------------------

PxSE1 201 AAACACAGATATTTGTTCTCGGGTCTTGGATGTGCCCGTAAAATGGCAATAGGCCCGCCCCCTATTACATTGGGACTAACATAACACTCTGGCGAAAAGT
PxSE1_EMT 101 ----------------------------------------------------------------------------------------------------

PxSE1 301 GGGTGCAGCAATGCACCTCTGCCTACCCCGCAAGGGAGTACATTAGTACAAGGCGTGAGTGCGTGTGTGTGTGTGTGTGTGTATAATATTATACATGTTG
PxSE1_EMT 101 --------------------------------------------------------------------------------------------ACATGTTG

PxSE1 401 CCAGTGATGACCTACGGTGCCGAGGTCTTACGGCCTACATGGTCTTTAGGCACAGCAAGCTATGGAAAGGGCTATGCTCGGCGTATCTCTAC
PxSE1_EMT 109 CCAGTGATGACCTACGGTGCCGAGGTCTTACGGCCTACATGGTCTTTAGGCACAGCAAGCTATGGAAAGGGCTATGCTCGGCGTATCTCTAC

B

PxSE2 1 TTTTGATACGTTAATAAACAAAATACCATTTTAGAAAACATATGAAAGAATGGATTTTTAGTAATAACAAAATATTTATTAAGTAGTAATTACCACTCTG
PxSE2_EMP 1 TTTTGATACGTTAATAAACAAAATACCATTTTAGAAAACATATGAAAGAATGGATTTTTAGTAATAACAAAATATTTATTAAGTAGTTATTACCACTCTG

PxSE2 101 ACGGTCGAATGGCGTAGTGGTTAGTGGCCCTGACTGCTATGCCGAAGGTCCCGGGTTCGATTCCCGGCTGGGGCAGATATTTGTTTAAAGACAGATATTT
PxSE2_EMP 101 ----------------------------------------------------------------------------------------------------

PxSE2 201 GTACTCGGGTCTTGGGTGTTGATATTTATATTTAGTATCTATCTATCTATGTATTTGTGTAGATATATCAGCTGTCCGACACCCATAACACAGGTTCTGC
PxSE2_EMP 101 ----------------------------------------------------------------------------------------------------

PxSE2 301 CTAGCTTGGGGTCGGATGGCCGTGTGTGAGATGTCCCCACATATTTATTTATTTATTTATTTATTTATTTACTCTGTCACAATTTCAGCTAAAATGAAGC
PxSE2_EMP 101 ----------------------------------------------------------------------------TCACAATTTCAGCTAAAATGAAGC

PxSE2 401 TTGTTTTTGCTATAAATATTTTTTCGCTACTAATTACAGTAAAAAATTAACAGCATAGACGTTAACATCAATGACT
PxSE2_EMP 125 TTGTTTTTGCTATAAATATTTTTTCGCTACTAAATACAGTCAAAAATTAACAGCATAGACGTTAACATCAATGACT

C

PxSE3 1 CTACTTACTTCGCAATTTTTGGTCAGCTTTGGTTAATATCTCCTACATTACTTCGGTAGGTACTTACTAGTTAGTTTTATATAGTTTTTAGGCAGGTCTC
PxSE3_EMP 1 CTACTTACTTCGCAATTTTTGGTCAGCTTTGTTTAATATCTCCTACATTACTTCGGTAGGT----ACTAGTTAGTTTTATATAGTTTTTAGGCAGGTCTC

PxSE3 101 TCTCTCTAGCAGGCTCTCTAGAGCGGTGGTAGCTCAGTCGGGTAAGCGCCCGCTTCTCACGCCAGAGATGCGGGTTCGAATCCCGGCGCTGACATGTACC
PxSE3_EMP 97 ----------------------------------------------------------------------------------------------------

PxSE3 201 AATGAGTTCTTTTCTGAATTTAAGTACAATGTATACCATAGCTCTTACGGTGAAGGAAAACATCGTGAGGAAACCTGCATATCTAGATTTAGCACATCTA
PxSE3_EMP 97 ----------------------------------------------------------------------------------------------------

PxSE3 301 GATATGTGAACCCACCAACCCGCAGTGGACCAGCGTGGTGGGGAAATGGTCCAAGCTTAGGAAGGCAGTTTAGACCTTGGGGATATGCACAAAGGTTCCA
PxSE3_EMP 97 ----------------------------------------------------------------------------------------------------

PxSE3 401 TTCGAGAGAGCCAGGTGCAGGTACTGTTACCCCCACAGAGAATAGAATAGAATAGAATAGGCAGGTCTCAGGTCTCATATACATAGGGCATTATTTTAAA
PxSE3_EMP 97 ---------------------------------------------------------------------AGGTCTCATATACATAGGGCATTATTTTAAA

PxSE3 501 GCGGGCACTTTGAAGTAGCTCGACTTAGAGAGTGTCCATTAATTGGTTAAGCGATTACTTAGCCATTAT
PxSE3_EMP 128 GCGGGCACTTTGAAGTAGCTCGACTTAGAGAGAGTCCATTAATTGGTTAAGCGATTACTTACCCATTAT

D

PxSE4 1 TAAGCGTTAATTAAGAAGTGTATTCAATAAAAGGAATTAAGTACCTACCTGTTAATTCTGTAATATTATAAGAAAGAATTATCATTTATTACCATTACCA
PxSE4_EMP 1 TAAGCGTTAATTAAGAAGTGTATTCAATAAAAGGAATTAAGTACCTACCTGTTAATTCTGTAATATTATAAGAAAGAATTATCATTTATTACCATTACCA

PxSE4 101 GAAGCGTCCGTAGTCGAGCGGGCCTCAGTGATCGTAACTGATCGCTGAGGTTAAGCAACAACTGACACGGTCAGCCATTGGATGGGTGACCAATTTCAAG
PxSE4_EMP 101 ----------------------------------------------------------------------------------------------------


PxSE4 201 TGGTTCTTTTCTGGACGCTTCCGTGCTTCGGACGGCACGTTAAGCCGTGGGTCCCGGTTGCTGCTTCGGCAGCAGTCGTTAAGCCTAGTCAGAGGCCTTC
PxSE4_EMP 102 ----------------------------------------------------------------------------------------------------

PxSE4 301 GGGCGGCTTGAAAACATCTGACAGTCGGGTTGCCCACTTACCCGACAACTCTCTCAGCACAAGCTTGCTTGTGTTGGGGTCCACCAACCCGCACTTGGCC
PxSE4_EMP 102 ----------------------------------------------------------------------------------------------------

PxSE4 401 AGCGTGGTGGACTAGGCCTAAAACCCTTCCTTCATTGGAAGGAGACCCGTGCCCCAGCAGTGGGGACGTAATGGGTCGTGATGATGACCATTACCAGGCT
PxSE4_EMP 102 ------------------------------------------------------------------------------------------------GGCT

PxSE4 501 GCTAAACCTCATTAAATACCTGTGCTTTAGGTCTGTGTTGCGCCTGTATCGTATTCTTTTCTTTACAACTACCTATACTCCCAGGCGCTCCTATAC
PxSE4_EMP 105 GCTAAACCTCATTAAATACCTGTGCTTTAGGTCTGTGTTGCGCCTGTATCGTATTCTTTTCTTTACAACTACCTATACTCCCAGGCGCTCCTATAC

E

PxSE5 1 TTGAATCGTATTCCCATTCAGGTTTGATTGAATTGAATAAATGATGTAAAGGCCATTTATGATATGATCAAACGTTGATTGAAAAGCTTTTGATTTAGGC
PxSE5_EMP 1 TTGAATCGTATTCCCATTCAGGTTTGATTGAATTGAATAAATGATGTAAAGGCCATTTATGATATGATCAAACGTTGATTGAAAAGCTTTTGATTTAGGC

PxSE5 101 GTCCGTAGTCGAGCGGGCTTCAGTGATCGTAACTGATCACTGAGGTTAAGCAACAACTGACACGGTCAGCCATTGGATGGGTGACCGATTTCAAGTGGTT
PxSE5_EMP 101 ----------------------------------------------------------------------------------------------------

PxSE5 201 CTTTTCTGGACGCTTCCGTGCTTCGGACGGCACGTTAAGCCGTGGGTCCCGGTTGCTGCTTCGGCAGCAGTCGTTAAGCCTAGTTAGAGGCCTTCGGGCG
PxSE5_EMP 101 ----------------------------------------------------------------------------------------------------

PxSE5 301 GCTTGAAAACATCTGACAGTCGGGTTGCCCACTTACCCGACAACTTGCATTGTACTCATTCAAAAACGGCGATACGGCTCGCGACCTATCACGTGAGTAC
PxSE5_EMP 101 ----------------------------------------------------------------------------------------------------

PxSE5 401 AATGCACAGCGAAAAGCGGGTGACTCGATTGCGAGTCACCTCTGACTACCCCTTCGGGGATTACAGTCGTGAGCATATGTATGTATGTTGATTTAGGCTT
PxSE5_EMP 101 --------------------------------------------------------------------------------------------------TT

PxSE5 501 GTGTCAATGCGTGATATGTTTTTGCCGTTTTTGCCTGTATTTTGTT--GTTTATACGATTTAAAAAAGTTCCCTAGTCCTGTGATTTTGTCAAATTTT
PxSE5_EMP 103 GTGTCAATGCGTGATATGTTTTTGCCGTTTTTGCCTGTATTTTGTTATGTTTATACGATTTAAAAAAGTTCCCTAGTCCTGTGATTTTGTCAAATTTT

**Figure S3**
