# Supplementary material for: Diversity of short interspersed nuclear elements (SINEs) in lepidopteran insects and evidence of horizontal SINE transfer between baculovirus and lepidopteran hosts
Source: BMC Genomics. 2021 Mar 31;22:226. doi: 10.1186/s12864-021-07543-z (PMC8010984; doi:10.1186/s12864-021-07543-z)
Supplement: Supplementary file 4 — Additional file 4: Figure S4. Multiple sequence alignment and evolutionary divergence estimation between the consensus sequences of SINEs. The number of base differences per site from between sequences are shown. All ambiguous positions were removed for each sequence pair. Evolutionary analyses were conducted in MEGA7.0. [file 12864_2021_7543_MOESM4_ESM.docx]

A

PxSE1 : -GACGACCG-AATGGCGTAGTGGT-TAGTGACCCTG--ACTACTGAGC-CGATGGTCCCGGGTTCGATTCCCGGCTG-GGGCAGAT-------------------ATTTGTTTAAA-- : 90
SfSE1 : TGACTGCCTCGTTGGCCGAGTGGT--TGCAAGTGCG--ACTGCCGGGC-AAGGGGTCTCGGGTTCGATTCCCGGGTC-GGGCGAAGTATTACTGGGCTTTTTTCGGTTTTTCGAAAAT : 112
SlNPVSE1 : ---------------CCGAGTGGT--CGCAAGTGCA--ACTGCCGGGC-AAGGGGTCTCGGGTTCGATTCCCGGGTC-GGGCAAATTATTACTGG----------GTTTTTAAATAAA : 87
SlittSE1 : --ACTGCCTCGTTGGCCGAGTGGT--CGCAAGTGCG--ACTGCCGGGC-AAGGGGTCTCGGGTTCGATTCCCGGGTC-GGGCAAAGTATTACTGG----------GTTTTTAAAAAAA : 100
SlituSE1 : ---------------CCGAGTGGT--CGCAAGTGCG--ACTGCCGGGC-AAGGGGTCTCGGGTTCGATTCCCGGGTC-GGGCAAAGTATTACTGG----------GTTTTTAAAAAAA : 87
MsSE1 : -GACTGCCTCGGTGGCGTAGTTGTACTGCATGCGCGGTACGGCAGCGCTCTGAGGTCCTGGGTTCGAATCCCGGGTC-GGGCAAAGTGATATTTG---------GGTTTTTCTG---- : 103
CfSE1 : AGACGACCG-GATGGCCTAGTGGT-TAGAGAACCTG--ACTACGAAGC-TTGAGGTCCCGGGTTCGATTCCCGTGTCGGGGCAGAT-------------------ATTTGTATGAATA : 94

PxSE1 : -CACAGATATTTGTTCTCGGGTCTTGGA--TGTGCCCGTAAAATGGCAATAGGCCCGCCCCCTATTA---CATTGGGACTAACATAACACTC---TGGCGAAAAGTGGGTGCAGCAA- : 198
SfSE1 : TTCTCAGTGGTAGCACG-GAGTCTGGAA-ATGTGCCCGGTATATGGCAATAGGCTCACCACCTATTA---CA-TGGGACTTACAACATAAA----TTGTGAAAAGTGGGTG-TACACA : 219
SlNPVSE1 : ATCTCAGTAGTAGCACG-GAGTCTGGAA-ATGTGCCCGGTATATGGCAATAGGCTCACCCCCTATTA---CA-TGGGACTCATAACATATATATATAGTGAAAAGTGGGTGTTACTT- : 198
SlittSE1 : T-CTCAGTAGTAGCACG-GAGTCTGGAA-TTGTGCCCGGTATATGGCAATAGGCTCACCCCCTATTA---CA-TGGGACTCATAACATAAA----TAGTGAAAAGTGGGTGTTACAT- : 206
SlituSE1 : --TCTAGTAGTAGCACG-GAGTCTGGAA-TTGTGCCCGGTATATGGCAATAGGCTCACCCCCTATTA---CA-TGGGACTCATAACATAAA----TAGTGAAAAGTGGGTGTTACAT- : 192
MsSE1 : --CTCAGTATCAGCCCG-GAGTCTGGAATTTGTGCCCG--ATATGGCGATAGGCTCGCCCCCTATCACATCA-TGGGAC--GGAACACACT----TGGCGAAAAGTGGGTGCCATGG- : 208
CfSE1 : ATACGAATGTTTGTTCTCGGGTCTTGGGTGTTTA------ATATG-----------------TATTT------AAGTATGTATTATATATA----TAATTATATTTATCCGTTGCTT- : 178

PxSE1 : ---------------------TGCAC---CTCTGCCTACCCCGCAAGGGAGTACATTAGTACAAGGCGTGA--GTGC------GTGTGTGTGTGTGT- : 263
SfSE1 : GTGGCATTACGTGCCATAATGTGCAC---CTCTGCCTACCCCTTCGGGGA---------TTAAAGGCGTGACGATAT------GTATGTATGTATGT- : 298
SlNPVSE1 : ----------------CTGTGTGCAC---CTCTGCCTACCCCTTCGGGGA---------AAAAAGGCGTGATGTTAT------GT-----------TA : 251
SlittSE1 : ----TGTACAGTGGCATAATGTGCAC---CTCTGCCTACCCCTTCGGGGA---------TAAAAGGCGTGATGTTAT------------------GTT : 270
SlituSE1 : ------CGTACAGTGCTAATGTGCAC---CTCTGCCTACCCCTTCGGGGA---------TAAAAGGCGTGATGTTAT------GT-------TATGTT : 259
MsSE1 : --------------------TTGCGC---CTCTGCATACCCCTTCGGGGA---------TAAAATGCGTGATGTTGT------GTGTGTGTGTGTGT- : 267
CfSE1 : --------------AGTACCATACACAAGCTTTGCTTATTT-----GGGACTAGGT---CAATTGGTGTGA-ATTGTCCCGTGATATTTATTTATTT- : 252

B

|  | PxSE1 | SfSE1 | SlNPVSE1 | SlittSE1 | SlituSE1 | MsSE1 | CfSE1 |
| --- | --- | --- | --- | --- | --- | --- | --- |
| PxSE1 |  |  |  |  |  |  |  |
| SfSE1 | 0.301 |  |  |  |  |  |  |
| SlNPVSE1 | 0.304 | 0.098 |  |  |  |  |  |
| SlittSE1 | 0.292 | 0.078 | 0.049 |  |  |  |  |
| SlituSE1 | 0.279 | 0.105 | 0.057 | 0.043 |  |  |  |
| MsSE1 | 0.275 | 0.206 | 0.211 | 0.189 | 0.208 |  |  |
| CfSE1 | 0.329 | 0.409 | 0.383 | 0.39 | 0.381 | 0.436 |  |

C

PxSE2 : ---GACGACCGA-ATGGCGTAGTGGTTAGTGGCCCTGACTG-----------CTATGCCGAAGGTCCCGGGTTCGATTCCCGGCT-GGGGCAGATATTTGTTTAAAG---ACAGATATTTG : 102
ObSE1 : -TTGACGACCTCTGTGGCGCGGCGGGC-GAAGCTTGGCCTG-----------CGACGCAGGAGGTTGCGGGTTCGATTCCCGCCTCGGAACAAATATTTGTGCGATC---GCAGATATTTG : 105
CsSE1 : TTTGACGACCTCCGTGGCCGAGTGGTTTGTACGCCGGGTTTCATGGTGTCGCCGACTCGAGAGGTCCCGGGTTCGAATCCCGGTG-GGGGCAGATGTTTGTGTGATGAATACGAGCATTTG : 120

PxSE2 : TACTCGG-GTCTTGGGTG-TTGATATTTATATTTA-------GTATCTATCTATCTA-TGTATTTGTGTAGATATATCAGCTGTCC-GACA-CCCATAACACAGGTTC-----TGCCTAGC : 206
ObSE1 : TTCAGGGTGTTCTGGGTATTTTCTATGTATAATAA----AAAATATCTATCTATGTAATATATTGTATTAAGTATATCAGTTATCT-AGTA-CCCATAACACAAGCTC-----TGCTTAGC : 215
CsSE1 : TACTCCA-GTCATGGATGTTTAATATGTATTTCTATATAAATATACTTATATATGTACAGTATATGTAT---TCTATCCGTTACCCTAGTATCCCATAACACAAGCCTTACAGTGCTTACT : 237

PxSE2 : TTGGGGTCGGATGGCCGTGTGTGAGATGTCCCCACATATTTATTTATTTATTTATTT--- : 263
ObSE1 : TTGGGGCTAGATAATGTTGTGTGAACTGTCCAGCATTATTTATTTATTTATTTATTTATT : 275
CsSE1 : TTGGGGCTAGGCTAATTGGTGTGAG-TGT-TGGAAATATTTATTTATTTATT-------- : 287

D

|  | PxSE2 | ObSE1 | CsSE1 |
| --- | --- | --- | --- |
| PxSE2 |  |  |  |
| ObSE1 | 0.282 |  |  |
| CsSE1 | 0.3 | 0.312 |  |

E

PxSE3 : -GAGCGGTGGTAGCTCAGTCGGGTAAGCGCCCG-CTTCTCACGCCAGAGAT-GCGGGTTCGAATCCCGGCGCTGACATGTACCAATGAGTTCTTTTCTGAATTTAAGTACAATGTAT--- : 114
MsSE2 : -AAGCGGCGATAGCCTAGTTGGGTGTGGAACGGACTGCCGAGACGAATGTCCGCAGGTTCAAATCCCAA---GGGCACACACCTCTGACTT-TTCTAAAAAATCATGTGTGTATTCTTTG : 115
PgSE1 : GGAGCAGTGGTGGCTCAG-TGGTTTAGGCCCCGACTTAC-GTACTGTAGGTCGCGGGTTCGAACCC------AGGCAGGCGC----------TTT----ACTTTGTGT-TAATTTAT--- : 94
PmSE1 : AGAGCGTCGGTGGCTCAG-GGGTTAAGCACTTGACTTGC-AATCTGCAGGTCCTGGGTTCGAATCC------CGCCATGTACCAATGTG---TTTTTCGATTTT-----CGATTTACATA : 104
LaSE1 : -AAGCAGTGATAGCCTAG-TGGTTTGAACGTTGGTCTCTCGAACGGGAGATCCTGGGTTCAAACCCCA----CGGCACGCACCTCTA-----ATTTAAAAAGTTATGTGCGTATTAGATA : 109
PzSE1 : -GAGCGTCGGTGGCTCAG-GGGTTAAGCACTTGACTTGC-AATCTGCAGGTCCTGGGTTCGAATCC------CGCCATGTACCAATGTG---TTTTTCGATTT------CGATTTACATA : 102
EpSE1 : -------TGATAGCCCAG-TGGCTAAGAATTCGGCCTCTTACTCGAGGGGTCCCGGGTTCGAATCCCAG---TGGCACGCACCAATGACT--TCCTTAAAAGTTATGTGCGCTTTATAAA : 107
SeSE1 : ---GTCGTGGTGGCCCGG-AGGTTAAGGCGCCCGCTTCTCATGCATGAGGGTGTGGGTTCGAAACC------TGGCAAGTACCAATGTGAC-TTTTTCCGAGTTATATGTACTTTCTATG : 109

PxSE3 : -----------ACCATCGCTCT---TACGGTGAAGGAAAACATCGTGAGGAAACCTG--CATATCTAGA--------TTTAGCACATCT----AGATATGTGAA-CCCACCAACCCGCAG : 205
MsSE2 : TGAATTTAT-CGTT--CGCTTT---AACGGTGAAGGAAAACATCGTGAGGAAACCTG--CACATCTGAGAAGTTCTCTATAGGAATTTCGA--AGGTGTGTGAAGTCTACCAATCCGCAC : 225
PgSE1 : ----TTCAT-CACCACTGCTCCAAAAACGGTGAAGGAAAACATCGTGAGGAAACCGG--CATGTCTTAGAAC-------CAATAAATTCGA--AGACATGTGACATCCACCAACCCGCAC : 198
PmSE1 : TGTACATTT-ATCCGACGTTCT---TACGGTGAAGGAAAACATCGTGATGCAACCTGCACATATCTGAG-----------AAGAAATTCAATGATATGTGTGAAGTCAACCAACCCGCAC : 209
LaSE1 : C-TAATTAT-CACT-----TGC---TACGGTGAAGGAAAACATCGTGAGGAAACCTG--CACACCTGAG-AGTTCTCCATAAAGTTCTCAA--AGGTGTGTGAAGTCTGCCAATCCGCAC : 214
PzSE1 : TGTACATTT-ATCCGACGTTCT---TACGGTGAAGGAAAACATCGTGATGC-ACCTGCACATATCTGAG-----------AAGAAATTCAATGATATGTGTGAAGTC----AACCCGCAC : 202
EpSE1 : ATTAAATAT-CACT--TGCTGT---AACGGTGAAGGAAAACATCGTGAGGAAACCTG--CATGCCTGAG-AGTTCTCCATAATGTTCTCAA--AGGCGTGTGAAGTCCACCAACCCGCAT : 216
SeSE1 : ATTATTTAGACACCACTGACAT----ACGGTGAAGG-AAACATCGTGAGGAAACCTGGACTTAT------------------AATTTCTAATTATAAGTTTGAAATC-GCCAA-CCGC-T : 203

PxSE3 : TGGACCAGCGTGGTGGGAAAATGGTCCA----------AGCTTAGGAAGGCAGTTTAGACCTTGGGGATATGCACAAAGGTTCCATTCGAGAGAGCCAGGTGCAGGTACTGTTACCCCCA : 315
MsSE2 : TAGGCCAGCGTGGT-GGACTAAGGCCTA-ATCCCTCTCAGTAGTAGAGGAGGCCCGTG-CTCAG----------CAGTGGGCAAGTATATAATACAGGGCTGATATTATTATTATTATTA : 332
PgSE1 : TGGGCCAGCGTGGT-GGATTACGGCCCC-TTCCCTCTC--TCGGAGAGGAGGCCTGTGCCCCTG----------CAGTGGGGAA-CATTCTAGAAAAGGCTGAT-TTATTATTATTATTA : 302
PmSE1 : TTGGCCAGCGTGGT-TGACTATGGCCTA-GTCACCCCTAACTTGGGGTAGGCTCCGAGCCCCT-----------CGGTGGGGACGT-----ATAGTGAGCTGATG--ATGATGATGATGA : 309
LaSE1 : TAGGCCAGCGTGGT-GGACTATGGCCTGTACCCCTCACACTGTGAGAGGAGACCCGAG-CCCAG----------CAGTGGGACAGT-------TATGGGTTGCAATGATGATGATGATGA : 315
PzSE1 : T-GGCCAGCGTGGT-TGACTATGGCCTA-GTCACCCCTAACTTGGGGTAGGCTCCGAGCCCCT-----------CAGTGGGGACGT-----ATAGTGAGCTGATG-----------ATGA : 292
EpSE1 : TAGGCCAGCGTGGT-GGACTAAGGCCTACATCCTTCTCATTGTGAGAGGAGACCCGA--CCCTG----------TAGTGGGTCATT-------AATGGGTTACA---------------- : 300
SeSE1 : TGAGCAAGCGTGGT--GATTAATGCTCA-AACCTTCTCCGTGTGAGAAGAGGCCTTTG-CTCAG----------CAGTGGGCACTT--------ATAGGCTGATG-----------ATGA : 290

PxSE3 : CAGAGAATAGAATAGAATAGAATA : 339
MsSE2 : T----------------------- : 333
PgSE1 : T----------------------- : 303
PmSE1 : T----------------------- : 310
LaSE1 : TGAT-------------------- : 319
PzSE1 : T----------------------- : 293
EpSE1 : ------------------------ : -
SeSE1 : T----------------------- : 291

F

|  | PxSE3 | MsSE2 | PgSE1 | PmSE1 | LaSE1 | PzSE1 | EpSE1 | SeSE1 |
| --- | --- | --- | --- | --- | --- | --- | --- | --- |
| PxSE3 |  |  |  |  |  |  |  |  |
| MsSE2 | 0.413 |  |  |  |  |  |  |  |
| PgSE1 | 0.344 | 0.274 |  |  |  |  |  |  |
| PmSE1 | 0.32 | 0.351 | 0.306 |  |  |  |  |  |
| LaSE1 | 0.421 | 0.257 | 0.312 | 0.327 |  |  |  |  |
| PzSE1 | 0.305 | 0.346 | 0.3 | 0.003 | 0.331 |  |  |  |
| EpSE1 | 0.328 | 0.271 | 0.287 | 0.324 | 0.192 | 0.321 |  |  |
| SeSE1 | 0.351 | 0.359 | 0.281 | 0.37 | 0.344 | 0.367 | 0.313 |  |

G

PxSE4 : GAAGCGTCCG-TAGTCGAGCGGGCCTCAGTGATCGTAACTGATCGCTGAGGTTAAGCAACAACTGACACGGTC--AGCCATTGGATGGGTGACCAATTTCAAGTGGTG---CTTTTCTGGA : 115
LaSE2 : GAAGCGCTCA-TAGCCCGGCGGCTTGCGTCGATCCTAAGCGATCGACAAAGTTAAGCAACTGCGGGCGCGGTC--GGTAAGTGGATGGGTGACCGCT----AGTGGCAAAAATATCTTGAG : 114
CsSE2 : GAGGAGCTCGATGGCGCAAGTGGTTAGCGCGTTCGGCTGCGATCGTTGAAGTTAAGCAACTTTCGCAGTGGTC--GGTCATTGGATGGGTGACCA-----AAAATGTA---CTATCTCGAT : 111
ObSE2 : --AGCGATGG-TAGCCTAACG---------GTTCAATAGTGCAACTCAGAATCCAACGATACCGGGTTCGATCCTAGGCTCCGCACCAATGACTTTTCT-AAGTTATGTGCGGATTTCCAT : 108

PxSE4 : CGCTTCCGTGCTTCGGACGGCACGTTAAGCCGTGGGTCCCGGTTGCTGCT--TCGGCAGCAGTCGTTAAGCCTAGTCAGAGGCCTTCGGGCGGCTTGAAAACATCTGACAGTCGGGTTGCC : 234
LaSE2 : CGTTTCCGTGCTTCGGAGGGCACGTTAAGCCGTCGGTCCCGGTTGTTACCATTAGGTGACAATCGTT-AGCCATGTCGGAGGCCTTTGGGTGGCATAAAAATTTCCGACACTTGGGTTGGC : 234
CsSE2 : CTCCTCCGTGCTTCGGAAGGCACTTTAAGCCGTTGGTCCCGGCTGCATCT--------GCAGTCGTT-AGC-------------------------------------------------- : 173
ObSE2 : CGC-TCTGTGCTTCGGAGGGCACGTTAAGCTGTCGGTCCCGGCTGTTATTGATTAATAGCAGTCGATAAGCCATGTCAGAGGCCCTCGGGCGGCTTGACAAACTCTGACA----------- : 217

PxSE4 : CACTTACCCGACAACTCTCTCAGCACAAGCTTGCTTGTGTTGGGGTCCACCAACCCGCACTTGGCCAGCGTGGTGGACTAGGCCTAAACCCTT------CCTTCATTGGAAGGAGACCCGT : 349
LaSE2 : CACTAACCC-----------------------------------CTCCGCCAATCCGCACTGGAGCAGCGTGGTGGAGTATGCTCTAGGCCTCCT----CAGTTATGAGGA-GAGACCCGT : 315
CsSE2 : --------------------------------------------ACCCACCAATCCGCACTGGGCCCGCGTGGTGGGTCATGGCCCAATCTCCCTATTCCATCCATAGGGA--AGGCCTGT : 248
ObSE2 : ----------------------------------------------CCAGAGGCCAGCACT--------------------------------------ACCTCATACGAA---------- : 244

PxSE4 : GCCCCAGCAGTGGGGACG-TAATGGGTCGTGATGATGATGA------- : 389
LaSE2 : G-CTCAGTAGT-GGGCCGTTAATGGGT--TGAGGCCA----------- : 348
CsSE2 : GCCCCAGCAGTGGGGACATTAATAGGC--TGATGATGATGATGATGAT : 294
ObSE2 : ---------------------ACGAAA--CGAAA-------------- : 255

H

|  | PxSE4 | LaSE2 | CsSE2 | ObSE2 |
| --- | --- | --- | --- | --- |
| PxSE4 |  |  |  |  |
| LaSE2 | 0.268 |  |  |  |
| CsSE2 | 0.28 | 0.305 |  |  |
| ObSE2 | 0.363 | 0.406 | 0.439 |  |

I

PxSE5 : GAAGCGTCCGTAGTCGAGCGGGCTTCAGTGATCGTAACTGATCACTGAGGTTAAGCAACAACTGACACGGTCAGCCATTGGATGGGTGAC : 90
SfSE1 : ----------------------------------------------------------TGACTGCCTCG--------TTGGCCGAGT--- : 21

PxSE5 : CGATTTCAAGTGGTTCTTTTCTGGACGCTTCCGTGCTTCGGACGGCACGTTAAGCCGTGGGTCCCGGTTGCTGCTTCGGCAGCAGTCGTT : 180
SfSE1 : -GGTTGCAAGTG---------------------------CGACTGCCGGGCAAG----GGGTCTCGGGTTCGATTCCCGGGTCGGGCG-- : 77

PxSE5 : AAGCCTAGTCAGAGGCCTTCGGGCGGCTTGAAAACATCTGACAGTCGGGTTGCCCACTTACCCGACAACTTGCATTGTACTCATTCAAAA : 270
SfSE1 : AAGTATTACTGGGCTTTTTTCGGTTTTTCGAAAATTTCT--CAGTGG----------TAGCACGGAGTCTGGAAATGTGCCCGGT---AT : 152

PxSE5 : ACGGCGATACGGCTCGCGACCTATCACGTGAG---TACAATGCACA--GCGAAAAGCGGGTG-----ACTCGC-TTGCGAGT-------- : 341
SfSE1 : ATGGCAATA-GGCTCACCACCTATTACATGGGACTTACAACATAAATTGTGAAAAGTGGGTGTACACAGTGGCATTACGTGCCATAATGT : 241

PxSE5 : -CACCTCTGACTACCCCTTCGGGGATTACAGTCGTGAGCATATGTATGT-------- : 389
SfSE1 : GCACCTCTGCCTACCCCTTCGGGGATTAAAGGCGTGACGATATGTATGTATGTATGT : 298

**Figure S4**
