# Supplementary material for: Diversity of short interspersed nuclear elements (SINEs) in lepidopteran insects and evidence of horizontal SINE transfer between baculovirus and lepidopteran hosts
Source: BMC Genomics. 2021 Mar 31;22:226. doi: 10.1186/s12864-021-07543-z (PMC8010984; doi:10.1186/s12864-021-07543-z)
Supplement: Supplementary file 5 — Additional file 5: Figure S5. The 8 copies with high identity to PxLINE1.1 in P. xylostella (A) and the multiple sequence alignment of one copy (AHIO01028576.1: 13049_14357) and Mariner-8_BM (B). [file 12864_2021_7543_MOESM5_ESM.docx]

AHIO01028576.1:13049_14357 : TTGCGCTACCTGAT--CCCGCTATAAAACCTTTCCA--CATCCGGTGTACCTTATCAGTCG-CTTGTTGCAAGTTG : 71
Mariner-8_BM : TTGCG--ACCTATTGACCC-CTATAAAAGCA--CCTGTTATCGGATTTTCTTTATCAGTCGACTT-TCACACGTTG : 70

AHIO01028576.1:13049_14357 : ACCGGTACACATCTC-GTTGCATTGTATTCCTTGTTTTCGCAAACCCATGGATACCACACCAGAAGAAGCTGCTCA : 146
Mariner-8_BM : ACCGGTACACATCTCCGCTACTTT-TGACCCTTTACCTTGTACGACAATGGAGACAACACCTACAGAAGCAGCACA : 145

AHIO01028576.1:13049_14357 : AGTTGTTGCCTTGCTGCAACAAGGGTTAAGTCAGCGAGCAGTCGCTGCCCAGCTCCACTTGAGCCAGTCTGCTGTA : 222
Mariner-8_BM : AATCGTAGCCCTATTGCAAGAAGGGCTCAGCCAGCGGGTTGTCGCTCGTCGGCTCCACATAAGCCAGTCCTGTGTT : 221

AHIO01028576.1:13049_14357 : TCCCGAGTATACAGACGGTTCCAAGAGACTGGTGCCTTCAATCGAAGACCAAGAACGGGCCGCCACCGCTGCACTT : 298
Mariner-8_BM : TCGAAAGCTTGCAAGCGCTTTCGGGAGACTGGTAGCTTTATCCCGAGACCAAGATCTGGACGGCGCCGGTGCACAT : 297

AHIO01028576.1:13049_14357 : CAGAGAGAGACGACCGCTTCATTGTCTCAACCTCGCTGCGCAATCGACACCTTACGGGCGTTGATGTGCAGCAAGA : 374
Mariner-8_BM : CGGAGAGGGATGACCGTTTTATCGTGTCAACCTCTCTCCGAAATCGGCATTTACCTGGTGTCGACGTCCAACAGGA : 373

AHIO01028576.1:13049_14357 : ACTGAGACGTGTACGACAAGTGGCTGTCAGCGAGTGGACAGTGAGAAGACGCTTGAAGGAAGCCAACTTGACACCA : 450
Mariner-8_BM : GCTCCGAGATGTTCGTGGGGTAGCAGCCAGCGAGTGGACAGTTCGTCGACGACTCAAGCAAGCGAATCTGACTCCA : 449

AHIO01028576.1:13049_14357 : AAAAGACCTGCATCAGGCCCCAAATTGACTGCAGGCCACCGACAAGCGCGTCTTCAGTTTGCTCGAGAGCATCTCG : 526
Mariner-8_BM : AAAAGGCCTGTCACAGGCCCGAAACTCACGGTAGCTCACCGACAAGCACGCCTTCAATTTGCTCGCACCCATCTTG : 525

AHIO01028576.1:13049_14357 : ATTGGAGCATTGCGCAATGGCGGTCGGTCCTGTTTACTGATGAGTGCAGAGTGTGTCTGCATGGCAGTGACAGGAG : 602
Mariner-8_BM : ATTGGGAGGTTGAGCAATGGAGGCAAGTCCTGTTCTCCGACGAGAGCAGGATGTGTTTGCACGGTAGCGACCGAAG : 601

AHIO01028576.1:13049_14357 : AGGCCGGGTCTACCGGCGTCCGGGGGAACGATTTGCCCAATGTTGTTTCGCTGAAACAGTAGCATATGGCGGCGGT : 678
Mariner-8_BM : AGGTCGGGTCTACAGGCGGCCTGGGGAGCGATTTGCGCAGTGCTGTTTCGCTGAAACAGTGGCTTATGGGGGCGGC : 677

AHIO01028576.1:13049_14357 : TCCTGCATGATGTGGGCTGGTATTTCTCTAGAGGGAAAAACCGCACTTGTTTTCGTGCCTGGAGGCGGCCGAGGAG : 754
Mariner-8_BM : TCTTGTATGATGTGGGCCGGCATTTCCTTCGACGGTAAAACCGAGCTTGTTTTCGTGCCTGGCGGGGGACGAGGAG : 753

AHIO01028576.1:13049_14357 : GCGGGTTAACAGCTGATCGGTACATCACCGACATTCTACTCGGTCATGTTGTGCCCTATGCAGAATTTGTCGGTGA : 830
Mariner-8_BM : GCGGTCTAACTTCGGACCGGTACATTTCCGATATTCTGCTGGAACATGTCGTTCCCTATGCGGGATATACCGGTGA : 829

AHIO01028576.1:13049_14357 : AGACTTCGTGCTAATGCACGACAATGCCCGCTGCCACACGGCACGAGTCAGTCGGCAGTTT--CT-GAGAGAGAAG : 903
Mariner-8_BM : TGACTTCCTCCTTATGCACGATAACGCTCGTTGTCACACTGCCCGTGTAA--CAACTGAATACCTCGAAGAAGTCG : 903

AHIO01028576.1:13049_14357 : GAATTGCGCACGATGGACTGGCCTGCGCTCAGTCCTGACCTGAATCCCATCGAACACTTATGGGACGAGCTCAAAA : 979
Mariner-8_BM : GTATCGC-CACATTGGACTGGCCTGCGCTCAGCCCTGACTTGAATCCTATTGAGCACGTGTGGGATGAACCGAAGA : 978

AHIO01028576.1:13049_14357 : GAAGAGTTCGGGCCAGGAATCCAGTCCCTGCAAGCGTGGACGAGCTGAAGACAGCTTTATTAGAGGAGTGGGACGG : 1055
Mariner-8_BM : GGAAGGTTCGTTCCAGAACTCCTGCTCCTTCATGTCTGAATGAGCTGAAATCGGCGTTGATTGAGGAGTGGGAAGG : 1054

AHIO01028576.1:13049_14357 : CTTTCCGTAGGAAACTGTCAAAAAGTTGATAAGGTCTATGAGAAACAGGCTGCAGGCTGTAATTAGGGCAAGAGGG : 1131
Mariner-8_BM : TATCCCACAAGAATCAATTCAGAAGCTGATCAGGTCTATGAAGAATCGTCTTCGAGCAGTTATTAGGGCGAGGGGA : 1130

AHIO01028576.1:13049_14357 : GGCAATACAAAGTATTGATTTAAATAAATAAATTTTTATCTTAACATTTTTTGT-TTAATTTGTATAATACGATAC : 1206
Mariner-8_BM : GGGAACACAAAGTACTGAC--ACTTAATTTTAAGGCTAAATAAAANTTTTATTTATTAATTTTTGTTGTT--TTAT : 1202

AHIO01028576.1:13049_14357 : CCATACCCT-TTTTTCCTAAATTCCCGTTTTTCCTACAAT-TGCGTTCAGACATCATTTATTTCAAAAATGATGAA : 1280
Mariner-8_BM : TCATTCATTATTTTTCCATATTTCCTTGTTTTCCTACCTCCTTCACTTAATCCGGGATAGCTCCTTAACTACTGGC : 1278

AHIO01028576.1:13049_14357 : TGGATTTCAATAATAATGATATCAAATTAAAGCTGACATCTTAAGCTTTTAAATGACATATCATTTTTATTAT--- : 1353
Mariner-8_BM : TCGATTTGAATGAATTTGGTATCAATTTAAAGTTGACAGTTGAACCTTTAAAATGA--TGTCATTTTTATGAT--- : 1349

**Figure S5**
