# Supplementary material for: Diversity of short interspersed nuclear elements (SINEs) in lepidopteran insects and evidence of horizontal SINE transfer between baculovirus and lepidopteran hosts
Source: BMC Genomics. 2021 Mar 31;22:226. doi: 10.1186/s12864-021-07543-z (PMC8010984; doi:10.1186/s12864-021-07543-z)
Supplement: Supplementary file 6 — Additional file 6: Figure S6. Alignment of potential LINE transposons in 8 lepidopteran insects genome. PxLINE1 in P. xylostella (NW011952036.1: 552486–555,713), TaLINE1 in Tuta absoluta (SNMR 01038797.1: 8533–11852), McLINE1 in Melitaea cinxia (APLT01012314.1: 14517–16103), CcLINE1 in Conopomorpha cramerella (SJJU01072145.1: 61771–65266), GmLINE1 in Galleria mellonella (NHTH01000021.1: 4230671–4228043), ArLINE1 in Adela reaumurella (WYDE01048472.1: 2507–535), AhLINE1 in Adoxophyes honmai (BHDV01006067.1: 48096–49728), DpLINE1 in Dendrolimus punctatus (JAABVI010000027.1: 8196917–8193378). [file 12864_2021_7543_MOESM6_ESM.docx]

PxLINE1 : --------------------------------------------------------------------------------------------------------------------- : -
TaLINE1 : --------------------------------------------------------------------------------------------------------------------- : -
McLINE1 : --------------------------------------------------------------------------------------------------------------------- : -
CcLINE1 : AAATCGATCTAGTGTGTCAGAGGGCTGTTTCTTGATTATATCAAGTCCGGTAAATCAAGAACAGTCACGGGTATGCGACCCCGGCCCCTCGAGCTATCGTCAGCACAAGCCTTGTCC : 117
GmLINE1 : --------------------------------------------------------------------------------------------------------------------- : -
ArLINE1 : --------------------------------------------------------------------------------------------------------------------- : -
AhLINE1 : --------------------------------------------------------------------------------------------------------------------- : -
DpLINE1 : --------------------------------------------------------------------------------------------------------------------- : -

PxLINE1 : --------------------------------------------------------------------AATTGTTTTTCATCTCTCAGTGCTCCGGCCCCTCCCGTGAGTAGCGGTA : 49
TaLINE1 : --------------------------------------------------------------------------AAGTTATATATTCTTCATCAGTCAGTTTATGCGCCGATGGATG : 43
McLINE1 : --------------------------------------------------------------------------------------------------------------------- : -
CcLINE1 : TTCCGTGCTGCGATATCATCACCCTTATTCCTCCTCCCAATTCCACGTACTTCCTTTCCCCACAACCTGATCCTTCATCCCATTCCGCCAATCCTTGGTCTGCCGCGCCTATGGATG : 234
GmLINE1 : --------------------------------------------------------------------------------------------------------------------- : -
ArLINE1 : --------------------------------------------------------------------------------------------------------------------- : -
AhLINE1 : --------------------------------------------------------------------------------------------------------------------- : -
DpLINE1 : ----------TGATTGAAACACGACAAGTACGTTATATATATACGTTTTGGTACAAGAGATGTCGCTTTATCAAGAATTTTATGGACAAGAATTTGAACCACAATCGACGATGTCTA : 107

PxLINE1 : CACCATTCATG----GTGCCGTGAAACCGATAACTACAC----------------------TCCAAGAGAACAGGG----------------------------------------- : 99
TaLINE1 : CATAGCTAGGGGC--GTGCCTAGCTTAGCGTGCCGATTT----------------------GCCCTGGGACCGGGTGACCCCAGATTACGGCTTACCTTGTCCTGGCAT-------- : 128
McLINE1 : --------------------------------------------------------------------------------------------------------------------- : -
CcLINE1 : CATGGCCAGGGGCGAGCCTGGTCGTAAGCGTGCCAACGT----------------------CCCGGATACCTGGCGACCTCCGGGCACTAATAAGCCTTGCCACATCAC-------- : 321
GmLINE1 : --------------------------------------------------------------------------------------------------------------------- : -
ArLINE1 : --------------------------------------------------------------------------------------------------------------------- : -
AhLINE1 : --------------------------------------------------------------------------------------------------------------------- : -
DpLINE1 : CATTTTTAATAGAAGTCGGTTCAAAATAGATGTCACTTTATGCAATGCTCTATATGACAAGTTCAATTAATCAGAATAATTCAAACCACAACCTACGGTATCCCCGCTTTTTATAAT : 224

PxLINE1 : --------------------------------------------------------------------------TTCAATATGGATCAACCCG------------------------ : 118
TaLINE1 : -------GCGGGGCTCTGCCAGGATCGACCTATTAATTCCCTTGCTACTCGTGGGAACGCA-ATGATTTCTTTACTTAACCCAAATCTAAATG------------------------ : 213
McLINE1 : --------------------------------------------------------------------------------------------------------------------- : -
CcLINE1 : -------GCGGGGCTCTGATGTGGTCGACCCTCTCTTTCCCCAGCTTTTCGTGGGACCCCAAATGAACAGTCTACCAAAATTCATTT------------------------------ : 401
GmLINE1 : --------------------------------------------------------------------------------------------------------------------- : -
ArLINE1 : --------------------------------------------------------------------------------------------------------------------- : -
AhLINE1 : --------------------------------------------------------------------------------------------------------------------- : -
DpLINE1 : ATTGATGTCGCTGCGATGATATGCTGTGATTTTTCTTTTTAAAGACTTTAAAACAATTAGTTATAATGTTTTAGTTTAAAAAAAAAAAAAATGTATAATATATAAATAAGTATTTAA : 341

PxLINE1 : ---------------------------------------------------------------------------GTGGTGGCGACGCGGTTCGCAACCCGTCACGCCGTGTCTCTC : 160
TaLINE1 : ---------------------------------------------------------------------------GCGGCGATGTAGGAGTTCGCGACTCATCACGTCTCGTTTCTA : 255
McLINE1 : --------------------------------------------------------------------------------------------------------------------- : -
CcLINE1 : ---------------------------------------------------------------------------GTAGCGGCGACGAGGCTCGCCACCCGTCACGTCAGGTCTCTC : 443
GmLINE1 : --------------------------------------------------------------------------------------------------------------------- : -
ArLINE1 : --------------------------------------------------------------------------------------------------------------------- : -
AhLINE1 : --------------------------------------------------------------------------------------------------------------------- : -
DpLINE1 : ATATGTATTAATATAGTGTAAGAAAGCCCATGAGAACACGCAATGGAATATATAGTGTATATAATAATAATCCGCACGGCGGCGACGCGGCTCGCAACTCGTCACGTCTCGTTTCTC : 458

PxLINE1 : GGAGGAGCGAAAAGTGGGCTGTGGAGACCCGCATAGCCACGATGAATATTTGTGGGGGTCTGGATGGAAAGATAGATGAAGTATGCGAGGTGTTTAACACAAGAGGAATGGATTTGC : 277
TaLINE1 : TCGGGAGCGGAACGCGGCCTACGGAGAGCCGCTTTGCTACGATGAATGTTAGAGGAGGAATGATAGATAAAATAGATGAGGTATGTCAGGTAATGGATGAAAGGCGTATAGATGTAT : 372
McLINE1 : --------------------------------------------------------------------------------------------------------------------- : -
CcLINE1 : GGAGAGGCGAAAAGTGGACTGCGGAGGCTCGATTTGCCACGCTGAATGTTTGTGGAGGATGGGACAGTAAGATTGATGAAGTTTGCAAGGTGATGAATGATAAGAATATTGATTTAA : 560
GmLINE1 : --------------------------------------------------------------------------------------------------------------------- : -
ArLINE1 : --------------------------------------------------------------------------------------------------------------------- : -
AhLINE1 : --------------------------------------------------------------------------------------------------------------------- : -
DpLINE1 : ATGGGGGCGAAAAGCGGACTGTGGAGAGCCGTTTGGCGACGTTGAATGTAAGAGGAGGGATGGACAGTAAAGTAGATGAAGTATGCCAGATGATGAATGAAAGAAAAATAGATTTAT : 575

PxLINE1 : TATGTGTGAATGAGTCCAAGCGGAAAGGTAGGGGAATTACCACGCACGGAACCTTGACAGCCTATTGGTCAGGGGTTCCTGAATCTGAGCATGGCTGTCAAGGGGTTGGGATTGTCC : 394
TaLINE1 : TGTGTGTGAATGAAACTAAGCGGAAAGGATGTGAAATGACGAAACATGGACCCTACACAGCGTATTGGTCTGGTGTTTCCAGTACCGATCGTGCATGTAAGGGAGTCGGCTTATTTC : 489
McLINE1 : --------------------------------------------------------------------------------------------------------------------- : -
CcLINE1 : TGTGTGTGAATGAGACGAAGCGAAAGGGTATAGACAGTACCACGCATGGCACGTACTCTGCGTATTGGTCGGGAGTGCCGCATTGCCAGCGTGCATGCCAAGGCGTTGGTCTTGTCT : 677
GmLINE1 : --------------------------------------------------------------------------------------------------------------------- : -
ArLINE1 : --------------------------------------------------------------------------------------------------------------------- : -
AhLINE1 : --------------------------------------------------------------------------------------------------------------------- : -
DpLINE1 : TATGTGTGAGTGAGACGAAGCGGAAAGGATGTGAAACCACTAAGTACGGTCAGTATACGGCTTATTGGTCAGGAGTGCAAGCGACTGAAAGAGGGAGTCAAGGGGTAGGTCTTATTC : 692

PxLINE1 : TATCTGAACGCTGGAATAATTGTGTGAAAGAATACGAGTGCGTGAGCCCAAGACTTATCTGGATCCGGTTAAAAGTAGGTTTGACGAGATTGTTTGTTCTGGGGGTTTATGCCCCG- : 510
TaLINE1 : TTTCCGCGCGAATGGCTGAATGTGTGACTGAGTTTGAATGTGTGAGCCCTCGACTCCTCTGGATTAGGCTAAAGGTTGGAATTACGCGAATCTTTGTCCTAGGTGCCTATGCGCCAA : 606
McLINE1 : --------------------------------------------------------------------------------------------------------------------- : -
CcLINE1 : TCTCAGAGCGAATGGCTGGTTGTTTGAAGAGCTACGAATGCGTGAACCCCAGACTCATATGGGCGAGACTCAAAGTTGGCATAAAGCACTTGTTCGTGTTGGGTGTATATGCACCG- : 793
GmLINE1 : --------------------------------------------------------------------------------------------------------------------- : -
ArLINE1 : --------------------------------------------------------------------------------------------------------------------- : -
AhLINE1 : --------------------------------------------------------------------------------------------------------------------- : -
DpLINE1 : TCTCTGAAAGAATGGAAGAATGTGTGAAAGAGCATGAGTGTGTCAGTCCAAGACTTGTATGGATTCGACTGAAAGTTGGCTTGACTCGGCTTTTTGTGGTTGGGGCATATGCACCAT : 809

PxLINE1 : -----------------GACACGTC---AAAATCTGCACAGGAGATAGATGAGTTTTGGAAAAGTATGAATGTAGTGTTGGATGAATGCGATGAGAATGAAAGGGTAATTATGCTCG : 607
TaLINE1 : CGGATTTGGGTACGGGTGGCTCCAC---AAAAGCTAAAAAGGAAAATGAGGAGTTCTGGGATAGTGTGAGAGAGGTTTTGAAAGGTTGTAATACAAATGAAAGGATTATCATGCTAG : 720
McLINE1 : --------------------------------------------------------------------------------------------------------------------- : -
CcLINE1 : -----------------GACATGTC---TAAGCCACTTAGAGAACGCGAAGAGTTCTGGGAGAATGTCAGGGGTGTGCTGTCAGAATGTAAGAGAAATGAGTTGATCATAGCGCTAG : 890
GmLINE1 : -------------------------------------------------------------------------------------------------------AATTATTTTGATAG : 14
ArLINE1 : --------------------------------------------------------------------------------------------------------------------- : -
AhLINE1 : --------------------------------------------------------------------------------------------------------------------- : -
DpLINE1 : CAGACATGAGCTCGACTTACACCTCCCGACAAACCATAGAGGAAAAGGACAAGTTTTGGGAATGTTTGAGAGGTGTGCTGCTTACATGTAAGCAGAACGAAAAATTAATTATGTTAG : 926

PxLINE1 : GTGATTTTAATAGTTGGGTAGGTGTGCAGCGTGATGGGTATGAAAGTGTATTGGGTAAGTTTGGTGACGTAAGAGTGAATGAGAATGGCAGGTATCTCCTGGATGTTTGCTTAGAAA : 724
TaLINE1 : GTGATTTAAATGGATGGGTAGGTATAAAGCGTGATGGGTATGATAAGGTTCTTGGAGCATTTGGAGACGAACGAGTTAATGAGAATGGAAAGAGTTTGTTAGAGTTTTGTTTGGAGT : 837
McLINE1 : --------------------------------------------------------------------------------------------------------------------- : -
CcLINE1 : GGGATTTTAATGGAAAAGTGGGTATACAGCGTGATTTGTATGAAAGTGTTCTGGGCTTGTTTGGTGATGAAAGAGTGAACGAGAACGGTGAGAGCCTTCTCGAGATATGTATGGAGT : 1007
GmLINE1 : GTGATTTTAATGGCTGGGTGGGAATCCGTCGCGATGGATATGAAAGGAATTTAGGAATGTTTGGAGATAAAAGAGTGAATGAAAATGGTGAAAGTTTATTAGAAGTATGCCTGGAGA : 131
ArLINE1 : --------------------------------------------------------------------------------------------------------------------- : -
AhLINE1 : --------------------------------------------------------------------------------------------------------------------- : -
DpLINE1 : GTGATTTTAATAGCTGGGTAGGCTTAAAACGAGAAGGTTATGAAAGGGTGTTAGGTACATACGGAGATGAAAGAATAAATATGAATGGTGACTGTTTGCTTGAGATATGTTTAGAAT : 1043

PxLINE1 : GAAACCTTATAGTTTCTAATTCTATGTTCAGGCACAAGGAGATACATATGTATACATGGCAACGACAGAATGAAAGGAGCATAATAGACTTTGTAATTGTGGATGAAAGGTTACGCG : 841
TaLINE1 : GGAATCTTCTTGTGTCGAATACAATGTTTCAACACAAGAAGATTCACACATATACAAGAGCAGAAGGTGTATCAAGGACCATTATAGATTTTGTGATTGTGGACGAAAGATTGAAGA : 954
McLINE1 : --------------------------------------------------------------------------------------------------------------------- : -
CcLINE1 : GGGACCTTGCTGTGACAAACACCATGTTTGATCACAGGAAGATCCACACGTATACTTGGCAGCGGGGTGTAGATAGAAGTATGATTGACTTTGTGATTGTGGATGGACGACTGAAGC : 1124
GmLINE1 : GAAATCTGGTGGTGACTAATACTTTGTTTAGTCACAAACTGATTCATATGTATACATGGCAAAGGCAAACTGAGAGAAGTATGATTGATTTTGTAATTGTGGATGAACGATTGAGAG : 248
ArLINE1 : --------------------------------------------------------------------------------------------------------------------- : -
AhLINE1 : --------------------------------------------------------------------------------------------------------------------- : -
DpLINE1 : GGAATTTGTGCGTGACTAACACAATGTTCTGTCACAAGAATATACATATGTACACGTGGGAAAATGTGAAAGGGAAAAGTATGATTGATTTTGTAATAGTAGATGAAAGATTGAGAA : 1160

PxLINE1 : TAAACGTTGCTGATACTCGAGTCTATCGGGGTGTCAATGTTGGAACGGACCATTTTCTGGTTGAAAGCCGGATTCGTGGTCTGTTTAATTATTGGCGACACCGACCTCGAGTATCAA : 958
TaLINE1 : AGAAAGTTCTAGACACTCGTGCGTATCGCGGGGCCGGGATCGACACAGACCACTTCCTGGTGATATCACGAATAAGTGGTCTCTTTACACGATGGCGACATAGAGTGCCCGAAGCGA : 1071
McLINE1 : --------------------------------------------------------------------------------------------------------------------- : -
CcLINE1 : AGCATGTGCTTGACACTAGGGTTTATCGTGGTGCTAATGTTGGGACCGATCATTATTTGGTTGTCAGCCGAATTAGTGGTTTGTTCCGGGGTTGGCGTCATCGGGCTCCTGTTGTCA : 1241
GmLINE1 : CGAAAGTGAAGGATACAAGAGTCTATCGTGGTACGAATGTCGGAACCGATCACTTTTTGGTGATATCGAGAATAGGTGGCTTATTCAATAAATGGCGACATCGTCCACGTGTTCGTA : 365
ArLINE1 : --------------------------------------------------------------------------------------------------------------------- : -
AhLINE1 : --------------------------------------------------------------------------------------------------------------------- : -
DpLINE1 : AAAACATTCTGGACACCAGATCATATCGGGGTACAGGCATCAATACAGATCACTTCTTAGTAGTGTGCCGAATAAAAGATCTTTTCAAAACTTGGAGGCACCAACATACCGATAAAC : 1277

PxLINE1 : CAACAAAT------TTAGAGCGTATCAAAGTAGAAAAATTGCAAGATGATGTTGTAAGTGAGGAGTATAGAAGAAGTTTGAAAGATAATTTAGAGAGCACTATT---GTCTTGGATG : 1066
TaLINE1 : CCAGTGTT------CTGGAAAGAGTGAAAGTAGAAAATTTGCAAGAAAAAGAGGTAAGAAAGGAATATGTCAAAAGGTTAAAGGATGAATTTGATAAGATGGAT---------GAGA : 1173
McLINE1 : --------------------ATAATTAAAGT-------------------------------------------------------------------------------------- : 11
CcLINE1 : ACACAGGC------TTGCAAAGAATAAAAGTTGATCAACTCCGGAAGGATGACGTGAAAGAGAAGTATGAATCAATGCTGAAAGAAAAACTCAGTATAGTGAATGAAGAAATAAGAG : 1352
GmLINE1 : TGTCAGAA------ATAGAAAGAGTAAGAGTAGAGAGACTGACAGAAGAACAGGTTCGAAATCAGTATCAAGAAAGATTGAATGAAAGATTTAGAGAGCTACTG---AAGAAAGATA : 473
ArLINE1 : --------------------------------------------------------------------------------------------------------------------- : -
AhLINE1 : --------------------------------------------------------------------------------------------------------------------- : -
DpLINE1 : ATAAATATAATACACTAGAAAGAATAAAAGTTGAAAAGTTGCAAGAAGAAGAAGTAAAAGAAGAGTATAAATCAAAATTACAAGAAAAATTTACTGAAATGAAT---------GATC : 1385

PxLINE1 : AAAATGATTTAGAAGAAAATTGGAAGCGCTTGAAAGATAGTTTAGTAAATACGGCGGTGAAAGTATGTGGAGTGAATAAAAGAAAGAAGAGTGGTAAGAGGGAGCTTACATGGTGGG : 1183
TaLINE1 : TGAATGATATTGAAGATGGTTGGAAGATATTTAAGGAAGTAATTGTCAAAGTTGCTGTTGAAGTATGTGGAGTCACTAGACGGGCAAAAGGTAATAAGCGAAAGACTGCATGGATGA : 1290
McLINE1 : --------------------------------------------------------------------------------------------------------------------- : -
CcLINE1 : AAGATGAGATAGATAATGTATGGAATGTTTTTAAAAATGGTATGGTTGAGATAGCCACTGAGGTATGCGGGGTGCGAAAGAGGAAGAATGTCTCCAGAGACTGTGAGCTCTGGTGGG : 1469
GmLINE1 : ACTATGAGATAGAAGAATTTTGGAAGGAATTTAAATGCGGAATCTTTGATATAGCTACTCAAGTTTGTGGTGTAAGTAAGAGGAAGGATGTTAAGATGAGAATGAATGTATGGTGGG : 590
ArLINE1 : --------------------------------------------------------------------------------------------------------------------- : -
AhLINE1 : --------------------------------------------------------------------------------------------------------------------- : -
DpLINE1 : TGTCCGAAATAGAAGAGTTGTGGATGTCCTTTAAAACTGAAGTTGTGGGTGTTGCGACTGATGTGTGTGGTATAACTAAAAGAAAAACGGGTAAATGCGTAAAGAATATTTGGTGGG : 1502

PxLINE1 : ATGATGAATGCAAAAAGGTAGTGAATGAAAAGAAAATGGCATGGTTGGATTTTTTGTCTAAAAAAGCCAACAACAGAATGCAAGGAAATCAGGGAATGGACGATGAAATGAAAGAGA : 1300
TaLINE1 : GTAAAGATGTGCAAGAAGTTGTTCAAAAGAAGAAAAAAGCATGGTTAGACTTATTGGCTACTAAAGCTAACCAAAGATTGCA--GA----AAGCGACAGATCAGGATGTGGAGAAAG : 1401
McLINE1 : --------------------------------------------------------------------------------------------------------------------- : -
CcLINE1 : ATGAGGAAGTAAGCAAGGGTGTGAATGAAAAGAAAAAGGCATGGATGGATGTGTTGGCCATGAGAGCCAAC---------------ACCCAAGCAACTAATGTGGATGAAAGTGAAA : 1571
GmLINE1 : ATGGCGAAGTGAAAGAAATGGTGGCTCTAAAGAAAAAGGCATGGATGGATTTGTTAGCTACCAGAGCTAATAAGAGAATGCA--------AGGTAATGTAAGTGACATTGAAAAAAT : 699
ArLINE1 : --------------------------------------------------------------------------------------------------------------------- : -
AhLINE1 : --------------------------------------------------------------------------------------------------------------------- : -
DpLINE1 : ATAAGGAAGTACAGAGTGTAGTGAATGATAAAAAGAAGGCATGGAAGGATTGGTTAGCTTTAAGAGCTAACCTAAGACTGAAAA------ATACAAAAAAGGAGGATGTAAATGAGG : 1613

PxLINE1 : TTCGAGAAA---AGTATGTTCTGCTAAAGAAAAAAGTAAAAGAAGTGATTGAAAGAAAGAAGAAGGCGTTAAAAGATGAATATGACAGGAAATTCTCTGACAACTTTCGAGCGAACA : 1414
TaLINE1 : CACGAAAGG---AGTATAAAAGTTTAAAGAATGTAGTAAAGAAGACAGTGGAACGAAAGAAAGAGGAAAATAAAGATGAGTTTGACAGGCGGCTATCAGAAAACTTTCGCACGAACT : 1515
McLINE1 : --------------------------------------------------------------------------------------------------------------------- : -
CcLINE1 : GGAAAATGTCTCAATATAAGTGCTTAAAGAAGAGAGTGAAAGAACTGATAGTACGTAAGAAGATTGAGTTAAAAGAGCGGAATGAAAAGAGGTTCTCTGACAACTTCCAAAATAATA : 1688
GmLINE1 : TGCAAAAGA-GAAGTATGATGTAATGAAAAAGAAAGTTAAAGAATTAGTAAGCAAAAAGAAAGAGGAATGTAAGGATGAGTATGAAAGGAAATTCTCTAGAAATTTTCGAGCTAATC : 815
ArLINE1 : ---------------------------------------------------AAAGAAATAAAAGGGAAGAGAGTGAAAAGAATGACCGGAGATTCTCTGACAACTTCCGGGCTAATA : 66
AhLINE1 : --------------------------------------------------------------------------------------------------------------------- : -
DpLINE1 : CAAAAATTT---TGTATAAAAGGATGAATGAAATAGTTAAAATTACAGTCAAAAAGAAAAAGAATGAGCAAAAAGATGATTTGGATGAAAGAATCTCTGAAGATTTCCAGAGTAACA : 1727

PxLINE1 : TTAAATTGTTCTGGAAGTTGGTAAGAAAGGCTCGAGGGAAGTCAGAGAATACAAATCTGGATGTGATAAGGGATGAAAATGGAGATGTTTTGAAAGATGAAAATAAAGTTCTTAAAA : 1531
TaLINE1 : TAAAGGTTTTCTGGAAATCTATAAAGACAACTCGAGGAAAAACAGCACAATCAGAGTTGAGTAGGATTCGGAGCCAGGATGGTAGTATAATAAAAGGTGAAGAATGTGTGTTGAAGA : 1632
McLINE1 : --------------------------------------------------------------------------------------------------------------------- : -
CcLINE1 : TTCGGCTATTTTGGAAACTAGTCCGGTCAGCCAGAAGTAAGTCAGAGAATCATAGCATGAAAGCAATTAGAGATAAGGATGGAAAAATTTTGAATGAAGAAAGTATGGTTGTAAGAA : 1805
GmLINE1 : AAAAGCTTTTCTGGCATTTAGTTCGAACAGCTAGAGGTAAAAAGAGAGATCCGAAAATGGGCGTGATAAGGGATATGGATGGTAATGTTATCACGGAACAAAAGGAAGTCTTAGATC : 932
ArLINE1 : TAAAACTATTTTGGAAAATGGTGAAAACAGCCCGGGGAAAATCAGAAACATCTAAAATGACTGATATCAGGGACGATCAGGGATGTGTTATAAAGGATGAAAAGAAAATTTTAGAAA : 183
AhLINE1 : ----ATTATTT-------------------------------------ATACAGAATCGAGAGTAACAGGGAATAACGATGGA---------------------------------- : 42
DpLINE1 : TTAAATATTTCTGGAAATCTATAAAAAGAGCTAGAGGAAAGACAGTACAGTCTGAGCTCAATGTAATCAGGGACAAGAACGGAGACATTGCGAAAGGAGAAAAATGCATATTGAACC : 1844

PxLINE1 : GATGGAAAGAATATTTTGAAAGTTTGTTTGAAAGCACAGATTGTAGAAC---------TAGTAGTGATGTAGAGGTAGAAAATGATAGAATAGTAGATGAGGAAAACAAGATAAGTA : 1639
TaLINE1 : GATGGAAGGAATATTTTGAAAGCTTGTTTGAAAAAGAAGAAGTACA------------AGAGGTGAACATACATGAAGAAAATGACAGTGAAAACGAAAG------CGAGATTGAAA : 1731
McLINE1 : --------------------------------------------------------------------------------------------------------------------- : -
CcLINE1 : GATGGAAGGAATACTTTGAAAATTTGTTTGAATGTAGCACCGCGTCGACTTTGGTTGATGCGGGTAATATGGATGGCGAAGAGAATAAAAG---------------TGAGATACTTA : 1907
GmLINE1 : ATTGGAAGAGATATTTTGAAAGTTTATTTGAACGTGGAAGTGAAAG------------AAAGAATGTTGAAAATGAAATAATGGACGAAGAAGTTGACAGTGAAAGTGATATAAGTA : 1037
ArLINE1 : GATGGAAGGATTATTTTGAAAGGCTCTTTGAACCTGAAGGAGTGGAGACACAGCCTGCAGAAGGAGCTGTGAGCGACACTCCTAGTGCGGGCAATGATAG------CGAGATAAGTA : 294
AhLINE1 : --------------------------------------------------------------------------------------------------------------ATTTGTA : 49
DpLINE1 : GCTGGAAAGAATATTTTGAAGGATTGTTTGAAAAAGATAAAGCGGA------------TGTGGAAAGTATGACTGATGAAAAGGACAATAGAGACGACAGTAGCGATAGTATAGGTA : 1949

PxLINE1 : TGAAAGAAATTATGGAAGCATTAAAAAGAATGAAAGTTGGTAAATCGGCCGGGTATGATAGGGTATCTTTAGAGATGCTAAGAGCGGGAGGTGGAGTGGCTGCAAGTGAGCTTTACC : 1756
TaLINE1 : TGGATGAGATTATGAAAGCTCTGAAAAGTATGAAATCTGGTAAGGCTGCAGGGTATGATAGAGTATCGGTTGAAATGCTGAAGGCTGGCGAAGGCGTAGCAGCTAGTCAGCTGTACC : 1848
McLINE1 : ---ATGAGATT-----AGCGCTGAAAAGTATGAGATTAGGTAAGGCTGCAGGGTATGACAGGATCACCGTCGAGATGCTGAGGGCTGGACAGGGTATAGTGGCTAGCCAGCTGTACT : 120
CcLINE1 : TGAATGAAATTTTCAAGGCTATGAAAGGAATGAAAAATGGAAAAGCGGCTGGGTATGATAGGATTTCGCTGGAGATGCTGAGGGCTGGTGGTGCGGTGGTGGCGAACCTGCTGTACC : 2024
GmLINE1 : TGGATGAAGTAATGAATGCAATGAAGATTATGAAATTTGGTAAAGCAGCGGGATTCGATAGAATATCTGCCGAAATGCTTAAAGAAGGTGGAGAAATCATTGTAAACCTGCTGTGTC : 1154
ArLINE1 : TGGACGAGATTCAGAAAGCATTGAAAAGCATGAAATCAGGTAAAGCAGCAGGATATGATAGAGTGTCTGTCGAAATGCTGAGGGGGGGTGGTTGGGTGGTTGCAGAACTGTTGCACC : 411
AhLINE1 : TAGAAGAAATAATGAAAGCGATGAAGGACCTGAAAGTAGGTAAAGCTGCTGGGTATGATAGTGTGACTGCGGAAATGCTAAAAGCTGGTGGTAGTTTGATGGCAGACTGGCTGTATC : 166
DpLINE1 : TGGATGAAATTATGAAAGCATTGAATAGTATGAAACTAGGAAAGGCTGCTGGGTATGATAGGATAACCGTGGAAATGCTAAAGGCTGGACAAGGGTTGGTCGCAAGTCTCTTATATC : 2066

PxLINE1 : AACTCTTCAATAAGTGCTGGCGTTGCGGTACTGTGCCACGCGACTGGTGCAGAGCGGTCATAGTTCCTTTGTATAAAGGAAAAGGCTCGCTCCAGACCTGCAACAGTTACCGCGGCA : 1873
TaLINE1 : TCCTTTTTAACCTTTGTTGGCGAAGCGGTCGAGTGCCAAGTGATTGGTGCAAGGCTGTAATAGTGCCATTATACAAAGGAAAAGGGTCACAGCTAGACTGTAAAAATTACCGCGGTA : 1965
McLINE1 : GCCTTTTCAATTTGTGCTGGCGAAATGGGCAAGTACCGGAAGACTGGTGCAAAGCCGTAATCGTGCCGTTGTATAAGGGAAAAGGGTCACGGCAGGACTGCATAAATTACCGCGGTA : 237
CcLINE1 : TTATTTTTAATGCATGTTGGCGATACGGCCGAGTACCAAGTGATTGGTGCAAAGCCGTAATCGTTCCACTGTATAAGGGTAAGGGCTCACAGCAGGATTGCAAACATTACCGAGGAA : 2141
GmLINE1 : TGCTGTTCAATAAATGTTGGCGGTCCGGTTGTGTTCCAGGTGACTGGAGCAAAGCTGTTATTGTGCCACTATATAAAGGCAGCGGGTCACAGCAGGAGTGCAAAAATTTCCGCGGAA : 1271
ArLINE1 : GGCTCTTTGATATGTGCTGGCGACATGGCCAAGTACCAGATGATTGGTGCAAAGCTGTGATCGTCCCACTATATAAAGGAAAAGGCTCGCAGCAGGAATGTAAAAACTACCGAGGCA : 528
AhLINE1 : GCCTCTTTAACCTGTGTTGGCAACAGGGCAAGGTTCCGGATGACTGGACCAAAGCAGTTATTGTGCCACTTTTTAAAGGGAAGGGCTCACAGCAAGAATGCAAAAATTATCGAGGTA : 283
DpLINE1 : GCCTTTTTAACATATGCTGGAAGAGTGGCTGTGTACCAAAAGATTGGTGCAAAGCCGTTATTGTACCACTCTTCAAAGGAAAAGGGTCAAAACAGGACTGTAAAAATTATCGGGGCA : 2183

PxLINE1 : TCAGCCTCTTGAGTATAGTTGGTAAATTGTATGCAAAAATATTGATTGAAAGGGTAGTGAAAGAGACGGAAGAAAAGATCTGGGATGTGCAAGGTGGTTTTCGAAAGGGGATGGGAT : 1990
TaLINE1 : TAAGCCTTCTCAGCGTCGTCGGTAAACTGTATGCAAGGGTACTGATTGAAAGAGTTGTGAAAGAAACTGATGAGAAAGTCTGGGATGCTCAAGCGGGATTTCGAAAGGGGATGGGAT : 2082
McLINE1 : TAAGCCTTCTCAGCGTCGTCGGTAAATTGTATGCGAAAGTGTTGATTGAAAGGGTTATGAATGAAACAGATGAAAAAGTATGGGATGCACAAGCGGGATTTAGAAAGGGAATGGGAT : 354
CcLINE1 : TCAGCCTTCTTAGTATCGTCGGCAAAATGTATGCAAAAGTGTTGATCGAAAGGGTAATACGTGAGACTGATGAAAAAATCTGGGATGTACAAGCTGGTTTTAGGAAGGGAATGGGAT : 2258
GmLINE1 : TAAGCCTTCTTAGTGTTGTAGGCAAATTGTATGCTAGGATTTTGATTAAAAGAGTGATGAATGAGACGGAAAGTAAAATTTGGGATGTGCAAGCAGGGTTTCGAAAGGGTATGGGAT : 1388
ArLINE1 : TAAGCCTTCTAAGTATTGTCGGCAAATTGTATGCAAAAGTACTGATTGAGAGGGTGTTGAATGTTACGGAGGAAAGGATCTGGGATGTGCAAACGGGATTTAGGAAGGGTATGGGAT : 645
AhLINE1 : TCAGTCTTTTAAGCATAGTAGGCAAGTTGTATGCGAGAATTTTGATTGAAAGAGTGATAAATGCAACTGAGAGCAGTATTTGGGATGTGCAAGCGGGTTTTAGAAAGGGAATGGGAT : 400
DpLINE1 : TCAGCCTTCTTAGCGTTGTCGGGAAAATGTATGCTAAAGTGTTAATTGAACGGGTATTGAAAGTAACAGAAGAGAAAGTATGGGATGTACAAGCGGGTTTTCGACAGGGAATGGGAT : 2300

PxLINE1 : GTACGGATCAAGTCTTTTCTCTACGAAGCGTCACAGAAAAGGTCCTTGCAAAGCAGCAAAAGGTTTTTTGTGCCTTCGTAGATTTGGAAAAGGCTTATGATAGAGTGAGGAGGAATG : 2107
TaLINE1 : GTACGGATCAGGTCTTTTCCTTGCGGTGCATAGCAGAAAAGTTTTTGGCTAAGAACCAAAAAGTCTTTTGCACATTCGTGGATCTGGAAAAGGCCTATGATAGAGTTCTGAGGAATG : 2199
McLINE1 : GTACGGATCAGGTCTTTTCCTTGCGGTGCATAGCCGAAAAGTTTTTAGCAAAAAACAAAAAGGTCTATTGCGCGTTCGTGGATCTGGAAAAGGCCTATGATAGAGTGGTGAGGAATG : 471
CcLINE1 : GCACCGACCAAGTCTTCTCTTTGCGCCAGGTGACGGAGAAGTTTTTGGCTAAAAACCAAAAGGTTTTCTGCGCTTTCATGGACTTAGAGAAGGCTTATGATCGGGTACCCAGGAATG : 2375
GmLINE1 : GTACGGATCAAGTCTTTTCCTTAAGGTGCATAACAGAGAAAACCTTAGCTCAACATCAGAAGGTTTTCTGCGCCTTCATTGACCTTGAAAAGGCTTATGATAGAGTGGAGAGGAATG : 1505
ArLINE1 : GCACGGATCTGGTCTTTTCCCTACGGTGCATAGCCGAAAAATTTCTGGCGACAAACCAGAAGATCTTTTGCGCGTTCGTAGATCTGGAAAAGGCCTATGACAAAGTGATGAGGAATG : 762
AhLINE1 : GTACGGATCAGGTTTTCTCATTACGGTGTGTAGCCGAGAAGTTCCTAGCGAAAGGCCAAAAAGTGTACTGCGCTTTCGTGGATCTTGAGAAAGCCTATGATAGAGTGAAGAGGGATG : 517
DpLINE1 : GTGTGGACCAAATCTTTACCTTACGGTGCATAACTGAAAAGTTTTTGGCCAAAGGTCAAAATGTGTATTGTGCATTTATAGATTTGGAGAAAGCTTATGATAGAGTGATAAGGAGTG : 2417

PxLINE1 : AATTATGGGA---GACACTGTCCGTTTATGGAGTGGACAGTCACCTGACGCGGGCACTGGGGTCCCTTTATAGGGAGTCTAGCGCTTGTGTCAGGATAAACGGAGCCTACACGGACT : 2221
TaLINE1 : AATTGTGGTC---GGCACTATCTGTGCATGGGGTGAGCAGCATCTTGGTACGAGCACTGAAATCCTTATACAAGGATTCCAGTGCTTGTGTCAGGATTAACGGGGCCTACACTGAGC : 2313
McLINE1 : AATTGTGGTC---AGCATTGTCCGTGTACGGTGTGAGCGGCGCACTCATGCGAGCACTACAATCTCTTTATAGGGATTCTAGTGCTTGTGTGAGGATAAACGGGGCATACACTGAAT : 585
CcLINE1 : AGTTGTGGACCTTGCTGCTGTCCCGGTTCAAGTTGGACAGGACCTTGGTACGGGCTTTGAAATCACTTTATGATGATTCCAGTGCCTGTGTCAGGGTAAACGGGACCTACTCAGACT : 2492
GmLINE1 : AACTGTGGTC---AACACTGGCAATGTATGGGGTGAGCAGCCATTTGATACGGGCATTCAAATCTTTATATAAGGATTCAAATGCCTGTGTCAGAATAAATGGAGTCTATACAGACT : 1619
ArLINE1 : AGTTGTGGGC---AGTGTTGTCCGAGTATGGAGTGGACAGTCACTTGGTTCGAGCACTGAAATCTCTCTATGAGGGATCAAAGGCCTGCGTCAGGATAAACGGGGCATACACCGACT : 876
AhLINE1 : AACTGTGGTC---AACGTTGTCCAGGAGAGGAGTGGATGGCGACTTGATACAGGCTCTGAAATCCTTATATAAGGATTCCAGAGCTTGCGTCAGATTGAACGGTGCCTATTCGGACT : 631
DpLINE1 : AACTATGGTC---AGCGCTAGCTAAGTATGGGATAACAGACAACCTGATACGATCACTGAAGTCCCTCTATAGCGATTCCAGTGCTTGTGTTAGGGTAAACGGCGCCTACACTGAAT : 2531

PxLINE1 : GGTTTGATATCCACAGGGGTGTTAGACAGGGATGTGTGGCTTCGCCTTGGCTGTTTAATCTGTTTATGGATAGTTGTCTCA-AGGATATGAAAGATGATGAAAGAGGTTTACGAATA : 2337
TaLINE1 : TGTTTAACATCGAGAAAGGTGTTAGACAGGGATGTGTTGCGTCACCATGGCTGTTCAACCTATTCATGGATAGTTGTTTGA-CAGATTTGAAAGAGTGTGAAAATGGACTGAGAATG : 2429
McLINE1 : GGTTTAATATCGAAAAAGGTGTTAGACAGGGATGTGTAGCGTCACCGTGGCTGTTTAACTTGTTCATGGACAATTGCTTGA-CAGAGTTAAAAGAGAATGAAAGTGGGTTGAGAATG : 701
CcLINE1 : GGTTTGGCATCCATAGAGGTGTCAGACAGGGATGCGTAGCGTCTCCGTGGCTGTTCAATCTGTTCATGGACAGTTGTTTGG-AAGATCTGAAAGGTCGTGATTGGGGTCTAGATATG : 2608
GmLINE1 : GGTTTGGCATTGAACGCGGTGTTAGACAGGGCTGTGTAGCGTCTCCTTGGCTGTTCAACCTATTTATGGACAGTTGCTTGACCCGATTTGAAAGGATCTAATTGTGGATTCAGGATG : 1736
ArLINE1 : GGTTTGACATCTCTAGAGGGGTCAGACAGGGGTGTGTGGCGTCACCTTGGCTGTTCAATCTATTCATGGACAGCTGCTTAG-CTGATCTAAAAGAATATGATTGTGGACTAAAAATG : 992
AhLINE1 : GGTTTGACATTTCTCGGGGTGTCAGACAGGGTTGTGTAGCATCGCCGTGGCTATTCAATCTTTTCATGGATGACTGTTTAC-TCGACTTAAAAGAGCGAGACTGTGGATTGAAGATA : 747
DpLINE1 : GGTTTAGCATTGAAAAAGGTGTTAGACAGGGCTGTGTAGCGTCGCCGTGGCTGTTCAACCTATTCTTAGACAGTTGCTTGG-ACGATTTGAAAGAAAGTGAAAGTGGTTTAAGAATG : 2647

PxLINE1 : GGAGAGTTACTTCTTAAGTGCTTGCTGTATGCTGACGATCAAGTCATACTTGCATCGTCGGTAGAACAGCTGCAACAACAAGTAACTCTCATGCATGAAAGTTTTAAAAGGAAAGGA : 2454
TaLINE1 : AATGGGTTACTCGTCAAATGTCTTCTCTATGCTGATGACCAAGTTATCCTTGCGTCGTCGGCTGAAGAGTTGCAGAAGATGGTAACAATTATGAATGAGGCTTTGAAAAAGAAAGGA : 2546
McLINE1 : GGTGAGTTACTCGTCAAATGTCTTCTCTATGCTGATGACCAAGTATTACTTGCGCCCTCGGCCGAGGAGTTGCAGGAGATGGTAACTGCTATGAGTGGAGCTTTTGTTAGAAAGGGA : 818
CcLINE1 : GGTGGTTTAATCATCAAATGCCTTTTATATGCTGACGATCAAGTTCTTCTTGCACCGTCGGCGTATGAATTGCAGCAGATGATTAATTGTATGAATGAGTCATTGAAAAGGAAAGGA : 2725
GmLINE1 : AATGAGTCTCTGTTTAAGACTCTTTTATACGCCGACGACCAAGTACTTTTTGCATCGTCGGAGTATGAATTACAACAAATGGTGACTATAATGAATGAAGCGTATGCAAGAAAAGGT : 1853
ArLINE1 : AGTGAGGTAGTTGTGAAATGCCTCTTGTACGCCGACGATCAAGTGATACTTGCATCGTCGGTGGACGAGTTGCAGGACATGGTAACTATTATGGTTGAAGCATTTAAAAGGAAAGGA : 1109
AhLINE1 : GGGGAGTCTCTCATCAAATGTCTCTTGTACGCCGATGATCAGGTGCTCCTTGCATCGTCGGCGTGTGAGTTACAGGACATGGTGACTCTAATGTACGAATCTTTTGAAAATAAGGGA : 864
DpLINE1 : AATGGGTTACTTATCAAATGTCTACTCTACGCCGATGATCAAGTTATAATTGCGTCATCGGAAACGGAGTTACAGGAGATGGTAACAATTATGAATGAGTCTTTTGAGAGAAAGGGT : 2764

PxLINE1 : ATGAAAGTGAATGTTAGTAAGACGAAAGTGATGGTGTTTGAAAGAGATGAAGAGGTAACTGAATGTGAGATCACGATCGAGAACGAAAGAGTGGAGCAAGTGAATGAGTTTGTATAT : 2571
TaLINE1 : ATGAAAGTGAATGTAAGTAAGACGAAATCAATGGTTTTTGAAAAAGAAGAAGGTATGACGGAGTGTAACATTATGATAGAAGAAGAGAGGGTGGAGCAAGTGAAAGAGTTTGTATAT : 2663
McLINE1 : ATGAAGATGAATGTAAAGAAGACGAAAGTGATGGTGTTTGAAAGAGATGAGGTAGTGACAGACTGTAATATCGTGATTGGAGATGAACAAATTGAACAGGTGAATGAGTTTGTGTAT : 935
CcLINE1 : ATGAAAGTGAATGCTAGTAAGACCAAAGTAATGGTAGTCGAAAATAATGAAAG---GACAGTTTGTGAGTGCATGATTGACGGTGAGAAAGTTGAACAAGTGGATGAGTTTGTATAT : 2839
GmLINE1 : ATGAAAATAAATGCGAAGAAGACGAAGGTAATGGTTTTTGAGAGGAATGAAAGTTTAACGGCATGCCAGATTATGATAGGAGATGAAAAAGTGGAGCAAGTGAGTGAGTTTCTATAT : 1970
ArLINE1 : ATGAAAGTGAATGTAAATAAGACGAAAGTAATGGTACTGGAGAGGAGTGAAATGATGTCAGAATGCAATGTAATGATTGGGGATGAGAGGCTGGAACAAGTGAACGAATTCGTATAT : 1226
AhLINE1 : ATGAAAATGAATGTAGGTAAAACGAAAGTTATGGTGATAGAGAAGAACGACGAGATGACTGAATGTATTATAACAGTTGGAAGTGAAAGATTAGAACAAGTGAGAGAGTTTAGATAT : 981
DpLINE1 : ATGAAAATTAATGTGAACAAGACGAAAGTTCTGGTGTTTGAAAGAGATGAAAGAATGACAAGTTGTAATATACAGATAGGAAATCAAAAGTTAGAGCAAGTTAGTGAATTTGTTTAC : 2881

PxLINE1 : TTGGGTAGTCTGTTTACGAGGGATGGGAAATGTGAGGGGGATATTGAAAGAAGAGTGAAAGCGGGAAATAAAGTGAATGGGGCCTTGCACTCTTTCATGGGGAGTCAAAGCGTGTCT : 2688
TaLINE1 : TTGGGATCGAAGTTTACATCAGATGGAAAGTGTGAAAGTGATATTGAAAGGAGGGTAAATGCCGGCAACATGGTGAATGGAGCGTTGCATGCCTTTATGAATAGCCAGACAGTGTCT : 2780
McLINE1 : CTGGGTTCGAAGTTTACAAGAGATGGAAAGTGTGAGAGTGATATTGAAAGAAGAGTGAATGCAGGAAACATGGTGAACGGAGCTTTGAACTCCTTTATGAGCAGTCGGAAGGTGTCT : 1052
CcLINE1 : TTGGGATCTCTATTTACAAGTGATGGAAAGTTTGACAAAGATATTGAAAGAAGAGTGAGTGCGGGTAATAAGGTTAACGGTGCCCTTCGCAGTGTTATGGAGAGCCAATTCATGTCG : 2956
GmLINE1 : TTAGGTTCTTTGTTTACGAGGGATGGGAAGTGTGAAAGAGATATTGAGAGGAGAGTAAAAGCGGGAAACAGTGTTAACGGTGCACTGCTCTCGATTATGTCAAGTAAAAATGTATCG : 2087
ArLINE1 : CTGGGAAGTATGTTCACGCGGGATGGAAAATGTGACGGAGACTTGGAAAAGAGAGTGAAAGCCGGTAATCAAGTGAACGGGGCGTTGCACTCGATTATGAGTAGCCAAAGTATGCCC : 1343
AhLINE1 : TTGGGATGCATGTTCACGAGTGACGGTAAGTACGATGAGGATATTGAAAGAAGAGTGAAAGCGGGAAATCAAATGAATGGAGCACTTCACTCAGTTGTGAGCCGTCAGGAAATTTCT : 1098
DpLINE1 : TTAGGCTGCATGTTTACCAGAGACGGAAAGCATGAAAGGGATATTGAAAGGAGAGTTATGGCAGGTAATAGGGTGAATGGAGCGCTAAATTCTTTTATGAATAGCCAGAAAGTGTCT : 2998

PxLINE1 : CAAAAGGCTCGTTTGGCTGTTCATGGGGGAGTGTTGGTCCCTACGCTAATGTATGGAAGTGAAAGCTGGGTCTGGCAGAAGAAGAATGAAAGTAGGGTAAATGCTGTTGAGATGCGC : 2805
TaLINE1 : AATAAGGCACGTCTGGCTGTGCATAGAGGAGTGTTAGTTCCAACACTTATGTATGGGAGTGAAAGTTGGGTATGGCAGAAAAAGCATGAAAGTAGAATAAATGCGGTAGAAATGAGA : 2897
McLINE1 : AAAAAGGCTCGTTTGGCTGTGCATGAGGGGGTGTTGCTTCCGACACTCATGTACGGAAGTGAAAGTTGGGTATGGCAGAAGAGACATGAAAGCAGAATAAATGCAGTTGAGATGAGA : 1169
CcLINE1 : AAAAAGGCAAGATTGGCTGTCCATGACGCTGTACTGGCACCTACCTTAATGTACGGGAGTGAAAGTTGGGTATGGCAGAAGAAGCATAAAAGTAAAGTCAATGCCGTAGAAATGCGG : 3073
GmLINE1 : AGCAAAGCTCGCCTTGCTATAATTCAAGGAGTACTGACACCAACACTCATGTATGGAAGCGAATCGTGGGTATGGCAGAAGAAGCATGAAAGTAAGATAAATGCTGTACAGATGAGA : 2204
ArLINE1 : AATAGGGCACGTTTGGCTGTCCATGGCGGAGTGCTGGCCCCTACATTGATGTACGGGAGTGAAAGTTGGGTATGGCAGACGAAGCATGAAAGCACGATAAATGCTGTGGAAATGCGC : 1460
AhLINE1 : AAGAATGCTCGTCTGGCTATTCACAACGGTGTGCTTGTGCCTACTTTGATGTATGGCAGAGAGAGTTGGGTGTGGCAGAAGAAACATGAAAGTAGAGTAAATGCGGTTGAGATGCGT : 1215
DpLINE1 : GTAAAGGCACGCTTGGCTGTCCATAAAGGAGTGTTGGTGCCGACCCTGATGTATGGTAGTGAAAGTTGGGTATGGCAGAAGAAGCATGAAAGTAGGATAAACGCAGTTGAAATGAGA : 3115

PxLINE1 : TCTCTAAGAAGTATGTGTGGACTGAAACTGAATGATAAGATAAGGAATAGTATTATAAGAGAGCGAGTTGGAGTAAAAGAAGACGTAGTGACCAAAATTGAAAAAGGAATGTTGAGA : 2922
TaLINE1 : GCGCTAAGAAGTATGATTGGAGTGAAATTGAGTGACAGGATAAGGAACAGTGTGATTAGAGAACGTTGCGGTATAAAAGAAGACGTAGTGACTGGTATAGAAAAAGGAATGCTGAGA : 3014
McLINE1 : GCGTTAAGAAGTATGATAGGAGTTAAACTGAGTGATAGGATGAGAAATAGTGAGATAAAGAAACGGTGTGGTCTGAAAGAAGATGTAGTGACAAAAATTGAGAAAGGTATGCTGAGA : 1286
CcLINE1 : TCATTGCGGAGCATGAAAGGCGTGACATTGAATGACAGGATAAGGAATAGTGTGATAAGGGAAAGTTGCGGTTTGAAGGAAGACGTAGTGACCAAGATTGAAAAGGGCATGCTTCGG : 3190
GmLINE1 : GCTTTGAGAAGTATGTGTGGTATTACTTTAAAAGATAGAGTAAGAAACAGTGTGATTAGAGAGAAGTGTGGACTGAAAGTCGATGTAGTGACCAAAATTGAAAAAGGAATGTTGCGT : 2321
ArLINE1 : GCGTTGAGGAGTATGTGTGGAGTCACAATGGAAGATAGATTGAGAAATAGTGTAGTGCGAGAGCGTTGTGGTCTGAAAGAAGATATAGTGACAAGGATTGAGAAAGGAATGGTTCGG : 1577
AhLINE1 : TCTTTGCGAAGCATGAGTGGTATCAAACTTACTGATAGGGTGCGTAATAGCGTGATTAGAGAGAAGTGTGGTCTGAAAGAGAGTATTATAACACGGATAGAGAAGGGTATGCTGCGT : 1332
DpLINE1 : GCTTTGAGAAGTATATGTGCGGTTAGCTTGAATGATAGGATAAGAAACAGTGTGCTAAGGGAACAATGTGGTTTGAAAGAAGATGTAATAACAAGAGTTGAAAAAGGAATATTAAGG : 3232

PxLINE1 : TGGTTTGGTCACATTGAAAGGATGGATGAAAGAAGACTGACGAAAGAAATTTACTGTGCAGAGATGAATGGTTGTGTCGGTAGAGGACGTCCTAGGCGAAAGTATGTCGACCAGATA : 3039
TaLINE1 : TGGTTTGGTCACATTGAGAGAATGAATGAAAGAAGATTAACGAAACAGGTGTATATGGCGAGTGTGGATGGGAACGTTGGAAGGGGAAGACCAAGACGCACGTACTTGGATCAGATC : 3131
McLINE1 : TGGTTTGGTCATGTCGAGAGAATGAATGAAGAACGACTGACGAAAAAAGTGTATAAGGCGAGTGTGAGTGGAAGTGTTGGAAGGGGTAGACCTAGGCGGACATTTCAAGACCAAATC : 1403
CcLINE1 : TGGTTTGGGCATATGGAGAGAATGGATGAAAGTCGATTGACGAAAGAGATTTATATGGGTGATGTAATGGGCCGGGTGAGTAGAGGTCGCCCCAGAAGGACATATAAAGACCAGATT : 3307
GmLINE1 : TGGTTTGGTCATTTGGAGAGGATGGATGAAAGAAGAATTACGAAAGAGATTTATCGAGCAAGAATGAATGGAGATGTCGGTAGGGGCCGCCCTAGACGGACGTATGTCGACCAAATA : 2438
ArLINE1 : TTGTTCGGTCATGTTGGAAGAATGGATGATAGGAGACTGACAAAAGAAATTTATGGAGCGGATGTGAGTGGAGGAGTCGGGAAGGGTCGCCCTAGACGCACGTATCAAGACCAGATC : 1694
AhLINE1 : TGGTTTGGGCATGTGGAGAGAATGGATGAAGCAAGGATATGTAAGCAAATTTACAAGGGAAGTGTGTTTGGGCAAGTCGGTCGAGGGCGCCCCCGAAGAACGTATATCGACCAGATC : 1449
DpLINE1 : TGGTTTGGGCATGTAGAGAGGATGGATGAAAATAGAATCACAAAACAGGTATATGACAGTATTGTCAATGGTAGAGCTGTTGCCGGTAGACCGAGACGAACATACCATGAACAAATA : 3349

PxLINE1 : GGCGACATACTCAAGAAGAGCCAAATTAGGAGTTTCCGAAACCGACGTGCGTGTATGAAGAGACTAATGAATGTTGAGGAAGCGAAAGAAGTATGTCAGGATCGTGGCACGTGGAGA : 3156
TaLINE1 : GGGGACGTCCTAAAGAAAGGTCAGGTCAAGAGTACCCTAAACCGACGAGCATGTATGAACAGATTGATGCATGTTGAAGAAGCGAAAGAGGTATGTCAGGATCGTAGCAAGTGGAAA : 3248
McLINE1 : GGGGACGTCTTGAAAAAAGGCCAGGTCAAGAGTACCCTAAACCGACGAGCATGTATGAAGGGAATAATGAAAGTGGATGAAGCGAAACAAGTATGTAAGGATCGTAGCAAGTGGAAA : 1520
CcLINE1 : GGTGATGTCCTGACCGAAGGTGGCGTGAAAAGTACTCGAAACCGGAGGTCATGTATGAAAAGATGTATGAATGTAGATGAAGCGAAAGGGGTATGTCAGGATCGTGACAAATGGAGA : 3424
GmLINE1 : GGCGACGTGCTGAAGAAGGGCCAAATTAAAAGTACCCGTAACCGACGAGCGTGTATGACAAGATTGATGAATGTGGAAGAAGCGAAAGAGGTTTGTTTGAATCGAAGCATTTGGCGT : 2555
ArLINE1 : GGGCTTGTGCTGAAAAAGGGTCAAGTGCGCAGTACCCGTAACCGACGAGCTTGTATGAAGAACATCATGAGAGTGGAAGAGGCGGCAGAGGTCTGTCAGGATCTTGGCACGTGGAGA : 1811
AhLINE1 : GACGATGTACTAAAGGGAGGCCAAATTAGGAGTACCCGAAACCGTCGAGCATGTATGACGAGAGTTATGAATGTGAGTGAAGCAAGAGAAGTATGTCAGAATAGAAGCGAATGGCGC : 1566
DpLINE1 : TCAGAACTGTTAAAAAAAGGTCAGGTAAAAAGCATTTCAAATAAACGCGCATGTATGAAGAGGTTAATGAAAGTAGACGAAGCGAAAGTTGTTTGTCAGGATCGTCGCAAGTGGAGA : 3466

PxLINE1 : TCCATAGTCTCTGCCTACCCCTCTGGGAGACAGGCGTG-AGTATATGTATGTATGTATGT---------AATTGTTTTTCAT : 3228
TaLINE1 : GATGTAGTCTCTGCCTACCCCTATGGGAAAGAGGCGTG-ATTTTATGTATGTATGTATGT---------AAGTTATATATTC : 3320
McLINE1 : GAAGTGGTCTCTGCCTACCCCTACGGGAAAGAGGCGTG-AGTATATGTATGTATGTAT-----------AATTAAAGTA--- : 1587
CcLINE1 : TCTGTAGTCTCTGCCTACCCCTCTGGGAGACAGGCGTGAAGTGTATGTATGTATGTATGTA--------AATCGATCTAG-- : 3496
GmLINE1 : TCCATTGTTTCTGCCTACCCTAATGGGAGACAGGCGTGACAGATATGTATGTATGTATGTA--------AGTAAGTATTTTG : 2629
ArLINE1 : TCCGTTGTCTCTGCCTACCCCACTGGGAAACAGGCGTG-ACGCTATGTATGTATGTATGTATTTACTGGAAAAGAAAT---- : 1888
AhLINE1 : TCTGTTGTCTGTGCCTACCCCGCAGGGAATAAGGCGTGACGGTTATGT---TATGTAT-----------TATTTATACAGA- : 1633
DpLINE1 : TCCATAGTCTCTGCCTACCCCCATGGGAAACAGGCGTG-ATTTTATGTATGTATGTATGTAT-------GATTGAAACACGA : 3540

**Figure S6**
