# Supplementary material for: Diversity of short interspersed nuclear elements (SINEs) in lepidopteran insects and evidence of horizontal SINE transfer between baculovirus and lepidopteran hosts
Source: BMC Genomics. 2021 Mar 31;22:226. doi: 10.1186/s12864-021-07543-z (PMC8010984; doi:10.1186/s12864-021-07543-z)
Supplement: Supplementary file 7 — Additional file 7: Figure S7. The origin analysis of ObSE2. (A) the alignment of 75-bp fragment at 5′-end of ObSE2 and 72-bp tRNA-related region of D. melanogaster. (B) the schematic representation of structure of ObSE2. [file 12864_2021_7543_MOESM7_ESM.docx]

A

tRNA : GTC--CTGTGGCGCAATGGATAACGCGTCTGACTACGGATCAGAAGATTCCAGGTTCGACTCCTGGC-AGGATCG : 72
ObSE2 : AGCGATGGTAGCCTAACGGTTCAATAGTGCAACTCAGAATCCAACGATACCGGGTTCGATCCTAGGCTCCGCACC : 75
B


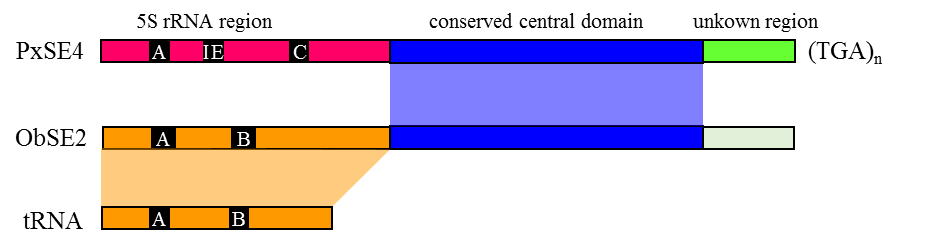


**Figure S7**
