# Supplementary figures and images for: Diversity of short interspersed nuclear elements (SINEs) in lepidopteran insects and evidence of horizontal SINE transfer between baculovirus and lepidopteran hosts
Source: BMC Genomics. 2021 Mar 31;22:226. doi: 10.1186/s12864-021-07543-z (PMC8010984; doi:10.1186/s12864-021-07543-z)

A


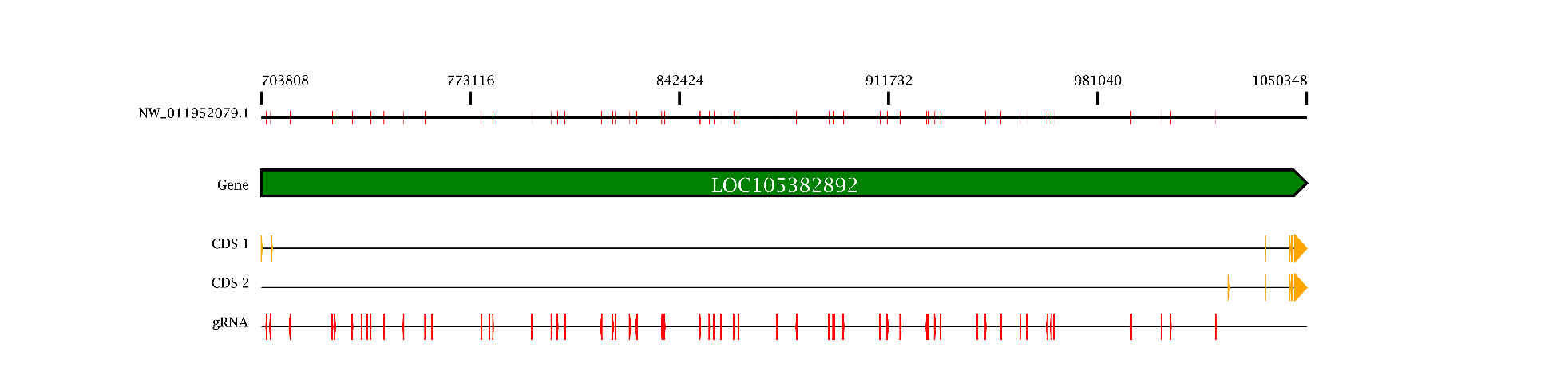


B


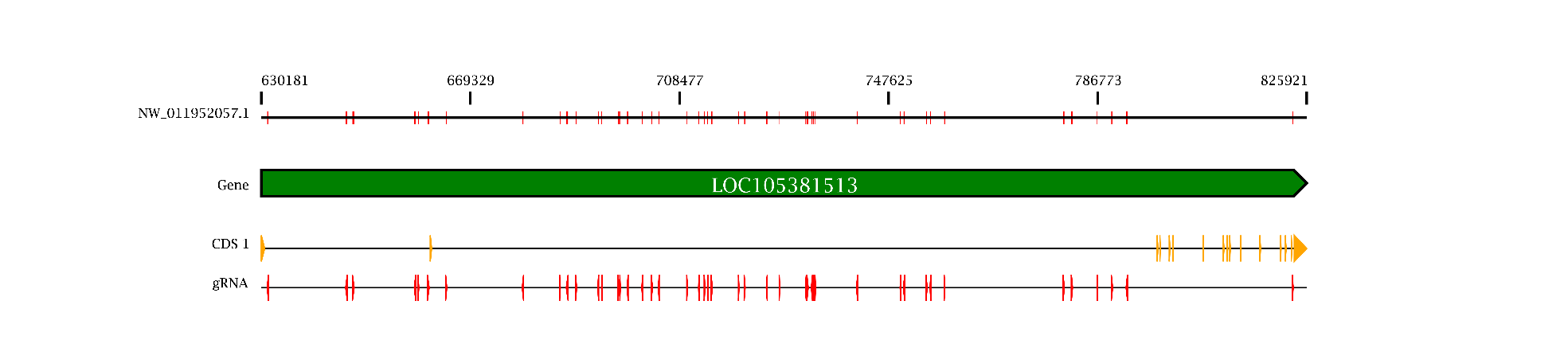


C


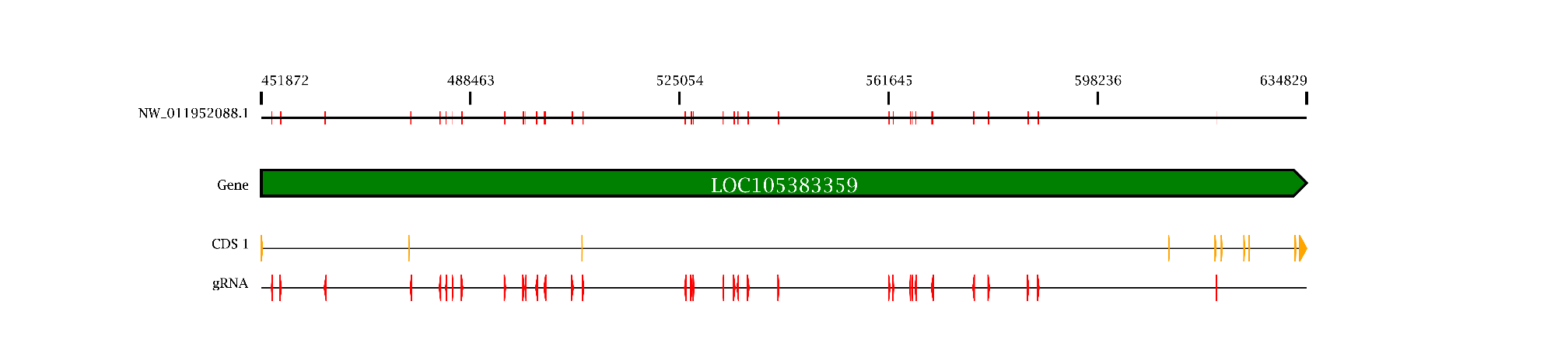


D

| gene  family | LOC105382892 | LOC105381513 | LOC105383359 | LOC105383464 | LOC105387706 | LOC105398356 |
| --- | --- | --- | --- | --- | --- | --- |
| PxSE1 | 18 | 17 | 11 | 12 | 6 | 7 |
| PxSE2 | 14 | 11 | 10 | 9 | 8 | 6 |
| PxSE3 | 10 | 7 | 2 | 4 | 4 | 9 |
| PxSE4 | 11 | 8 | 7 | 5 | 11 | 7 |
| PxSE5 | 7 | 2 | 4 | 3 | 3 | 2 |

**Figure S9**

Supplement: Supplementary file 10 — Additional file 10: Figure S9. The typical integration pattern of SINEs within genome of P. xylostella. (A) (B) and (C) are schematic diagrams of several copies inserted into the introns of LOC105382892, LOC105381513 and LOC105383359, respectively. (D) Statistics number of different SINE families inserted into the same gene. [file 12864_2021_7543_MOESM10_ESM.docx]
