# Supplementary material for: Diversity of short interspersed nuclear elements (SINEs) in lepidopteran insects and evidence of horizontal SINE transfer between baculovirus and lepidopteran hosts
Source: BMC Genomics. 2021 Mar 31;22:226. doi: 10.1186/s12864-021-07543-z (PMC8010984; doi:10.1186/s12864-021-07543-z)
Supplement: Supplementary file 11 — Additional file 11: Figure S10. Paralogous empty sites of PxSE1 (A) in P. xylostella and SfSE1 (B) in S. frugiperda. The nucleotides of TSD are indicated with the red background. The nucleotides of 3′ tail sequence are indicated with the gray background. [file 12864_2021_7543_MOESM11_ESM.docx]

A

NW_11952107.1_354375_354705 : ATTACGCTATTATGGCAATATGAATATAAAGACGACCGAATGGCGTAGTGGTTAGTGACCCTGACTACTGAGCCGATGGTCCCGGGTTCGATTCCC : 96
NW_11952215.1_331899_331959 : ATTACGCTATTATGGCAATATGAATATAAA------------------------------------------------------------------ : 30
NW_11952224.1_454937_454997 : ATTACGCTATTATGGCAATATGAATATAAA------------------------------------------------------------------ : 30
NW_11952259.1_525428_525488 : ATTACGCTATTATGGCAATATGAATATAAA------------------------------------------------------------------ : 30
NW_11952142.1_378405_378465 : ATTACGCTATTATGGCAATATGAATATAAA------------------------------------------------------------------ : 30
NW_11952058.1_9350_9410 : ATTACGCTATTATGGCAATATGAATATAAA------------------------------------------------------------------ : 30
NW_11952013.1_1882087_1882147 : ATTACGCTATTATGGCAATATGAATATAAA------------------------------------------------------------------ : 30
NW_11952396.1_231612_231672 : ATTACGCTATTATGGCAATATGAATATAAA------------------------------------------------------------------ : 30
NW_11952220.1_140716_140776 : ATTACGCTATTATGGCAATATGAATATAAA------------------------------------------------------------------ : 30

NW_11952107.1_354375_354705 : GGCTGGGGCAGATATTTGTTTAAACACAGATATTTGTTCTCGGGTCTTGGATGTGCCCGTAAAATGGCAATAGGCCCGCCCCCTATTACATTGGGA : 192
NW_11952215.1_331899_331959 : ------------------------------------------------------------------------------------------------ : -
NW_11952224.1_454937_454997 : ------------------------------------------------------------------------------------------------ : -
NW_11952259.1_525428_525488 : ------------------------------------------------------------------------------------------------ : -
NW_11952142.1_378405_378465 : ------------------------------------------------------------------------------------------------ : -
NW_11952058.1_9350_9410 : ------------------------------------------------------------------------------------------------ : -
NW_11952013.1_1882087_1882147 : ------------------------------------------------------------------------------------------------ : -
NW_11952396.1_231612_231672 : ------------------------------------------------------------------------------------------------ : -
NW_11952220.1_140716_140776 : ------------------------------------------------------------------------------------------------ : -

NW_11952107.1_354375_354705 : CTAACATAACACTCTGGCGAAAAGTGGGTGCAGCAATGCACCTCTGCCTACCCCGCAAGGGAGTACATTAGTACAAGGCGTGAGTGCGTGTGTGTG : 288
NW_11952215.1_331899_331959 : ------------------------------------------------------------------------------------------------ : -
NW_11952224.1_454937_454997 : ------------------------------------------------------------------------------------------------ : -
NW_11952259.1_525428_525488 : ------------------------------------------------------------------------------------------------ : -
NW_11952142.1_378405_378465 : ------------------------------------------------------------------------------------------------ : -
NW_11952058.1_9350_9410 : ------------------------------------------------------------------------------------------------ : -
NW_11952013.1_1882087_1882147 : ------------------------------------------------------------------------------------------------ : -
NW_11952396.1_231612_231672 : ------------------------------------------------------------------------------------------------ : -
NW_11952220.1_140716_140776 : ------------------------------------------------------------------------------------------------ : -

NW_11952107.1_354375_354705 : TGTGAATATAAAGTAATATTTGTATTGTATGTAAGTACTTAC : 330
NW_11952215.1_331899_331959 : ------------GTAATATTTGTATTGTATGTAAGTACTTAC : 60
NW_11952224.1_454937_454997 : ------------GTAATATTTGTATTGTATGTAAGTACTTAC : 60
NW_11952259.1_525428_525488 : ------------GTAATATTTGTATTGTATGTAAGTACTTAC : 60
NW_11952142.1_378405_378465 : ------------GTAATATTTGTATTGTATGTAAGTACTTAC : 60
NW_11952058.1_9350_9410 : ------------GTAATATTTGTATTGTATGTAAGTACTTA- : 59
NW_11952013.1_1882087_1882147 : ------------GTAATATTTGTATTGTATGTAAGTACTTAC : 60
NW_11952396.1_231612_231672 : ------------GTAATATTTGTATTGTATGTAAGTACTTAC : 60
NW_11952220.1_140716_140776 : ------------GTAATATTTGTATTGTATGTAAGTACTTAC : 60

B

NJHR_01000074.1_23878_23509 : TACGAATTTATAACAATTAAAAAAGAAACTGACTGCCTCGTTGGCCGAGTGGTTGCAAGTGCGACTGCCGGGCAAGGGGTCTCGGGTTCAAATCCC : 96
NJHR_01000656.1_88363_88303 : TACGAATTTATAACAATTAAAAAAGAAACT------------------------------------------------------------------ : 30
NJHR_01000768.1_116188_116128 : TACGAATTTATAACAATTAAAAAAGAAACT------------------------------------------------------------------ : 30
NJHR_01000750.1_2876_2816 : TACGAATTTATAACAATTAAAAAAGAAACT------------------------------------------------------------------ : 30
NJHR_01000750.1_4342_4282 : TACGAATTTATAACAATTAAAAAAGAAACT------------------------------------------------------------------ : 30
NJHR_01000752.1_63657_63597 : TACGAATTTATAACAATTAAAAAAGAAACT------------------------------------------------------------------ : 30

NJHR_01000074.1_23878_23509 : GGGTCGGGCGAAGTATTACTGGGCTTTTTTCGGTTTTTCGAAAATTTCTCAGTGGTAGCACGGAGTCTGGAAATGTGCCCGGTATATGGCAATAGG : 192
NJHR_01000656.1_88363_88303 : ------------------------------------------------------------------------------------------------ : -
NJHR_01000768.1_116188_116128 : ------------------------------------------------------------------------------------------------ : -
NJHR_01000750.1_2876_2816 : ------------------------------------------------------------------------------------------------ : -
NJHR_01000750.1_4342_4282 : ------------------------------------------------------------------------------------------------ : -
NJHR_01000752.1_63657_63597 : ------------------------------------------------------------------------------------------------ : -

NJHR_01000074.1_23878_23509 : CTCACCACCTATTACATGGGACTTAAAACATAGGTAAATTGTGAAAAGTGGGTGTACACAGTGGCATTACGTGCCATAATTTGCACCTCTGCCTAC : 288
NJHR_01000656.1_88363_88303 : ------------------------------------------------------------------------------------------------ : -
NJHR_01000768.1_116188_116128 : ------------------------------------------------------------------------------------------------ : -
NJHR_01000750.1_2876_2816 : ------------------------------------------------------------------------------------------------ : -
NJHR_01000750.1_4342_4282 : ------------------------------------------------------------------------------------------------ : -
NJHR_01000752.1_63657_63597 : ------------------------------------------------------------------------------------------------ : -

NJHR_01000074.1_23878_23509 : CCCTTCGGGGATTAAAGGCGTGACGATATGTATGTATGTAAAAAAGAAACTATATTACAACCTATCCTCTATGCTATAATA : 369
NJHR_01000656.1_88363_88303 : ---------------------------------------------------ATATTACAACCTATCCTCTATGCTATAATA : 60
NJHR_01000768.1_116188_116128 : ---------------------------------------------------ATATTACAACCTATCCTCTATGCTATAATA : 60
NJHR_01000750.1_2876_2816 : ---------------------------------------------------ATATTACAACCTATCCTCTATGCTATAATA : 60
NJHR_01000750.1_4342_4282 : ---------------------------------------------------ATATTACAACCTATCCTCTATGCTATAATA : 60
NJHR_01000752.1_63657_63597 : ---------------------------------------------------ATATTACAACCTATCCTCTATGCTATAATA : 60

**Figure S10**
